# Transcriptomic analysis of male and female *Schistosoma mekongi* adult worms

**DOI:** 10.1186/s13071-018-3086-z

**Published:** 2018-09-10

**Authors:** Orawan Phuphisut, Pravech Ajawatanawong, Yanin Limpanont, Onrapak Reamtong, Supaporn Nuamtanong, Sumate Ampawong, Salisa Chaimon, Paron Dekumyoy, Dorn Watthanakulpanich, Brett E. Swierczewski, Poom Adisakwattana

**Affiliations:** 1 Department of Helminthology, Faculty of Tropical Medicine, Mahidol University, Bangkok, Thailand; 2 Department of Microbiology, Faculty of Science, Mahidol University, Bangkok, Thailand; 3 Department of Social and Environmental Medicine, Faculty of Tropical Medicine, Mahidol University, Bangkok, Thailand; 4 Department of Molecular Tropical Medicine and Genetics, Faculty of Tropical Medicine, Mahidol University, Bangkok, Thailand; 5 Department of Tropical Pathology, Faculty of Tropical Medicine, Mahidol University, Bangkok, Thailand; 6 Department of Enteric Diseases, Armed Forces Research Institute of Medical Sciences, Bangkok, Thailand

**Keywords:** *Schistosoma mekongi*, Transcriptome, Differentially-expressed genes, Gene expression, RNA-Seq

## Abstract

**Background:**

*Schistosoma mekongi* is one of five major causative agents of human schistosomiasis and is endemic to communities along the Mekong River in southern Lao People’s Democratic Republic (Laos) and northern Cambodia. Sporadic cases of schistosomiasis have been reported in travelers and immigrants who have visited endemic areas. *Schistosoma mekongi* biology and molecular biology is poorly understood, and few *S. mekongi* gene and transcript sequences are available in public databases.

**Results:**

Transcriptome sequencing (RNA-Seq) of male and female *S. mekongi* adult worms (a total of three biological replicates for each sex) were analyzed and the results demonstrated that approximately 304.9 and 363.3 million high-quality clean reads with quality Q30 (> 90%) were obtained from male and female adult worms, respectively. A total of 119,604 contigs were assembled with an average length of 1273 nt and an N50 of 2017 nt. From the contigs, 20,798 annotated protein sequences and 48,256 annotated transcript sequences were obtained using BLASTP and BLASTX searches against the UniProt Trematoda database. A total of 4658 and 3509 transcripts were predominantly expressed in male and female worms, respectively. Male-biased transcripts were mostly involved in structural organization while female-biased transcripts were typically involved in cell differentiation and egg production. Interestingly, pathway enrichment analysis suggested that genes involved in the phosphatidylinositol signaling pathway may play important roles in the cellular processes and reproductive systems of *S. mekongi* worms.

**Conclusions:**

We present comparative transcriptomic analyses of male and female *S. mekongi* adult worms, which provide a global view of the *S. mekongi* transcriptome as well as insights into differentially-expressed genes associated with each sex. This work provides valuable information and sequence resources for future studies of gene function and for ongoing whole genome sequencing efforts in *S. mekongi*.

**Electronic supplementary material:**

The online version of this article (10.1186/s13071-018-3086-z) contains supplementary material, which is available to authorized users.

## Background

Mekong schistosomiasis is an important human parasitic disease and is caused by infection with *Schistosoma mekongi*. The parasite is highly prevalent along the Mekong River in southern Lao People’s Democratic Republic (Laos) and northern Cambodia [1–3]. Individuals infected by *S. mekongi* are mostly villagers living in endemic areas, but cases also occur in immigrants who resettle in other countries. Moreover, *S. mekongi* has been identified in international travelers who visited endemic areas, making this infection an important travel medicine issue in Southeast Asia [4–6].

As with other schistosome infections, disease develops after free-swimming *S. mekongi* larvae (cercariae) are released from a snail intermediate host, *Neotricula aperta*, and penetrate the human skin during swimming, bathing, farming and fishing [7]. Infection with *S. mekongi* is associated with a chronic local inflammatory response when eggs become entrapped in host tissues, resulting in intestinal disease, hepatosplenic inflammation and liver fibrosis [8]. No effective vaccine against *S. mekongi* is available, and anthelmintic treatment relies upon a single drug, praziquantel, against which resistance is becoming occurrence in some areas [9–13]. Thus, identification of novel targets for drug and vaccine development is urgently needed [14].

In previous studies, transcriptomic analyses of schistosomes have been conducted using expressed sequence tags (ESTs) [15–19], microarray hybridization [20–24] and serial analysis of gene expression [25–27]. However, data obtained using these technologies have limitations, including low throughput, short sequence length and resulting in challenges in assembly and the ability to analyze only known sequences. Recently, next-generation sequencing (NGS) technologies have significantly accelerated research in genomics, transcriptomics, epigenomics and metagenomics [28], which has dramatically improved the efficiency of gene discovery and gene expression analysis (RNA-Seq) in diverse species of plants and animals [29–33]. Transcriptomic analyses of human and animal schistosomes including *S. mansoni*, *S. japonicum*, *S. haematobium* and *S. turkestanicum* have been performed using RNA-Seq technology [34–43] and have contributed significantly to systematic efforts to identify gene targets. Despite recent advances in sequencing technology and bioinformatic methods, there is little information on the complete transcriptome of *S. mekongi*. Here, RNA-Seq technology and *de novo* assembly were used to provide a global view of the transcriptomes of male and female *S. mekongi* adult worms. The aim of our study was to produce transcriptomic data that would enhance our understanding of the biology of *S. mekongi* and facilitate the identification of candidate drug and vaccine targets*.*

## Results

### RNA-Seq and *de novo* transcriptome assembly

RNA-Seq analysis of *S. mekongi* adult worms using Illumina PE sequencing technology yielded a total of 346,331,720 and 411,906,616 raw reads from male and female worms, respectively. After quality assessment and trimming, 304,934,770 and 363,296,510 clean reads were obtained from male and female worms, respectively. Per-base quality distributions for each biological replicate are shown in Additional file 1: Figure S1.

Clean reads longer than 200 nt were used for *de novo* assembly. A total of 190,741 and 190,922 contigs with N50 values of 1844 and 1965 nt were generated from assemblies with k-mer sizes 25 and 31, respectively. The assembly with k-mer size 31 was subsequently selected, due to its high realignment rate and higher N50 value, to filter redundant sequences. Summaries of both transcriptome assemblies’ quality and realignment rate are shown in Additional file 2: Table S1. A total of 119,604 contigs were obtained in the final assembly with an average contig length of 1273 nt. The shortest contig was 201 nt long, while the longest contig was 30,465 nt long. The N50 length of the final assembly was 2107 nt. A total of 42,600 contigs were longer than 1 kb and 625 contigs were longer than 10 kb. The overall GC content was 34.1% (Table 1).

**Table 1:** Statistical summary of *de novo* assembled transcriptome sequences from male and female *S. mekongi* adult worms

Parameter	Male worms	Female worms
Summary of raw sequencing reads		
Total raw reads^a^	346,331,720	411,906,616
Total raw read nucleotides above Q30 (%)	48,771,315,768 (93.9%)	57,423,179,186 (92.9%)
Summary of trimmed sequencing reads		
Total clean reads^a^	304,934,770	363,296,510
Total clean read nucleotides above Q30 (%)	43,463,738,987 (97.4%)	51,257,988,579 (96.9%)
Summary of final transcriptome assembly contigs		
Number of contigs	119,604	
Smallest contig length (nt)	201	
Largest contig length (nt)	30,465	
Number of contigs with length < 200 nt	0	
Number of contigs with length > 1k nt	42,600	
Number of contigs with length > 10k nt	625	
N50 (nt)	2107	
GC content (%)	34.1	

^a^Reads of three replicates

### Functional annotation and GO classification

To annotate protein function, all 119,604 assembled contigs were subjected to prediction of protein CDS regions (Table 2). A total of 20,798 putative proteins were identified of which 19,744 could be annotated after BLASTP searching against the UniProt Trematoda database with an *E*-value cut-off < 10^-5^. Transcripts for which CDS regions could not be predicted were alternatively annotated using a BLASTX sequence similarity search against the UniProt Trematoda database with an *E*-value cut-off < 10^-5^. Following this analysis, 48,256 additional contigs could be assigned to functional proteins. All annotated transcripts produced from both analyses were further annotated using the GO databases of BLAST and Pfam. A total of 21,833 and 8462 assembled contigs were assigned to at least one of three GO terms of the BLAST and Pfam databases, respectively. GO assignments were grouped into three different categories: biological processes, molecular functions and cellular components, containing 8694, 16,366 and 11,055 transcripts, respectively.

**Table 2:** Summary of assembled transcript annotation

Transcript annotation	No. of transcripts
Total transcripts	119,604
Total protein sequences	20,798
Number of protein sequences with BLASTP hits	19,744
Number of transcript sequences with BLASTX hits	48,256
Number of transcript sequences with GO terms (BLAST)	21,833
Number of transcript sequences with GO terms (Pfam)	8462

### Analysis of differential expression

**Fig. 1:**
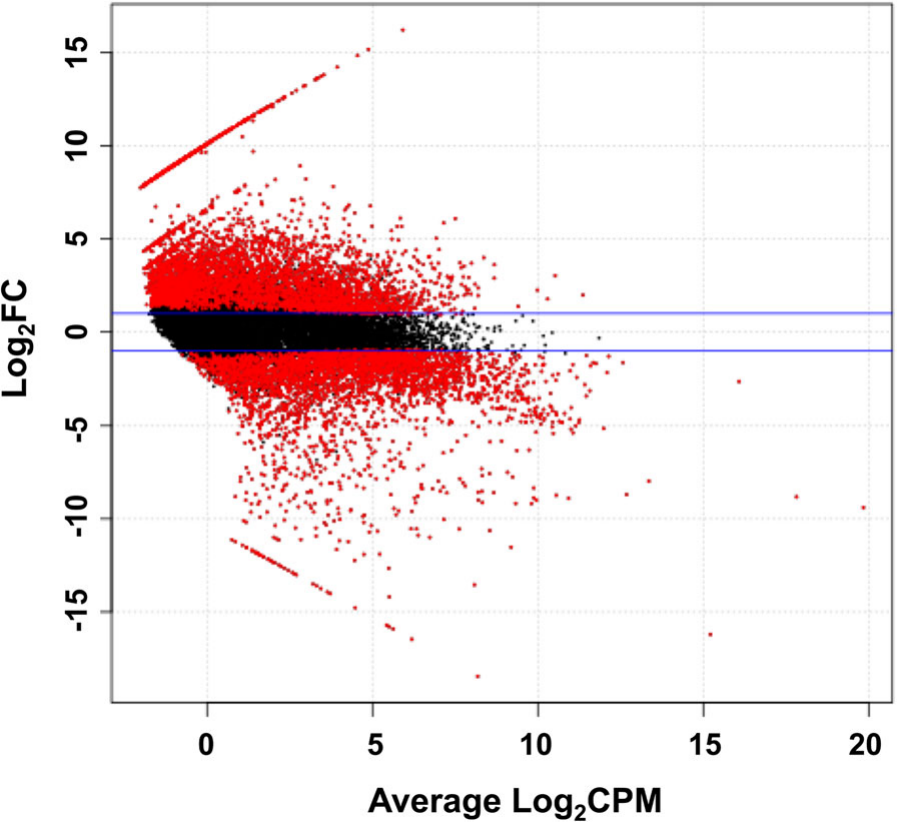
Smear plot of all DE transcripts with cpm > 1.0 from *S. mekongi* male and female worms. The graph shows average log_2_(cpm) on the x-axis *vs* log_2_(fold change in gene expression) between male and female worms on the y-axis. DE transcripts are shown in red and non-DE transcripts are shown in black

Of 119,604 total transcripts, 18,643 were DE in male and female adult worms with cpm > 1 (Fig. 1). In total, 8169 transcripts were significantly DE between male and female worms (FDR value < 0.05 and fold change > 2.0), spanning a log_2_ fold change range of -18.5 to 16.2. The red spots shown in Fig. 1 represent DE transcripts. Among all significantly DE transcripts, 4659 and 3510 transcripts were upregulated in male and female worms, respectively. The functional annotations of DE transcripts demonstrated that 2391 transcripts upregulated in male worms could be annotated but 2268 transcripts had unknown functions, while 1308 transcripts upregulated in female worms could be annotated but 2202 transcripts had unknown functions.

**Fig. 2:**
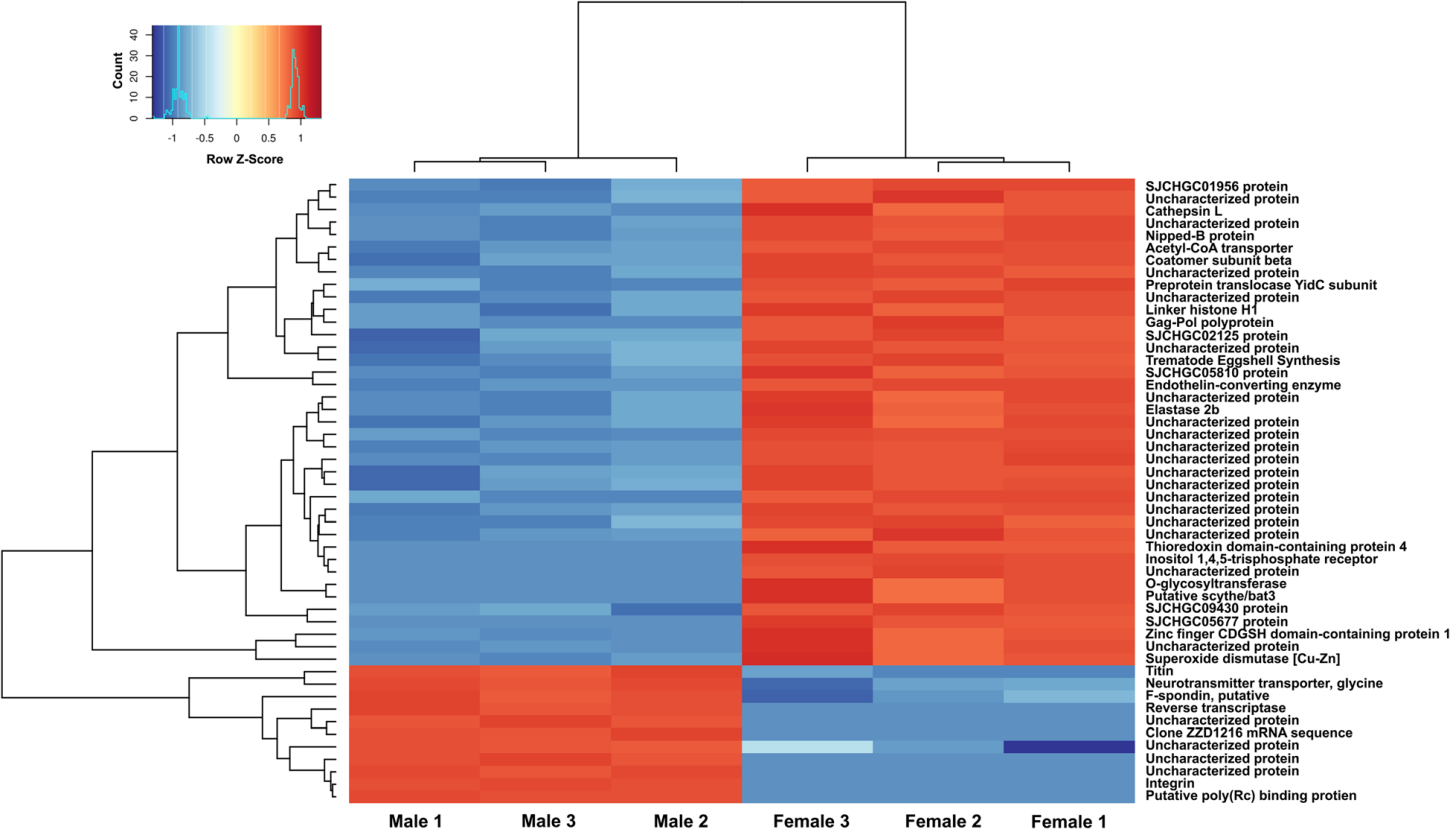
Heatmap of 50 most significantly DE transcripts from *S. mekongi* male *vs* female worms with three biological replicates. DE transcripts with higher expression levels are shown in red. DE transcripts with lower expression levels are shown in blue

The 50 most significantly DE transcripts of both male and female worms were shown in a heatmap (Fig. 2), with11 and 39 of these transcripts being significantly upregulated in male and female worms, respectively. However, 22 of these transcripts had unknown functions (4 and 18 transcripts in male and female worms, respectively). Transcript identification and protein descriptions are listed in Additional file 3: Table S2.

Among the significantly upregulated transcripts for each sex, the 50 transcripts with the highest upregulation are listed in Tables 3 and 4. In male worms, these were transcripts related to cytoskeleton-, motor-, and neuronal-associated proteins (dynein, tropomyosin 2, myosin light chain kinase and plexin A1) and to cell adhesion (integrin, CD97 antigen and serine-rich adhesin for platelets). Several transcripts associated with signaling pathways were also upregulated in male worms including serine/threonine kinase, receptor protein-tyrosine kinase, putative multiple ankyrin repeats single KH domain protein, serine/threonine-protein kinase PAK 3, rapamycin-insensitive companion of mTOR and regulator of G-protein signaling 3 (Table 3).

**Table 3:** Top 50 most significantly upregulated transcripts in *S. mekongi* male worms

Transcript ID	Protein description	Log_2_(FC)	*P*-value	FDR	Pfam annotation
comp65_seq18	Reverse transcriptase	16.2	2.66E-92	1.24E-88	–
comp2831_seq1	Uncharacterized protein	15.2	1.47E-70	2.29E-67	–
comp1481_seq2	Clone ZZD1216 mRNA sequence	14.8	1.19E-70	2.01E-67	–
comp926_seq0	Uncharacterized protein	14.2	3.84E-56	3.01E-53	–
comp2062_seq0	Uncharacterized protein	13.8	1.53E-45	6.63E-43	–
comp4508_seq0	Integrin	13.7	2.79E-47	1.30E-44	–
comp4099_seq6	Uncharacterized protein	13.6	3.96E-41	1.34E-38	G-patch domain, FtsJ-like methyltransferase
comp556_seq2	Hypothetical protein	13.6	3.35E-32	4.84E-30	–
comp4395_seq2	Putative poly(Rc) binding protein	13.6	1.45E-43	5.73E-41	KH domain
comp298_seq2	Uncharacterized protein	13.5	2.02E-41	7.11E-39	Ankyrin repeats
comp5387_seq1	Serine/threonine kinase	13.2	1.95E-36	4.39E-34	Protein kinase domain, protein tyrosine kinase
comp3544_seq2	Dynein-associated protein	13.2	1.51E-34	2.79E-32	–
comp510_seq4	Tropomyosin-2	13.0	1.04E-22	6.12E-21	Aida N-terminus, tropomyosin
comp1917_seq1	Lysine-tRNA ligase	12.8	2.43E-26	2.02E-24	tRNA anti-codon, OB-fold nucleic acid binding domain
comp7221_seq1	Uncharacterized protein	12.6	3.04E-27	2.75E-25	–
comp8398_seq0	Putative homeobox protein	12.6	6.23E-25	4.54E-23	N-terminal of Homeobox Meis and PKNOX1
comp3445_seq0	Receptor protein-tyrosine kinase	12.6	1.84E-26	1.55E-24	Receptor L domain, furin-like cysteine rich region
comp3797_seq0	Uncharacterized protein	12.5	2.10E-26	1.75E-24	BTG family
comp3182_seq1	Putative multiple ankyrin repeats single KH domain protein	12.4	2.95E-17	9.84E-16	Ankyrin repeats
comp115_seq0	Uncharacterized protein	12.4	1.44E-22	8.37E-21	Arrestin (or S-antigen), N-terminal domain
comp7837_seq2	Uncharacterized protein	12.2	8.70E-24	5.69E-22	Cadherin domain
comp5447_seq1	Myosin light chain kinase, smooth muscle	12.2	1.17E-15	3.10E-14	Immunoglobulin I-set domain
comp3754_seq1	Metastasis suppressor protein 1	12.1	3.29E-19	1.38E-17	IRSp53/MIM homology domain
comp8738_seq10	Uncharacterized protein	12.1	2.30E-21	1.20E-19	–
comp4029_seq0	Plexin-A1	12.1	4.14E-20	1.88E-18	Plexin repeat, IPT/TIG domain
comp138_seq8	SJCHGC08958 protein	12.0	1.54E-13	3.00E-12	Trematode eggshell synthesis protein
comp5826_seq0	Uncharacterized protein	12.0	2.10E-22	1.19E-20	Leucine rich repeat
comp3044_seq3	Uncharacterized protein	12.0	1.01E-20	4.89E-19	NIF3 (NGG1p interacting factor 3)
comp8147_seq2	Uncharacterized protein	11.9	4.28E-20	1.94E-18	Laminin G domain
comp595_seq0	Choline dehydrogenase, mitochondrial	11.9	7.87E-15	1.85E-13	GMC oxidoreductase
comp1402_seq1	Uncharacterized protein	11.9	1.25E-16	3.78E-15	–
comp490_seq3	Serine/threonine-protein kinase PAK 3	11.9	5.01E-19	2.07E-17	P21-Rho-binding domain
comp8255_seq1	Uncharacterized protein	11.8	3.00E-20	1.38E-18	–
comp4371_seq0	Putative ABC transporter	11.8	1.63E-19	7.01E-18	ABC transporter
comp5811_seq8	Rapamycin-insensitive companion of mTOR	11.8	2.32E-17	7.85E-16	Rapamycin-insensitive companion of mTOR, domain 5
comp9143_seq1	Regulator of G-protein signaling 3	11.8	5.29E-21	2.64E-19	C2 domain, PDZ domain (DHR or GLGF)
comp9843_seq1	Uncharacterized protein	11.8	6.13E-20	2.71E-18	Rhodopsin family
comp8166_seq1	Uncharacterized protein	11.8	4.38E-21	2.20E-19	–
comp1561_seq0	Uncharacterized protein	11.6	2.16E-12	3.55E-11	–
comp6197_seq1	Uncharacterized protein	11.6	3.52E-17	1.17E-15	–
comp9531_seq0	Putative ankyrin 2,3/unc44	11.6	2.07E-16	6.05E-15	Ankyrin repeats
comp9039_seq1	Centrosomal protein of 120 kDa	11.6	2.64E-19	1.11E-17	C2 domain, Cep120 protein
comp2906_seq3	CD97 antigen	11.6	3.96E-18	1.47E-16	Secretin family
comp3517_seq2	Rho guanine nucleotide exchange factor 12	11.6	1.38E-19	6.00E-18	Regulator of G protein signalling-like domain
comp2195_seq3	Uncharacterized protein	11.6	1.91E-15	4.93E-14	Galactoside-binding lectin
comp5487_seq0	Uncharacterized protein C7orf63-like protein	11.6	8.05E-20	3.52E-18	–
comp4205_seq4	Serine-rich adhesin for platelets	11.5	2.14E-14	4.76E-13	–
comp3759_seq1	Putative Alstrom syndrome protein	11.5	1.86E-16	5.50E-15	–
comp9022_seq1	Uncharacterized protein	11.5	1.97E-18	7.61E-17	MORN repeat
comp10754_seq0	Uncharacterized protein	11.4	8.21E-19	3.31E-17	Cadherin-like

**Table 4:** Top 50 most significantly upregulated transcripts in *S. mekongi* female worms

Transcript ID	Protein description	Log_2_(FC)	*P*-value	FDR	Pfam annotation
comp837_seq1	SJCHGC05677 protein	18.5	1.51E-88	4.69E-85	–
comp546_seq10	O-glycosyltransferase	16.5	6.50E-54	4.04E-51	Tetratricopeptide repeat
comp1_seq2	Uncharacterized protein	16.2	2.08E-09	2.06E-08	–
comp1141_seq3	Putative scythe/bat3 DNA repair	15.9	7.99E-51	4.51E-48	Ubiquitin family
comp401_seq5	SJCHGC09037 protein	15.8	7.56E-34	1.29E-31	Protein similar to CwfJC-terminus 1
comp7058_seq0	Uncharacterized protein	15.7	6.12E-20	2.71E-18	–
comp524_seq5	Uncharacterized protein	14.8	8.48E-19	3.41E-17	–
comp5822_seq2	DNA excision repair protein ERCC-6 DNA repair	14.2	7.21E-29	7.68E-27	Type III restriction enzyme, res subunit
comp5430_seq10	Inositol 1,4,5-trisphosphate receptor	14.0	5.20E-88	1.39E-84	Inositol 1,4,5-trisphosphate/ryanodine receptor
comp3416_seq1	Thioredoxin domain-containingprotein 4	13.9	1.32E-48	7.02E-46	Thioredoxin-like domain
comp3035_seq0	Uncharacterized protein	13.7	7.86E-27	6.85E-25	DnaJ domain
comp6618_seq1	Uncharacterized protein	13.6	3.75E-67	4.99E-64	–
comp247_seq3	SJCHGC09430 protein	13.5	2.43E-64	2.66E-61	–
comp3544_seq3	Mannosyltransferase	13.5	4.23E-24	2.84E-22	Alg9-like mannosyltransferase family
comp7684_seq1	Uncharacterized protein	13.0	2.85E-26	2.34E-24	–
comp1544_seq2	EH domain-bindingprotein 1	12.9	5.14E-22	2.83E-20	CAMSAP CH domain
comp4065_seq0	Uncharacterized protein	12.9	7.78E-31	1.00E-28	–
comp3239_seq2	Uncharacterized protein	12.8	1.94E-19	8.30E-18	Phosphatidylethanolamine-binding protein
comp167028_seq0	Uncharacterized protein	12.7	2.33E-64	2.66E-61	–
comp7307_seq0	Uncharacterized protein	12.7	1.97E-31	2.68E-29	–
comp6088_seq2	Uncharacterized protein	12.6	7.12E-39	1.92E-36	–
comp4607_seq0	Uncharacterized protein	12.5	7.98E-33	1.23E-30	–
comp8587_seq1	Pol polyprotein	12.4	2.50E-40	7.78E-38	–
comp5229_seq0	Uncharacterized protein	12.3	4.78E-15	1.16E-13	–
comp8246_seq0	Uncharacterized protein	12.3	4.60E-25	3.41E-23	–
comp2149_seq0	Uncharacterized protein	12.2	4.45E-97	2.77E-93	–
comp5198_seq1	Uncharacterized protein	12.2	1.16E-28	1.20E-26	–
comp475_seq2	Ccr4-not transcription complex gene regulation	12.2	1.52E-37	3.94E-35	–
comp8567_seq0	Uncharacterized protein	12.0	1.58E-31	2.17E-29	–
comp7704_seq0	Uncharacterized protein	12.0	7.90E-28	7.63E-26	–
comp1502_seq0	Uncharacterized protein	12.0	3.49E-20	1.59E-18	–
comp9595_seq0	Uncharacterized protein	11.9	4.66E-30	5.57E-28	–
comp29_seq2	SJCHGC05410 protein	11.9	1.22E-17	4.25E-16	–
comp5733_seq0	Elastase 2b	11.9	5.02E-60	4.92E-57	Trypsin-like peptidase domain
comp785_seq0	Uncharacterized protein	11.9	1.50E-22	8.68E-21	–
comp11219_seq0	Uncharacterized protein	11.9	3.03E-36	6.57E-34	–
comp9353_seq1	Uncharacterized protein	11.8	2.73E-21	1.40E-19	–
comp969_seq7	Uncharacterized protein	11.8	6.68E-27	5.93E-25	–
comp5698_seq0	Uncharacterized protein	11.8	1.13E-35	2.34E-33	–
comp10698_seq1	Uncharacterized protein	11.7	6.86E-34	1.18E-31	–
comp7614_seq1	Uncharacterized protein	11.7	2.55E-21	1.32E-19	–
comp4729_seq2	Uncharacterized protein	11.6	1.17E-30	1.45E-28	Alpha amylase, catalytic domain
comp10183_seq1	Uncharacterized protein	11.5	1.90E-21	1.01E-19	–
comp5834_seq0	SJCHGC06880 protein	11.5	2.91E-33	4.68E-31	RING-variant domain
comp268_seq0	Putative 26S proteasome non-ATPase regulatory subunit 11 protein misfolding repair	11.5	8.14E-05	3.44E-4	PCI domain
comp9969_seq1	Uncharacterized protein	11.4	2.39E-33	3.87E-31	–
comp1262_seq5	Uncharacterized protein	11.2	3.05E-22	1.70E-20	Dynein heavy chain
comp1218_seq2	Connector enhancer of kinase suppressor of Ras2	11.2	5.59E-30	6.52E-28	PDZ domain (DHR or GLGF)
comp5896_seq0	Uncharacterized protein	11.2	5.53E-90	2.06E-86	–
comp5214_seq0	Uncharacterized protein	11.1	2.20E-29	2.42E-27	–

Among the 50 most upregulated transcripts in female worms, transcripts with known function primarily related to glycosylation activity (O-glycosyltransferase, mannosyltransferase), peptidase activity (elastase 2b) and antioxidant enzyme activity (thioredoxin domain-containing protein 4). Moreover, several genes related to DNA- and protein-misfolding repair activity (scythe/bat3, DNA excision repair protein ERCC-6, 26S proteasome non-ATPase regulatory subunit 11), gene regulation activity (Ccr4-not transcription complex) and cellular and physiological control activity (inositol 1,4,5-trisphosphate receptor) were upregulated (Table 4).

### GO and KEGG pathway analyses of DE transcripts

**Fig. 3:**
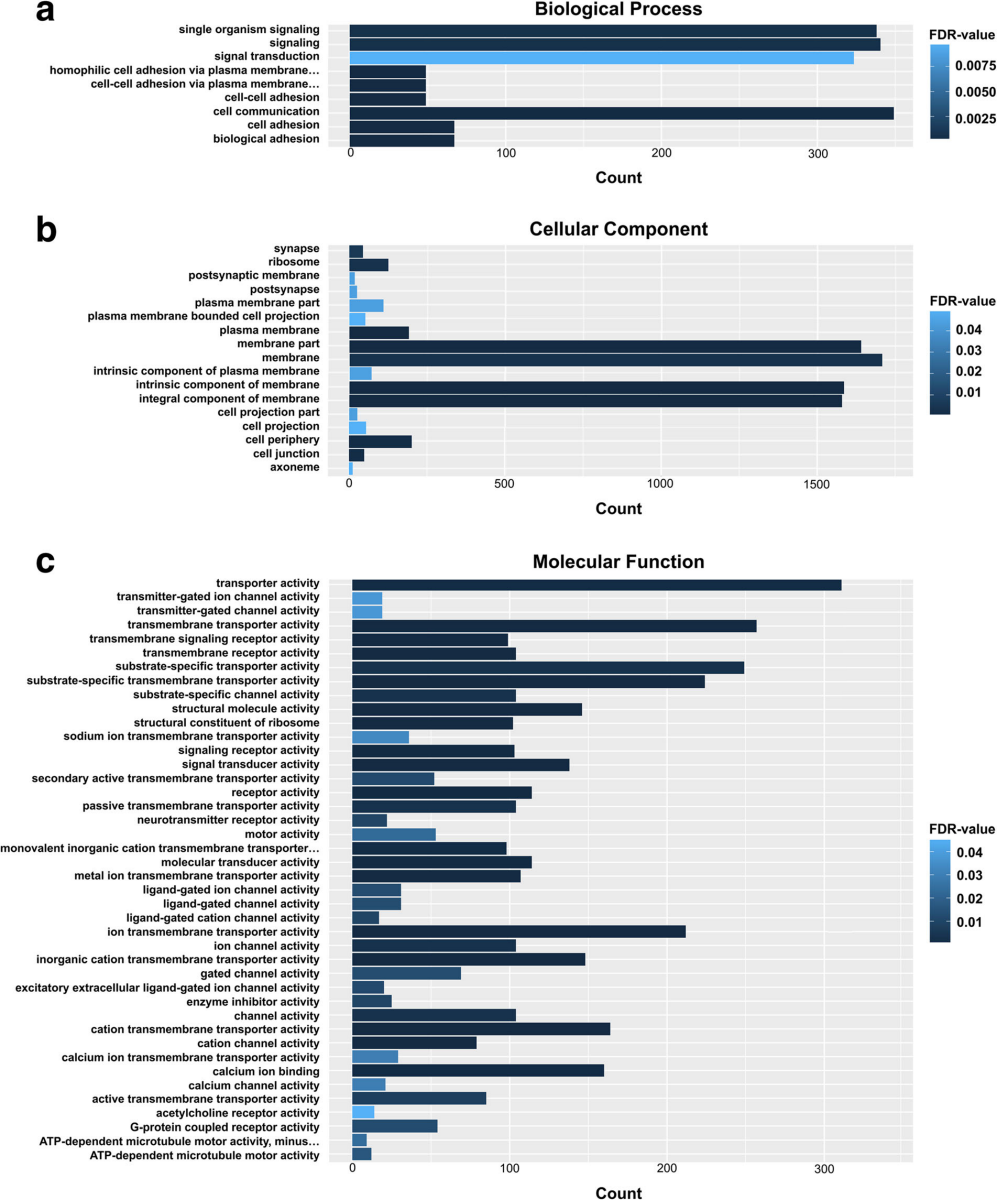
GO analysis of DE transcripts in *S. mekongi* male and female worms. GO categories are organized according to three main ontologies: biological process (**a**), cellular component (**b**) and molecular function (**c**). The x-axis shows the numbers of transcripts. The y-axis shows the GO term. GO terms with higher FDR values are shown in light blue, whereas GO terms with lower FDR values are shown in dark blue

According to the GO classification of DE transcripts, there were 2916, 14,141 and 6693 transcripts assigned to biological process, cellular component and molecular function categories, respectively, of which 1627, 7461 and 3963 transcripts were significantly DE (FDR value < 0.05) (Additional file 4: Table S3). The top three predominant terms for ‘biological process’ were cell communication, signaling, and single organism signaling; for ‘cellular component’ they were membrane, membrane part, and intrinsic component of membrane; and for ‘molecular function’ they were transporter activity, transmembrane transporter activity and substrate-specific transporter activity (Fig. 3a-c).

Furthermore, significant GO terms in each sex was analyzed and demonstrated that the male upregulated transcripts composed of cell communication, single organism signaling and signaling were the top three significant GO terms for ‘biological process’, intrinsic component of membrane, integral component of membrane and plasma membrane were significant GO terms for ‘cellular component’, signal transducer activity, receptor activity and molecular transducer activity were significant GO terms for ‘molecular function’ (Additional file 5: Table S4 and Additional file 6: Figure S2). While, significant GO terms in female upregulated transcripts revealed that cellular amide metabolic process, peptide metabolic process, and amide biosynthetic process were significant GO terms for ‘biological process’, intracellular ribonucleoprotein complex, ribonucleoprotein complex and ribosome were significant GO terms for ‘cellular component’, structural constituent of ribosome, structural molecule activity, and RNA binding were significant GO terms for ‘molecular function’ (Additional file 7: Table S5, Additional file 8: Figure S3).

**Fig. 4:**
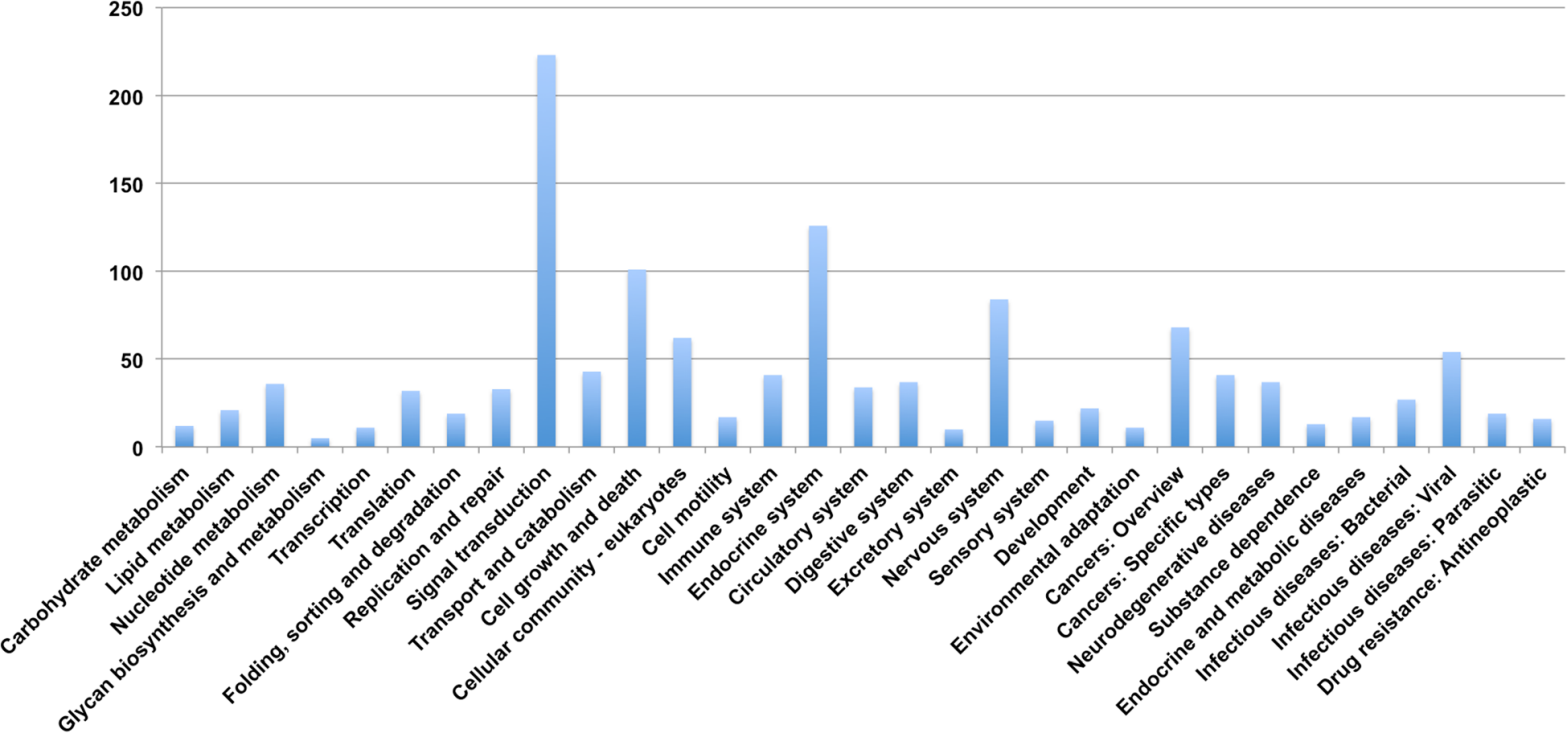
Enrichment pathway analysis of DE transcripts in *S. mekongi* male *vs* female worms (*Q*-value ≤ 0.05). The bar graph shows the number of annotated DE transcripts (y-axis) associated with each sub-class of an enriched pathway (x-axis)

**Fig. 5:**
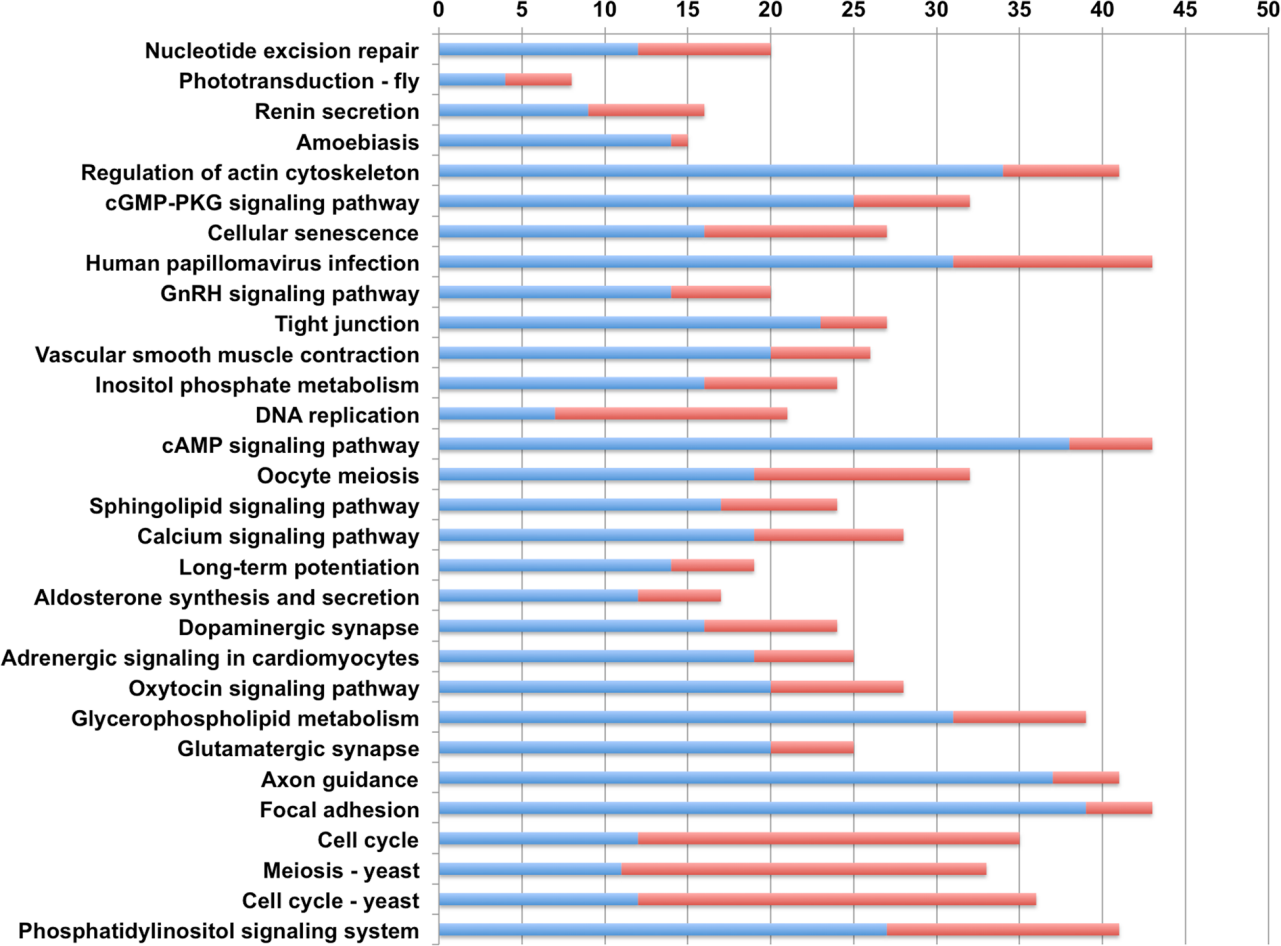
Top 30 most significantly enriched pathways in *S. mekongi* male and female worms. The bar graph shows the number of annotated DE transcripts (x-axis) with each significantly enriched pathway (y-axis). Blue and red colors are the numbers of upregulated transcripts in male and female worms, respectively

**Fig. 6:**
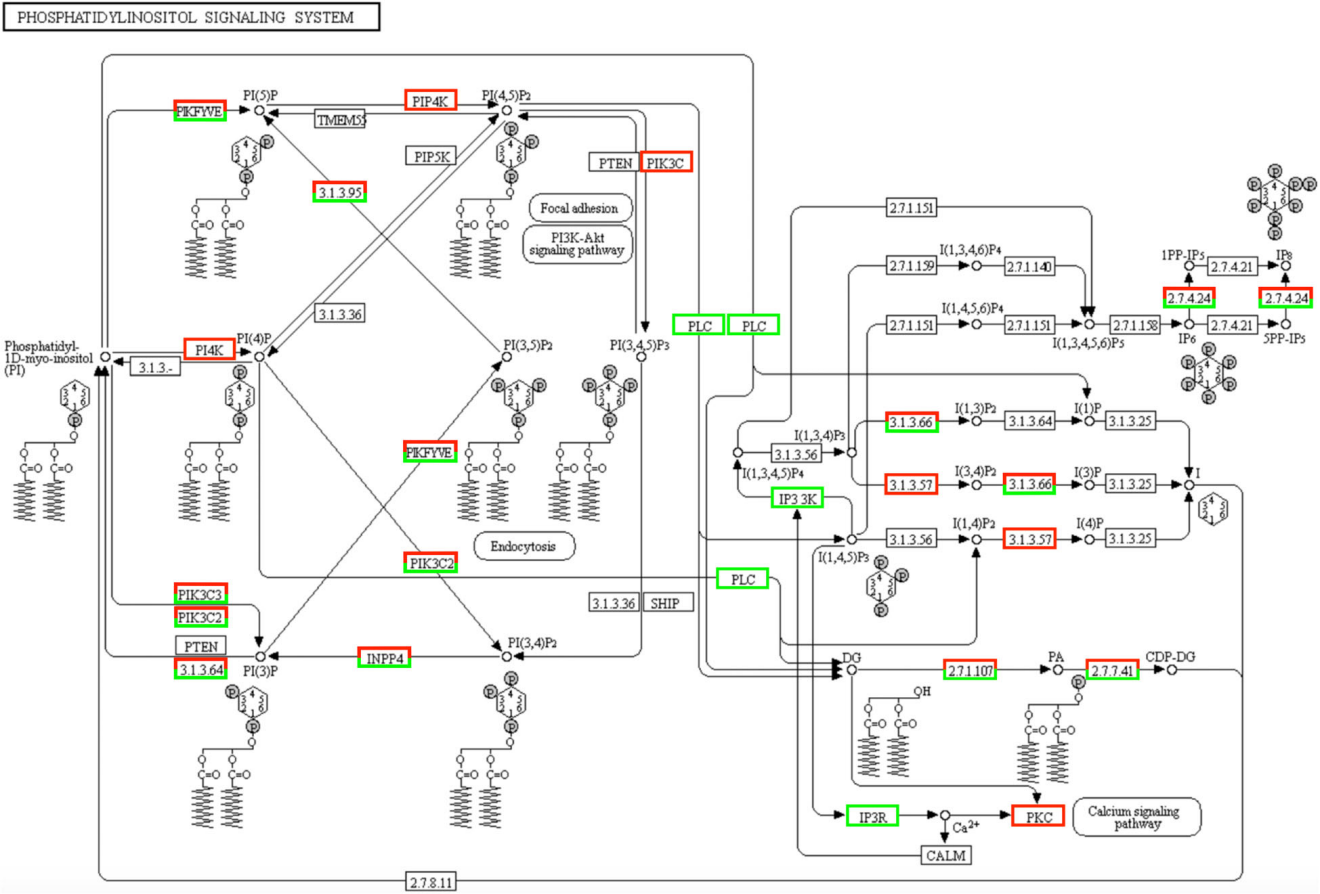
Phosphatidylinositol signaling pathway. Red background indicates upregulated genes in male worms and green background indicates upregulated genes in female worms

KEGG pathway enrichment analysis revealed that a total of 122 pathways were significantly enriched comparing male *vs* female worms (FDR value < 0.05) (Additional file 9: Table S6). These pathways were composed of 397 DE transcripts distributed among the following classes: organismal systems (29.5%), human disease (22.7%), environmental Information processing (17.3%), cellular processes (17.3%), genetic information processing (7.4%) and metabolism (5.7%). The enrichment pathway analysis suggested that the top three sub-classes were composed of transcripts relating to signal transduction (17.3%), endocrine system (9.8%) and cell growth and death (7.8%) (Fig. 4). Analysis of the top 30 most significantly enriched pathways showed that the phosphatidylinositol signaling system was the most significantly enriched pathway (*P* = 4.28E10-09), followed by cell cycle - yeast (*P* = 3.52E-09), meiosis - yeast (*P* = 3.93E-09), cell cycle (*P* = 1.92E-08) and focal adhesion pathways (*P* = 1.02E-07) (Fig. 5). Upregulated transcripts involved in the phosphatidylinositol signaling system are shown in Fig. 6 and Additional file 10: Table S7.

The significant KEGG pathway in each sex demonstrated that the top three significant KEGG pathways in the male worm comprised of focal adhesion, axon guidance and phosphatidylinositol signaling system, respectively. While, cell cycle - yeast, meiosis - yeast and cell cycle were top three significant KEGG pathways in female worms (Additional file 11: Table S8).

### RT-qPCR validation

Thirty representative transcripts (10 upregulated in male worms, 10 upregulated in female worm and 10 non-DE transcripts) were randomly selected for validation of the RNA-Seq analysis using RT-qPCR. The expression of these genes in male and female worms was validated by RT-qPCR, which was highly correlated with the results obtained by RNA-Seq (10 upregulated transcripts in male worms, *r* = 0.793, *P*= 0.006; 10 upregulated transcripts in female worms, *r* = 0.98, *P* < 0.000001; non-DE transcripts, *r* = 0.98, *P* < 0.000001 (Additional file 12: Figure S4).

## Discussion

Mekong schistosomiasis caused by *S. mekongi* remains a health problem in both humans and animals resident in endemic areas [44–46]. Moreover, Mekong schistosomiasis has sporadically emerged throughout the world due to immigration, travel and animal trading [4–7]. To prevent and control this infection, several strategies have been implemented including mass administration of praziquantel in human and reservoir animals, rapid diagnosis to identify asymptomatic cases, snail control and health education. These interventions showed variable results (both successful and unsuccessful) in each area [47]. Thus, novel prevention and control strategies need to be developed based on basic and applied *S. mekongi* biology, especially genomic, transcriptomic and proteomic analyses. However, public databases contain few *S. mekongi* sequences. Herein, we provide the first transcriptomic database of *S. mekongi* adult worms, and compared gene expression between male and female adult parasites.

In this study, RNA-Seq of *S. mekongi* was performed using the Illumina PE sequencing platform with a read length of 150 bases. *De novo* assembly was carried out prior to annotation and downstream analysis. As there was no available reference genome, *de novo* assembly was required to generate contigs. In this regard, the *de novo* transcriptome assembler Bridger was selected as it provides the highest accuracy for paired end reads and the most complete reconstruction of transcripts compared to other assemblers [48].

After annotation, we demonstrated that 4659 and 3510 DE transcripts were significantly upregulated in adult male and female worms, respectively. Genes encoding cytoskeleton-, motor- and neuronal-associated proteins (dynein, tropomyosin 2, myosin light chain kinase and plexin A1) were predominantly upregulated in male worms. Our findings are consistent with previous studies of *S. mansoni* and *S. japonicum* [20, 21, 23, 49–51]. Upregulation of cytoskeleton and motor proteins in male worms may imply their role in the physical support of female worms to facilitate their migration against blood flow from hepatic portal sites to the smaller mesenteric circulation where they lay their eggs [24].

In addition, high expression of adhesion molecule, integrin, was observed in *S. mekongi* male worms. In *S. mansoni*, integrins were predominantly transcribed in reproductive tissue including testes, ovary, vitellarium and ootype. Downexpression of integrins using RNAi affected shape abnormality of oocyte. These indicate an indispensable role of integrin in reproductive processes of *S. mansoni* that may implied to other *Schistosoma* spp. and other Platyhelminthes [52, 53]. CD97 homolog is another adhesion molecule highly upregulated in *S. mekongi* male worms. CD97 is a member of adhesion G protein-coupled receptor expressed on the surface of hematopoietic stem cells, immune cells, smooth muscle, etc. The three known ligands interacting with CD97 have been identified and characterized including complement decay-accelerating factor (CD55), chondroitin sulfate and integrin α5β1 that they are the key molecules playing important roles in several biological and physiological processes in the cells [54]. However, basic function, property and tissue localization of CD97 in parasitic helminths and other pathogens are still unknown and need to be investigated in the future.

In addition to genes associated with cytoskeleton, motor and adhesion, several proteins involved in signaling pathways, including G protein-coupled receptors, tyrosine kinases and serine/threonine kinase pathways were upregulated in male worms. Previously, several studies demonstrated that these signaling pathways were involved in the schistosome life-cycle to control growth, development, sexual differentiation, maturity and even host-parasite interactions. Thus, members of these pathways should be proposed as promising new therapeutic targets against schistosomiasis [55–58].

In the female worm, the antioxidant protein, thioredoxin domain-containing protein 4, was upregulated, which is consistent with the other studies of *S. mansoni* and *S. japonicum* [59–64]. Due to blood degradation in female worms, oxidative stress is generated as a by-product. Thus, higher expression of antioxidant enzymes in female worms has been proposed as a mechanism to protect against damage from oxidative stress. In *S. mansoni*, thioredoxin was not only highly upregulated in female worms but also in eggs. Thus, it has also been proposed that antioxidant mechanisms protect the embryo from oxidative damage resulting from host immunity [59, 61]. The elastase 2b was upregulated and was found among the top 50 most significantly DE transcripts in female worms. RT-qPCR of elastase 2b comparing among male worms, female worms and eggs was performed and the result demonstrated that the gene was expressed the highest in female worms followed by eggs and male worms (Additional file 13: Figure S5). High expression of elastase 2b in mature eggs may facilitate miracedia invade snail intermediate host as same as being required by cercariae to penetrate definitive host skin [65]. However, the real function of elastase 2b in miracedia of schistosomes has not been mentioned elsewhere and need to be investigated in the future. Genes involved in glycosylation, DNA- and protein-misfolding repair and gene regulation were highly enriched in female worms. These may be related to the active cell cycle, proliferation and differentiation during egg production [51].

Among the most significant GO terms in male and female upregulated transcripts, different patterns of gene expression in biological processes were revealed between male and female worms. The result showed that the majority of upregulated genes in male worms were intimately involved in the cell communication, cell signaling and cell adhesion such as delta-like protein, calcium ion binding, putative sodium/calcium exchanger, Rho GTPase-activating protein, G protein-coupled receptor kinase, serine/threonine-protein phosphatase, guanine nucleotide binding protein (G protein), GTP-binding protein, indicating more active host-parasite communication and interaction in male worms. In comparison, these contrasts with the upregulated genes in female worms that played roles in metabolic and biosynthetic processes especially to amide and peptide, indicating that the nutritional acquisition is more crucial for female worms. This is probably reflective of its status of oviposition, which requires abundant nutrition for egg production [51].

The most significant KEGG pathway in male and female upregulated transcripts revealed that structural elements expressed differentially in the male worm included components involved in focal adhesion, regulation of the actin cytoskeleton and tight junctions. In contrast, female worms mostly upregulated genes involved in the cell cycle and meiosis specifically relating to cell division and cell differentiation, such as nipped-B protein, putative cell division cycle 20 (Cdc20) (Fizzy), cell division control protein 45-like protein, cell division cycle 7-related protein kinase, structural maintenance of chromosomes protein, family C50 non-peptidase homologue (C50 family) and the tyrosine-protein kinase Abl. In addition, a set of genes involved in DNA replication processes, DNA polymerase, DNA helicase, putative chromosome transmission fidelity factor and putative DNA primase large subunit are preferentially expressed in the female worm. These results are presumably due to oogenesis and vitellogenesis in female worms [51, 66–68]. Moreover, we found several genes involved in the response to DNA damage or spindle abnormalities assigned to the cell cycle pathway. These included putative rad1 DNA damage checkpoint protein, putative meiotic checkpoint regulator cut4 and putative phosphatidylinositol 3-and 4-kinase, as well as putative DNA polymerase delta small subunit and DNA polymerase III gamma-tau subunit assigned to the genetic information processing pathway (all upregulated in female worms). These observations potentially reflect cell division processes and the need to repair DNA damage caused by oxygen radicals released during the process of hemoglobin digestion, as mentioned in the previous section.

The KEGG pathway analysis revealed pathways associated with sexual differentiation and development, as well as the sex maintenance pathway for female and male worms of *S. mekongi*. These included the GnRH signaling pathway, the insulin signaling pathway and the progesterone-mediated oocyte maturation pathway; these were also identified in previous studies [40, 69, 70]. Our finding that the phosphatidylinositol signaling pathway was most highly enriched was interesting. Inositol hexakisphosphate, calcium-dependent protein kinase C, phosphatidylinositol-4,5-bisphosphate 3-kinase catalytic subunit alpha PI3K, diacylglycerol kinase (DAG kinase), phosphatidylinositol 4-phosphate 3-kinase C2 domain-containing subunit alpha and myotubularin-related protein were upregulated in male worms and inositol 1,4,5-trisphosphate receptor was specifically upregulated in female worms.

Phosphatidylinositol is a phospholipid, which is a crucial component of cellular membranes, and plays important roles in various cellular processes including growth, differentiation and vesicular secretion. The phosphatidylinositol pathway consists of a series of conversions of phosphatidylinositol into singly, doubly and triply phosphorylated forms, and has been implicated in the regulation of numerous cellular functions and responses to extracellular signals [71]. The phosphatidylinositol signaling pathway have been reported to play important roles in a multitude of cellular processes such as membrane trafficking, cell motility, cytoskeletal reorganization, DNA synthesis, the cell cycle, adhesion, signal transduction and reproductive systems [72, 73]. Studies of the role of the phosphatidylinositol pathway in *C. elegans* found that its phosphorylated forms have multiple functions; for example, phosphatidylinositol-3-OH kinase, diacylglycerol kinase, phosphatidylinositol 4-phosphate 5-kinase and phosphatidylinositol 3-kinase, play important roles in longevity and diapause [74], thermotactic behavior [75], ovulation and fertilization [76] and the behavioral learning response [77], respectively.

In mammals, the phosphatidylinositol 3-kinase (PI3K) signal transduction pathway is necessary for seminiferous cord formation, testis and vascular development during testis morphogenesis. Inhibition of PI3K using the specific inhibitor, LY294002, blocked cord formation and impacted the number of cords and their vascular density [78, 79]. The phosphatidylinositol signaling pathway may be indispensable for *S. mekongi* survival, especially development of the male reproductive system. In the future, the functions and properties of the phosphatidylinositol signaling pathway in sex development of schistosomes need to be elucidated prior to consideration of pathway members as novel therapeutic targets against schistosomiasis.

## Conclusions

In this study, we presented a transcriptomic analysis of male and female *S. mekongi* adult worms using RNA-Seq data. The study revealed the key biological and physiological features of male and female *S. mekongi* worms. These data will serve as a sequence resource for future studies of gene functions and will aid the ongoing whole genome sequencing efforts for *S. mekongi*, which may further facilitate the discovery of novel interventions against this persistent parasite.

## Methods

### Parasite materials

Adult *S. mekongi* worms used in this study were maintained by alternating infection of the intermediate snail host, *N. aperta*, and mice (*Mus musculus*) according to a standard protocol [80]. The *N. aperta* snails were collected from the Mekong River, Ubon Ratchathani Province, Thailand. Eight-week-old female ICR mice were purchased from the National Laboratory Animal Center, Mahidol University. Mice were infected with 25–30 cercariae by percutaneous exposure at the abdomen and then housed in the Animal Care Unit, Faculty of Tropical Medicine, Mahidol University. Eight weeks post-infection, mature adult worms were recovered from the infected mice by hepatic perfusion with 0.85% normal saline solution. Adult males and females were separated manually under a dissecting microscope. The worms were carefully washed in normal saline solution and immediately frozen at -80 °C until the time of RNA extraction.

### RNA extraction, cDNA library construction, RNA sequencing and quality control

Total RNA was extracted from pooled adult male (*n* = 20) and female (*n* = 40) worms. Extractions for each sex were performed with three biological replicates using TRIZOL^TM^ reagent (Invitrogen, Carlsbad, CA, USA) according to the manufacturer’s instructions. RNA quantity and quality were assessed using a NanoDrop® ND-1000 spectrophotometer (Thermo Fisher Scientific Inc., Wilington, DE, USA) and a 2100 Bioanalyzer (Agilent Technologies, Palo Alto, CA, USA).

For each replicate, cDNA libraries were constructed from 1 μg of total RNA (Additional file 14: Figure S6) using the NEBNext® Ultra^TM^ RNA Library Prep Kit for Illumina® according to the manufacturer’s protocol (New England Biolabs (NEB), Ipswich, MA, USA). Briefly, mRNA isolation was performed using oligo(dT) magnetic beads contained in the NEBNext® Poly(A) mRNA Magnetic Isolation Module (NEB). The mRNAs were fragmented and reverse transcribed using NEBNext® First Strand Synthesis Reaction Buffer and NEBNext® Random Primers. First-strand cDNA was synthesized using ProtoScript II Reverse Transcriptase and second-strand cDNA was synthesized using Second Strand Synthesis Enzyme Mix. Double-stranded cDNA was purified using the AxyPrep™ Mag PCR Clean-up kit (Axygen Biosciences, Union City, CA, USA) and treated with End Prep Enzyme Mix to repair both ends, add a dA-tail, and add adaptors to both ends *via* TA ligation. Size selection of adaptor-ligated DNA was performed using the AxyPrep™ Mag PCR Clean-up kit, and fragments of ~360 bp were recovered. Each sample was then amplified by PCR for 11 cycles using P5 and P7 primers; both of these primers carried sequences that can anneal to the Illumina flow cell and perform bridge PCR, and the P7 primer carried a six-base index sequence allowing for multiplexing. The PCR products were purified using the AxyPrep™ Mag PCR Clean-up kit, analyzed using an Agilent 2100 Bioanalyzer and quantified using a Qubit 2.0 Fluorometer (Invitrogen). Barcoded libraries with different index sequences were multiplexed and loaded on an Illumina HiSeq X instrument according to the manufacturer’s instructions (Illumina, San Diego, CA, USA). Sequencing was carried out using a 2 × 150 bp paired-end (PE) configuration; image analysis and base calling were conducted using the HiSeq Control Software (HCS) + RTA 2.7 (Illumina) on the HiSeq X instrument.

In order to assess sequencing quality, the raw sequencing reads were processed using the following steps. First, raw sequencing reads was assessed with FastQC software (v0.11.2) (http://www.bioinformatics.babraham.ac.uk/projects/fastqc/) to estimate read quality using PHRED quality scores. Secondly, adaptor sequences and low-quality bases (below Q30) were trimmed from the raw reads with Trimmomatic software (v.0.32) [81]. Finally, any resulting reads that were at least 50 bases long were selected for further analysis.

### Assembly of transcripts

*De novo* assembly of clean reads was performed using the Bridger assembler [82] with either 25 or 31 k-mer sizes. The quality of each assembly was assessed using Transrate (v.1.0.3) [83] and the sequence read realignment rates for each assembly’s contigs were computed with HISAT2 (v.2.0.5) [84]. In this study, the contig sequences from the assembly with k-mer size 31 were selected for analysis due to this assembly’s high realignment rate and higher N50 value. Contig sequences with coverage > 10 reads were extracted and clustered with the CD-HIT-EST algorithm [85] to filter redundant sequences. The longest sequences from each cluster were selected as the final assembly.

### Annotation of transcripts

Protein-coding sequence (CDS) regions and open reading frames (ORFs) were predicted for each assembled transcript using the TransDecoder v.5.0.1 algorithm (http://transdecoder.github.io). The function of the predicted protein sequences was annotated using BLAST v.2.2.31 (BLASTP algorithm) [86], with searches against the UniProt Trematoda database (https://www.ncbi.nlm.nih.gov/pmc/articles/PMC2238893/) [87]. Transcripts for which no CDS regions were predicted were subsequently analyzed with BLAST v.2.2.31 (BLASTX algorithm) [86] for alternative functional assignment. The protein family of annotated proteins was analyzed through a protein profile database (Pfam) search using HMMER3 software [88]. A threshold of *E*-value < 10^-5^ was applied in all the functional prediction analyses.

### Analysis of differentially expressed (DE) transcripts

Trimmed sequence reads were aligned back to the assembled transcriptome using Bowtie2 (v.2.2.9) [89]. Discordant alignments were excluded. TPM (transcripts per million), FPKM (Fragments Per Kilobase Of Exon Per Million Fragments Mapped), credibility intervals and expected counts were computed using RSEM (v.1.3.0) [90]. Transcripts with expression values of more than 1 count per-million (cpm) were extracted for differential expression analysis. Analysis of DE transcripts comparing male *vs* female worms was conducted using edgeR v.3.20.7 [91]. The expression fold change between sexes were calculated and log-transformed. Significantly DE transcripts between sexes were extracted according to the following criteria: false discovery rate (FDR) < 0.05 and fold change > 2.0.

### Gene ontology (GO) and KEGG pathway analyses

Significantly DE transcripts were annotated with GO terms for assignment to three major GO categories: cellular components, biological processes and molecular functions. The GO annotation was performed with the R package topGO (v.2.28.0) (https://bioconductor.org/packages/release/bioc/html/topGO.html). Enrichment was assessed with classic Fisher’s exact tests in topGO and multiple testing was done with FDR. Significant GO terms with FDR values < 0.05 were extracted. Significantly DE transcripts were annotated with pathway information from the KEGG pathway database using the R package clusterProfiler [92]. Pathway over-representation was assessed with a significance cut-off of 0.05.

### Quantitative real-time polymerase chain reaction (RT-qPCR)

A total of 10 male-associated and 10 female-associated DE transcripts, as well as 10 non-DE transcripts, were randomly selected for validation using RT-qPCR. First-strand cDNA synthesis and qPCR validation experiments were performed with three different biological replicates from the same samples used in RNA-Seq experiments. Total RNA (1 μg) from each biological replicate was treated with 1 U of DNase I (Thermo Fisher Scientific, Waltham, MA, USA) and used to synthesize first-strand cDNA using a RevertAid First Strand cDNA Synthesis kit (Thermo Fisher Scientific) according to the manufacturer’s instructions. The expression level of each gene was determined by SYBR Green RT-qPCR. Each 20 μl reaction mixture contained 2 μl of first-strand cDNA, 1× iTaq Universal SYBR Green Supermix (Bio-Rad Laboratories, Philadelphia, PA, USA), and 300 nM each of forward and reverse primers. To normalize gene expression levels, 18S ribosomal RNA (rRNA) was used as an internal control [93, 94]. Amplification was performed on a CFX96 Real-Time PCR System (Bio-Rad Laboratories, Hercules, CA, USA), according to the following protocol: pre-incubation at 95 °C for 5 min, followed by 40 cycles of 95 °C for 20 s and 60 °C for 1 min. A melting curve analysis was performed from 65–95 °C. Gene expression levels were calculated using the 2^-∆∆*C*t^ method [95]. RT-qPCR experiments were performed with three replicates. Primer sequences for each target gene were designed using Primer3Plus (http://www.bioinformatics.nl/cgi-bin/primer3plus/primer3plus.cgi) with default parameters and are listed in Additional file 15: Table S9. Correlation between RNA-Seq and qPCR results for 30 representative transcripts was analyzed using the Spearman’s rank test.

## Additional files

Additional file 1: **Figure S1.** Base quality distribution of raw reads and trimmed paired-end reads. (PDF 2840 kb)

Additional file 2: **Table S1.** Summary of raw sequencing reads, trimmed sequencing reads and sequence read assemblies. (XLSX 43 kb)

Additional file 3: **Table S2.** Detailed information on the top 50 most significantly upregulated DE transcripts. (XLSX 81 kb)

Additional file 4: **Table S3.** Detailed information on GO term analysis of DE transcripts. (XLSX 78 kb)

Additional file 5: **Table S4.** Detailed information on GO term analysis in male upregulated transcripts. (XLSX 99 kb)

Additional file 6: **Figure S2.** Bar graph for GO term analysis in male upregulated transcripts. (PDF 1856 kb)

Additional file 7: **Table S5.** Detailed information on GO term analysis in female upregulated transcripts. (XLSX 94 kb)

Additional file 8: **Figure S3.** Bar graph for GO term analysis in female upregulated transcripts. (PDF 1341 kb)

Additional file 9: **Table S6.** Detailed information on pathway analysis of DE transcripts. (XLSX 68 kb)

Additional file 10: **Table S7.** Detailed information on function of upregulated genes belonging to the phosphatidylinositol signaling pathway. (XLSX 70 kb)

Additional file 11: **Table S8.** Detailed information on pathway analysis in male and female upregulated transcripts. (XLSX 72 kb)

Additional file 12: **Figure S4.** Correlation between RNA-Seq and RT-qPCR results. (PDF 90 kb)

Additional file 13: **Figure S5.** Relative expression level of elastase 2b in male worms, female worms and eggs. (PDF 162 kb)

Additional file 14: **Figure S6.** RNA integrity analysis. (PDF 1602 kb)

Additional file 15: **Table S9.** Oligonucleotide primer sets used for RT-qPCR validation. (PDF 58 kb)

## Abbreviations

CDS: Coding DNA sequence
CPM: Counts per million
DE: Differentially expressed
ESTs: Expressed sequence tags
FC: Fold change
FDR: False discovery rate
FPKM: Fragments per kilobase of exon per million fragments mapped
GO: Gene ontology
KEGG: Kyoto Encyclopedia of Genes and Genomes
NGS: Next-generation sequencing
ORFs: Open reading frames
PF: Paired-end
Pfam: Protein profile database
SRA: Sequence read archive
TPM: Transcripts per million
UniProt: The Universal Protein Knowledgebase
