# Supplementary material for: Transcriptomic analysis of male and female Schistosoma mekongi adult worms
Source: Parasit Vectors. 2018 Sep 10;11:504. doi: 10.1186/s13071-018-3086-z (PMC6131826; doi:10.1186/s13071-018-3086-z)

Additional file 1: Figure S1

Base quality distribution of raw reads and trimmed paired-end reads

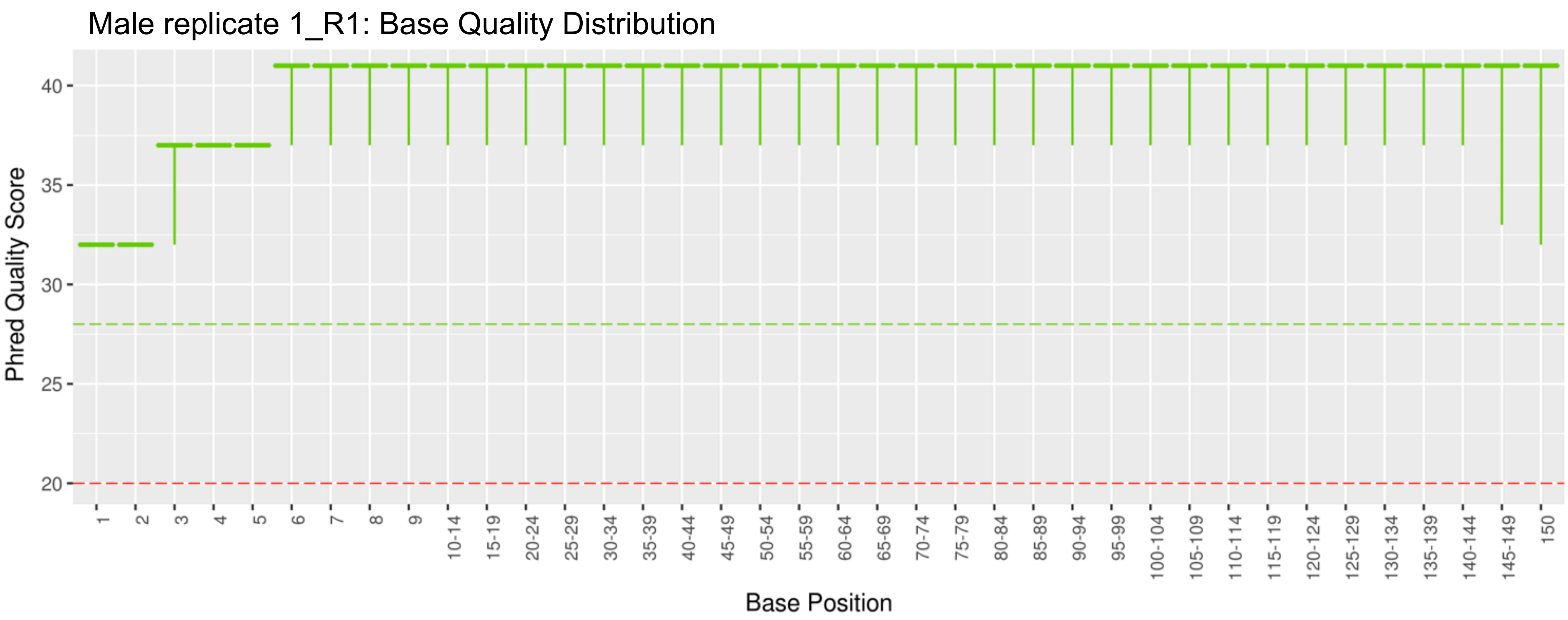

Male replicate 1_R1: Base Quality Distribution
Phred Quality Score
40
35
30
25
20
1
2
3
4
5
6
7
8
9
10-14
15-19
20-24
25-29
30-34
35-39
40-44
45-49
50-54
55-59
60-64
65-69
70-74
75-79
80-84
85-89
90-94
95-99
100-104
105-109
110-114
115-119
120-124
125-129
130-134
135-139
140-144
145-149
150
Base Position

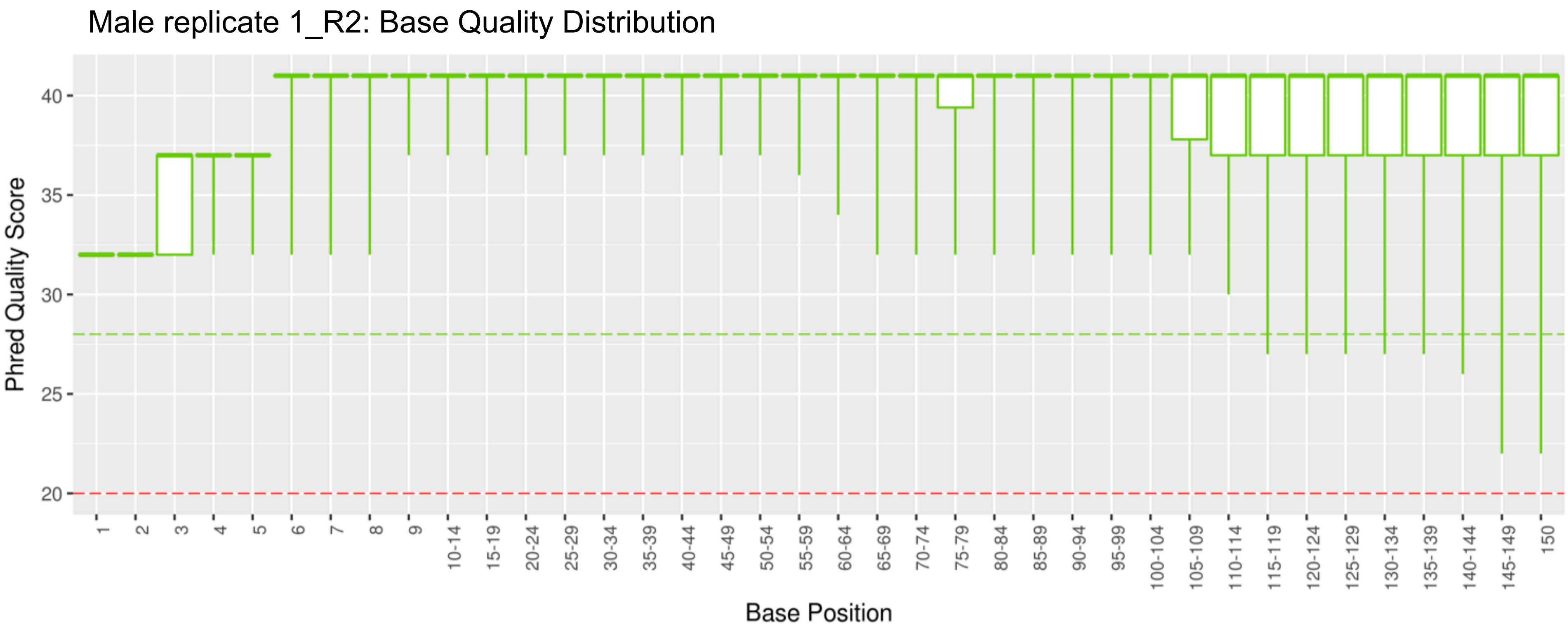

Male replicate 1_R2: Base Quality Distribution
Phred Quality Score
40
35
30
25
20
1
2
3
4
5
6
7
8
9
10-14
15-19
20-24
25-29
30-34
35-39
40-44
45-49
50-54
55-59
60-64
65-69
70-74
75-79
80-84
85-89
90-94
95-99
100-104
105-109
110-114
115-119
120-124
125-129
130-134
135-139
140-144
145-149
150
Base Position

Male replicate 2_R1: Base Quality Distribution

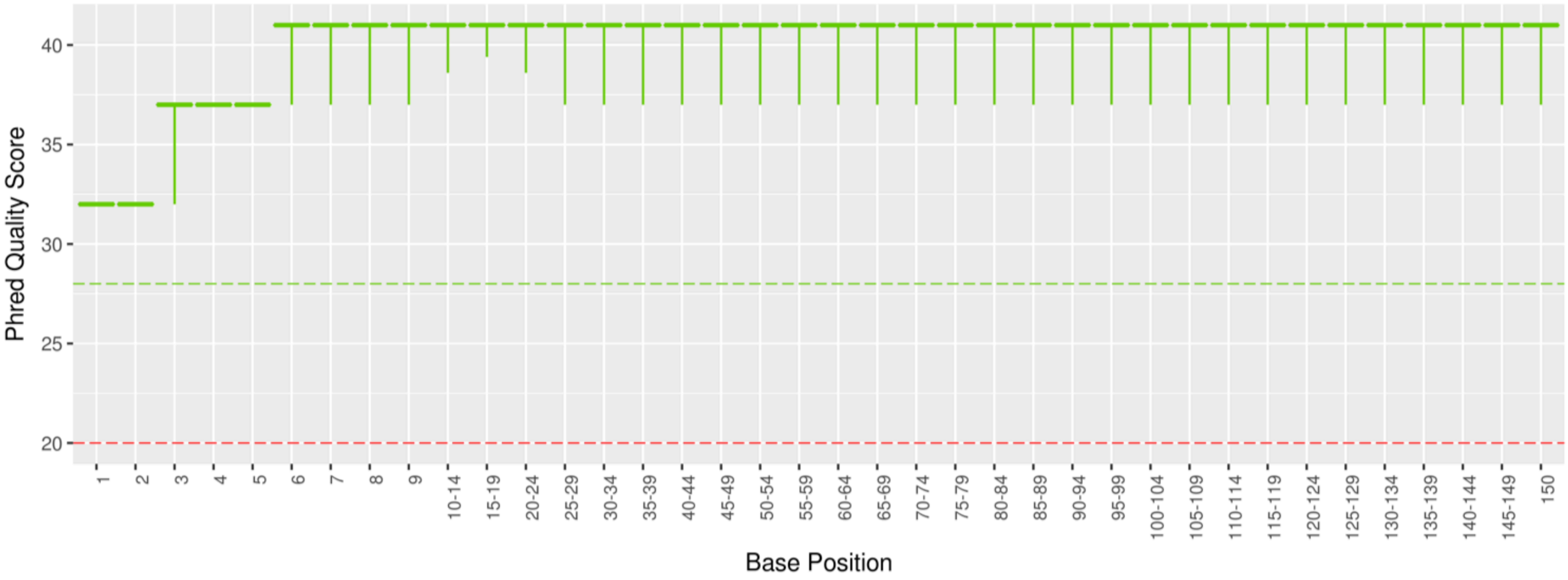

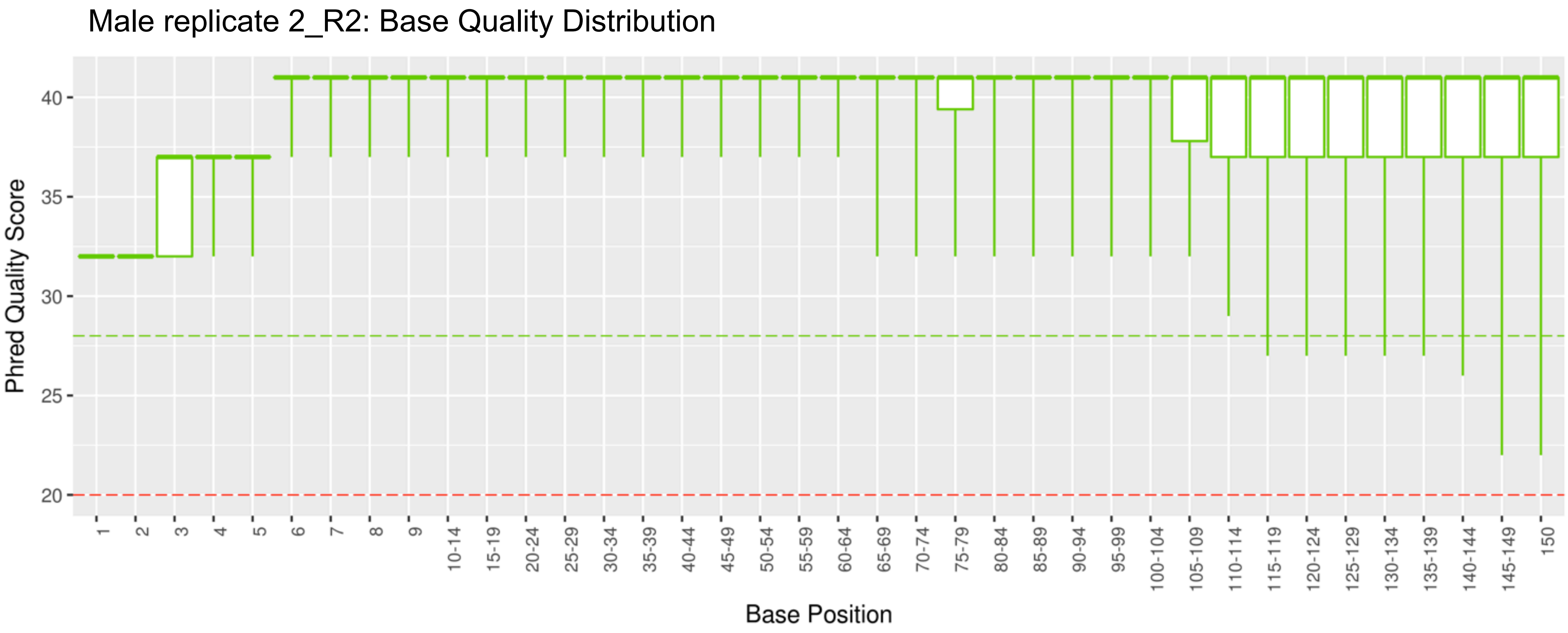
Male replicate 2_R2: Base Quality Distribution
Phred Quality Score
40
35
30
25
20
1
2
3
4
5
6
7
8
9
10-14
15-19
20-24
25-29
30-34
35-39
40-44
45-49
50-54
55-59
60-64
65-69
70-74
75-79
80-84
85-89
90-94
95-99
100-104
105-109
110-114
115-119
120-124
125-129
130-134
135-139
140-144
145-149
150
Base Position

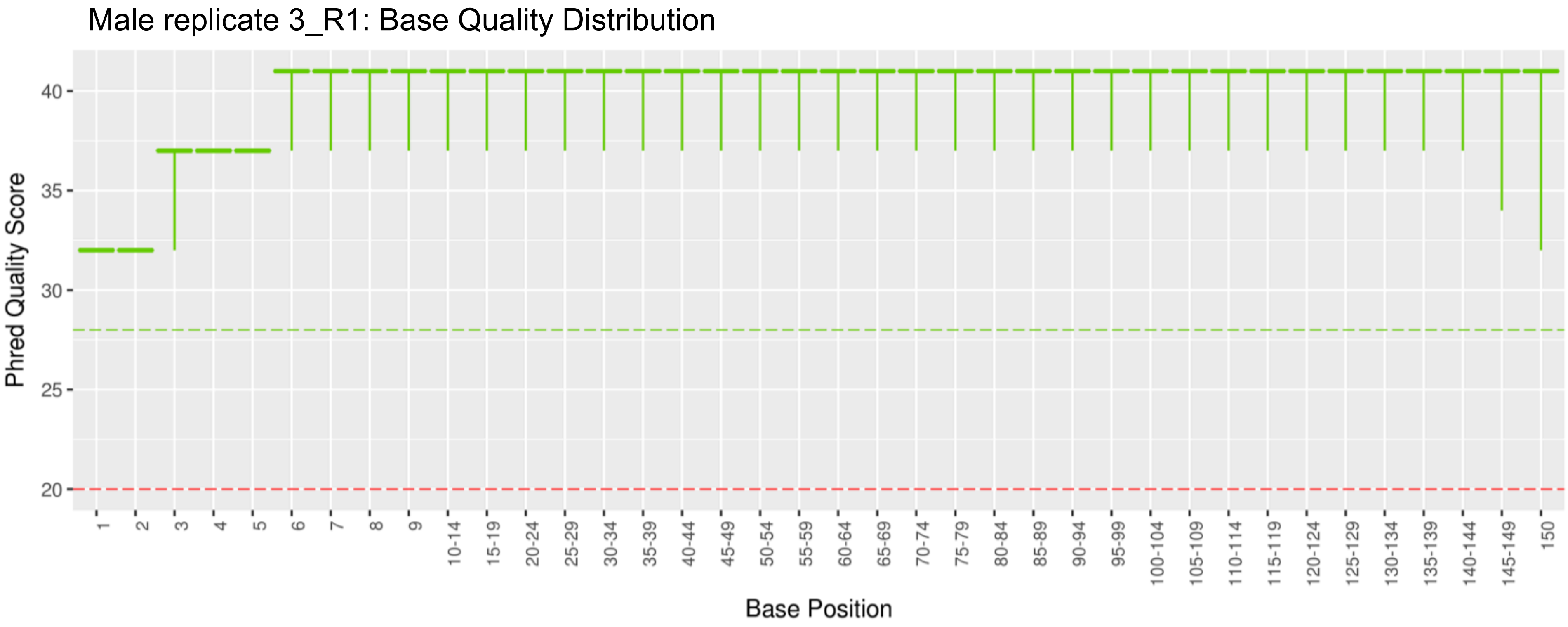

Male replicate 3_R1: Base Quality Distribution
Phred Quality Score
40
35
30
25
20
1
2
3
4
5
6
7
8
9
10-14
15-19
20-24
25-29
30-34
35-39
40-44
45-49
50-54
55-59
60-64
65-69
70-74
75-79
80-84
85-89
90-94
95-99
100-104
105-109
110-114
115-119
120-124
125-129
130-134
135-139
140-144
145-149
150
Base Position

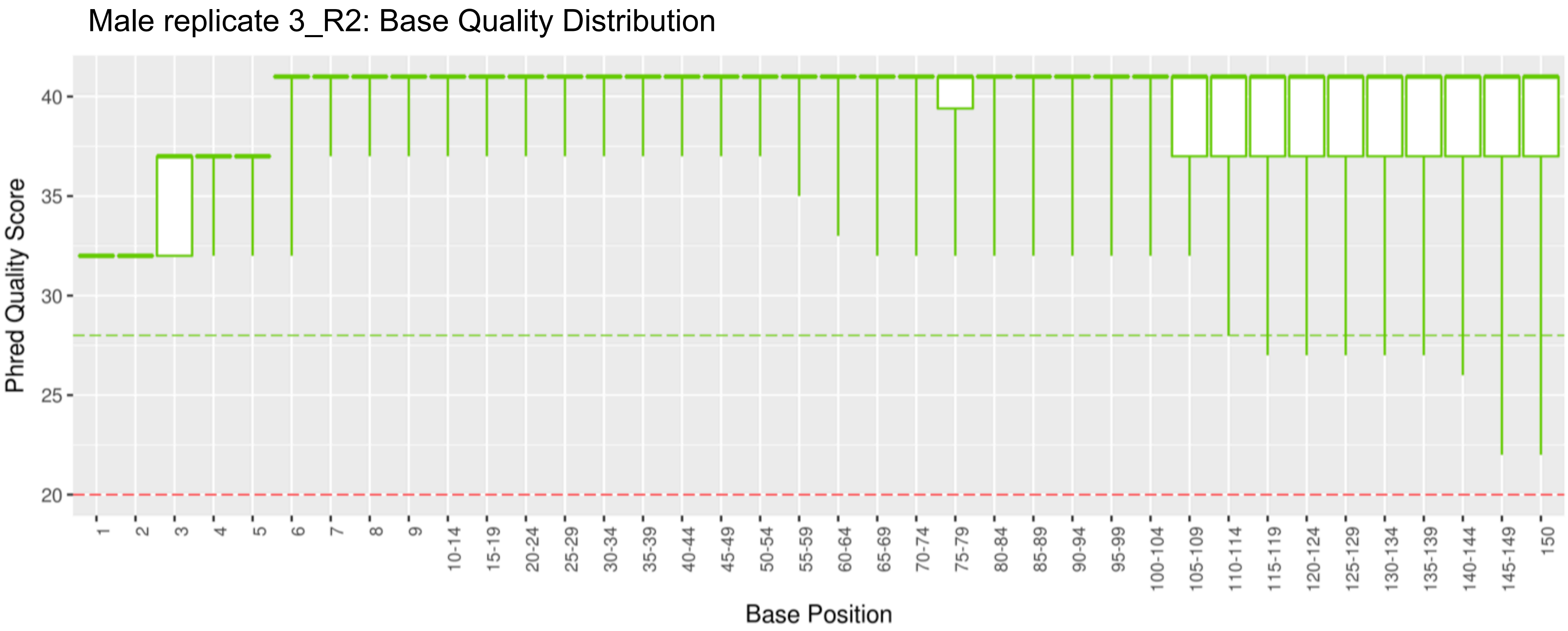
Male replicate 3_R2: Base Quality Distribution
Phred Quality Score
40
35
30
25
20
1
2
3
4
5
6
7
8
9
10-14
15-19
20-24
25-29
30-34
35-39
40-44
45-49
50-54
55-59
60-64
65-69
70-74
75-79
80-84
85-89
90-94
95-99
100-104
105-109
110-114
115-119
120-124
125-129
130-134
135-139
140-144
145-149
150
Base Position

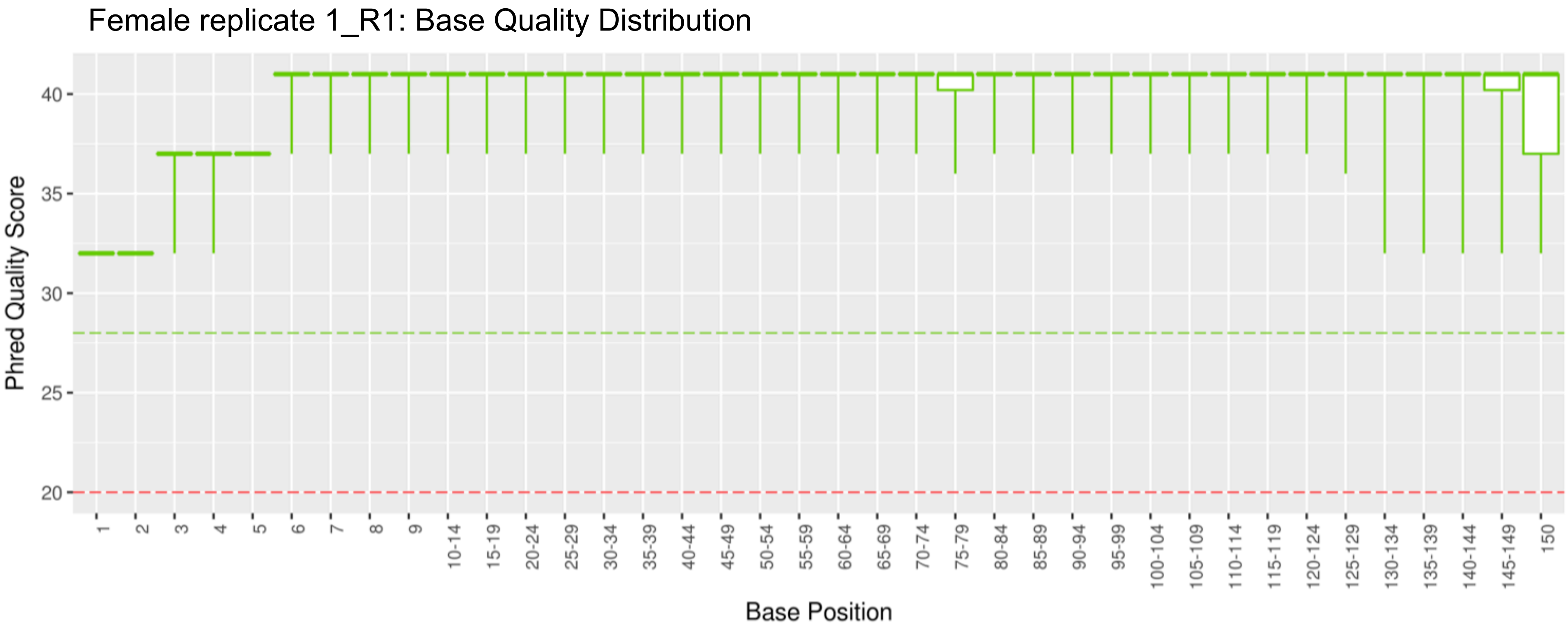
Female replicate 1_R1: Base Quality Distribution
Phred Quality Score
40
35
30
25
20
1
2
3
4
5
6
7
8
9
10-14
15-19
20-24
25-29
30-34
35-39
40-44
45-49
50-54
55-59
60-64
65-69
70-74
75-79
80-84
85-89
90-94
95-99
100-104
105-109
110-114
115-119
120-124
125-129
130-134
135-139
140-144
145-149
150
Base Position

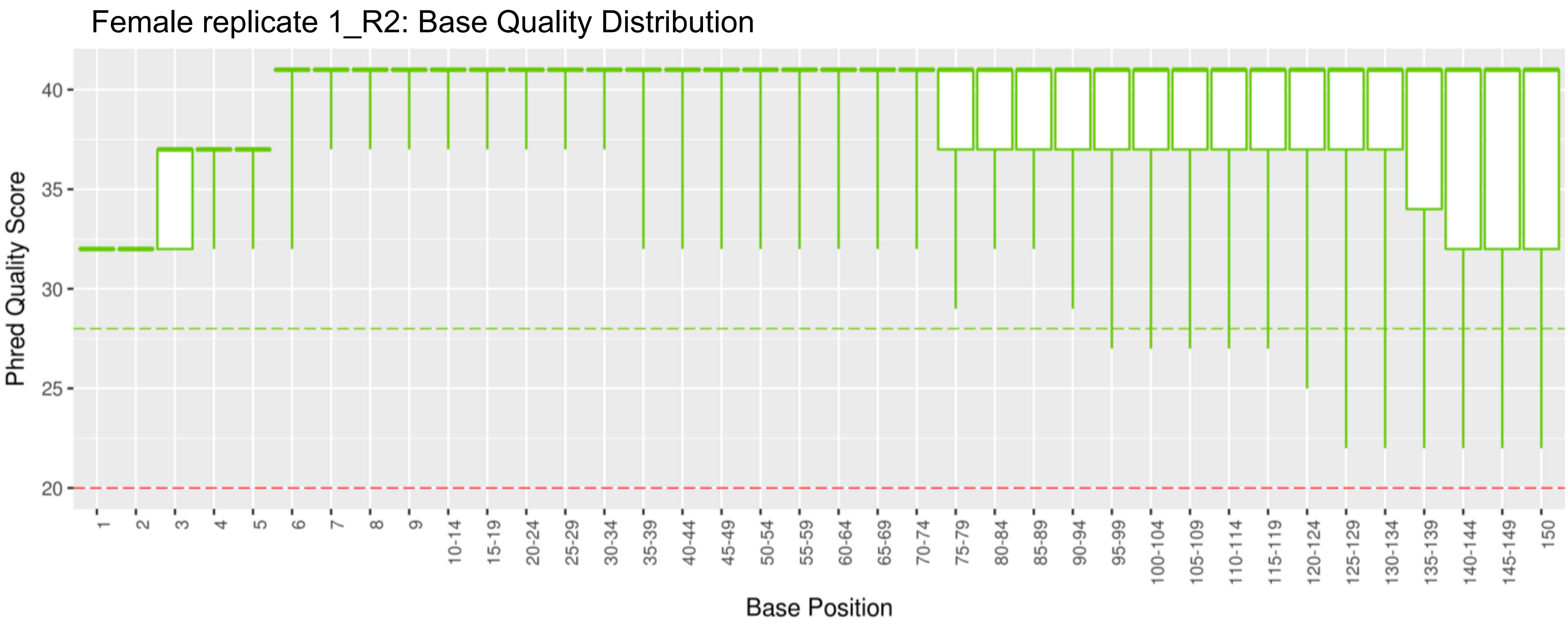
Female replicate 1_R2: Base Quality Distribution
Phred Quality Score
40
35
30
25
20
1
2
3
4
5
6
7
8
9
10-14
15-19
20-24
25-29
30-34
35-39
40-44
45-49
50-54
55-59
60-64
65-69
70-74
75-79
80-84
85-89
90-94
95-99
100-104
105-109
110-114
115-119
120-124
125-129
130-134
135-139
140-144
145-149
150
Base Position

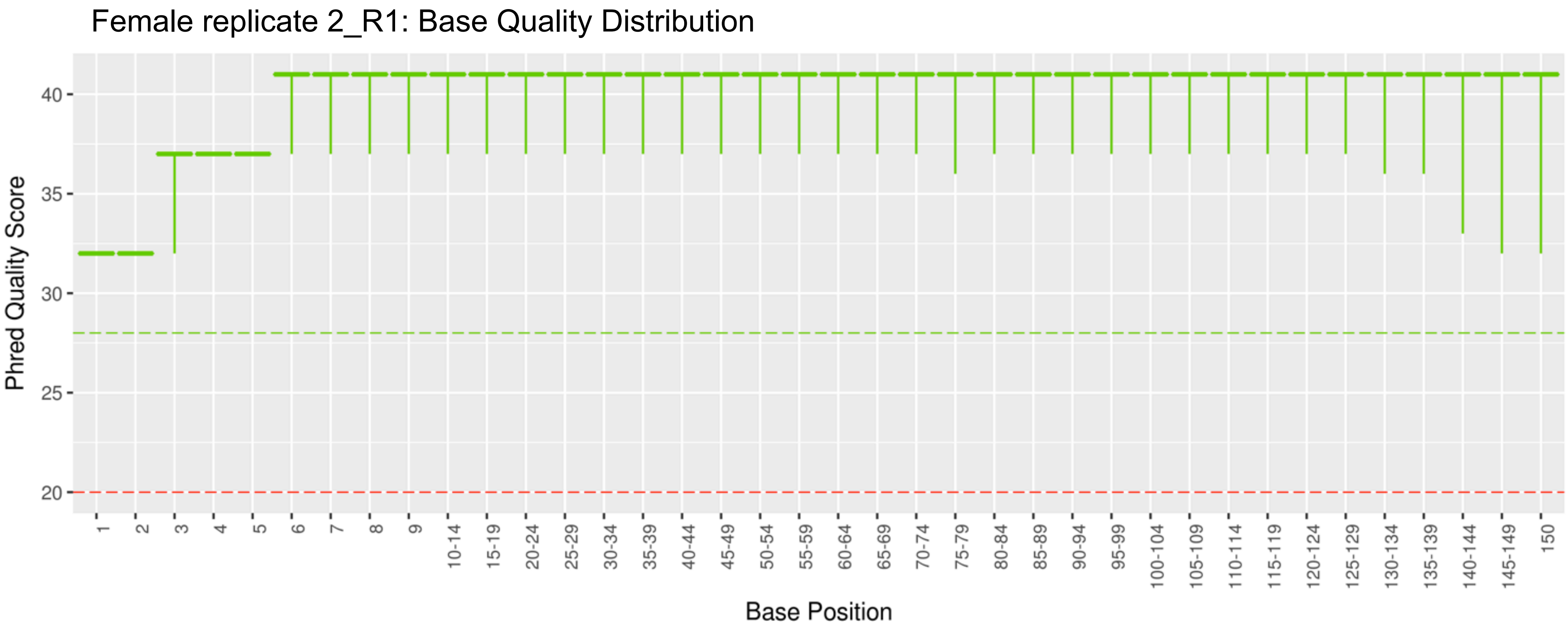
Female replicate 2_R1: Base Quality Distribution
Phred Quality Score
40
35
30
25
20
1
2
3
4
5
6
7
8
9
10-14
15-19
20-24
25-29
30-34
35-39
40-44
45-49
50-54
55-59
60-64
65-69
70-74
75-79
80-84
85-89
90-94
95-99
100-104
105-109
110-114
115-119
120-124
125-129
130-134
135-139
140-144
145-149
150
Base Position

Female replicate 2_R2: Base Quality Distribution

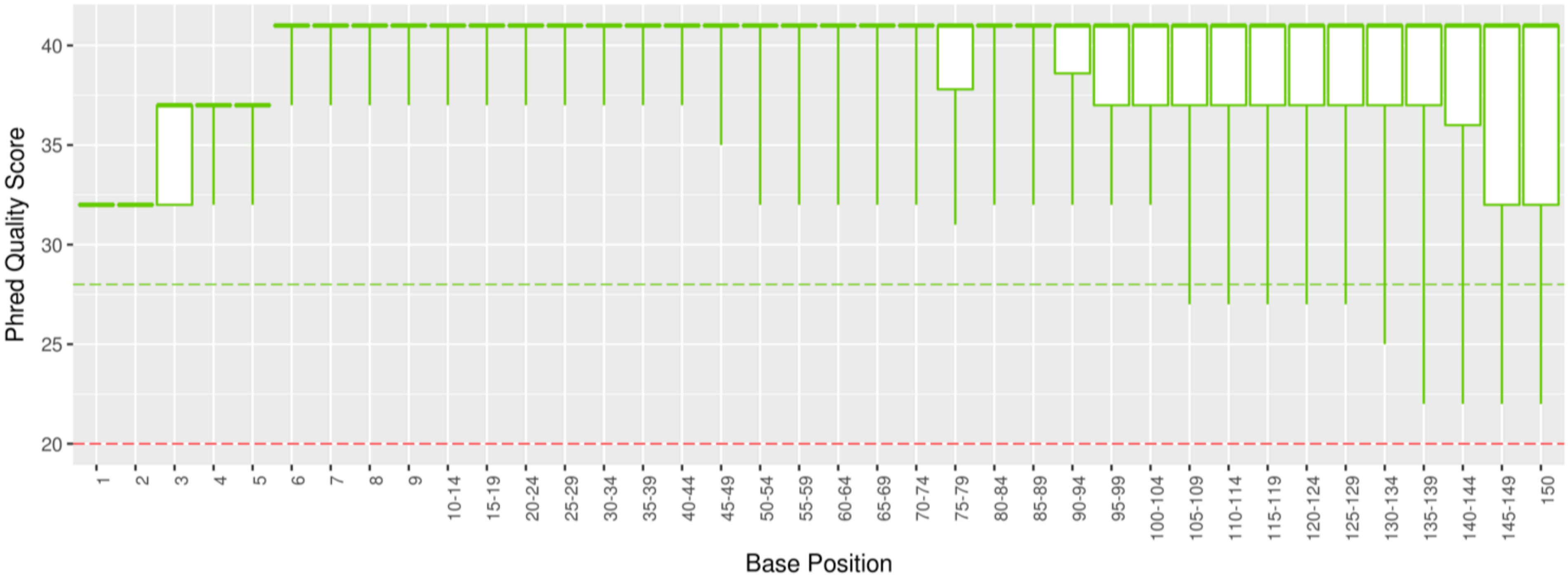

Female replicate 3_R1: Base Quality Distribution

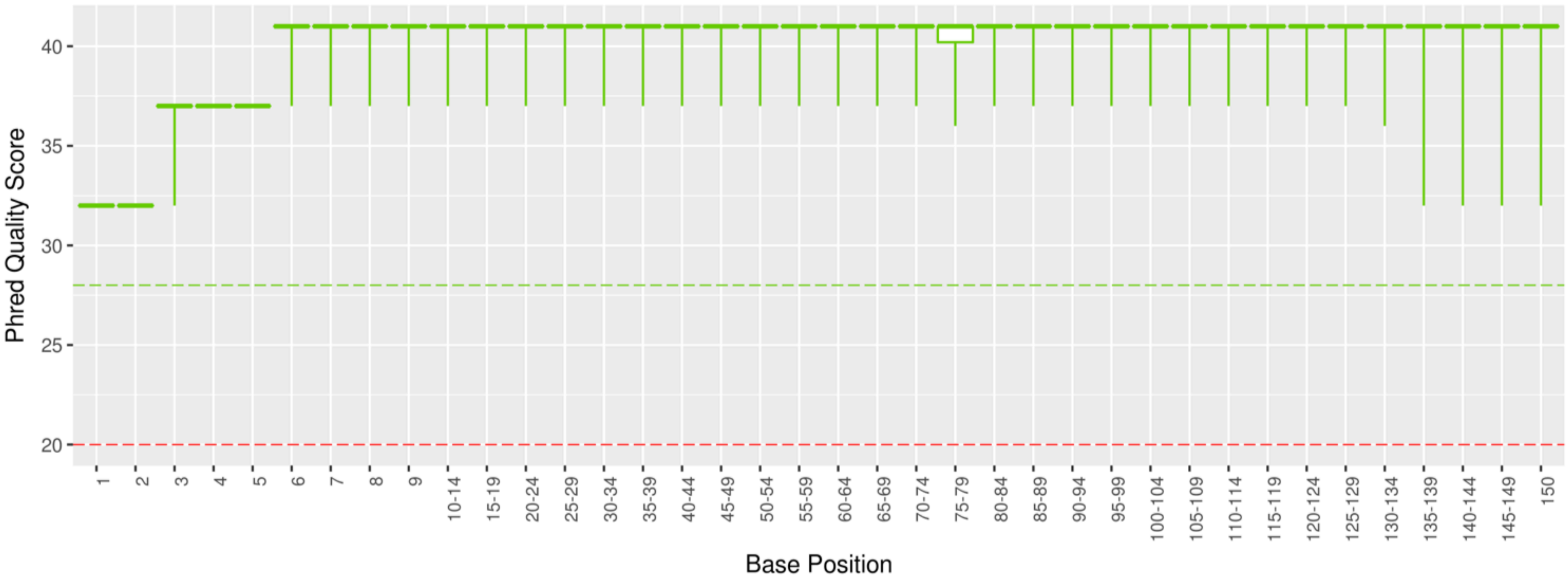

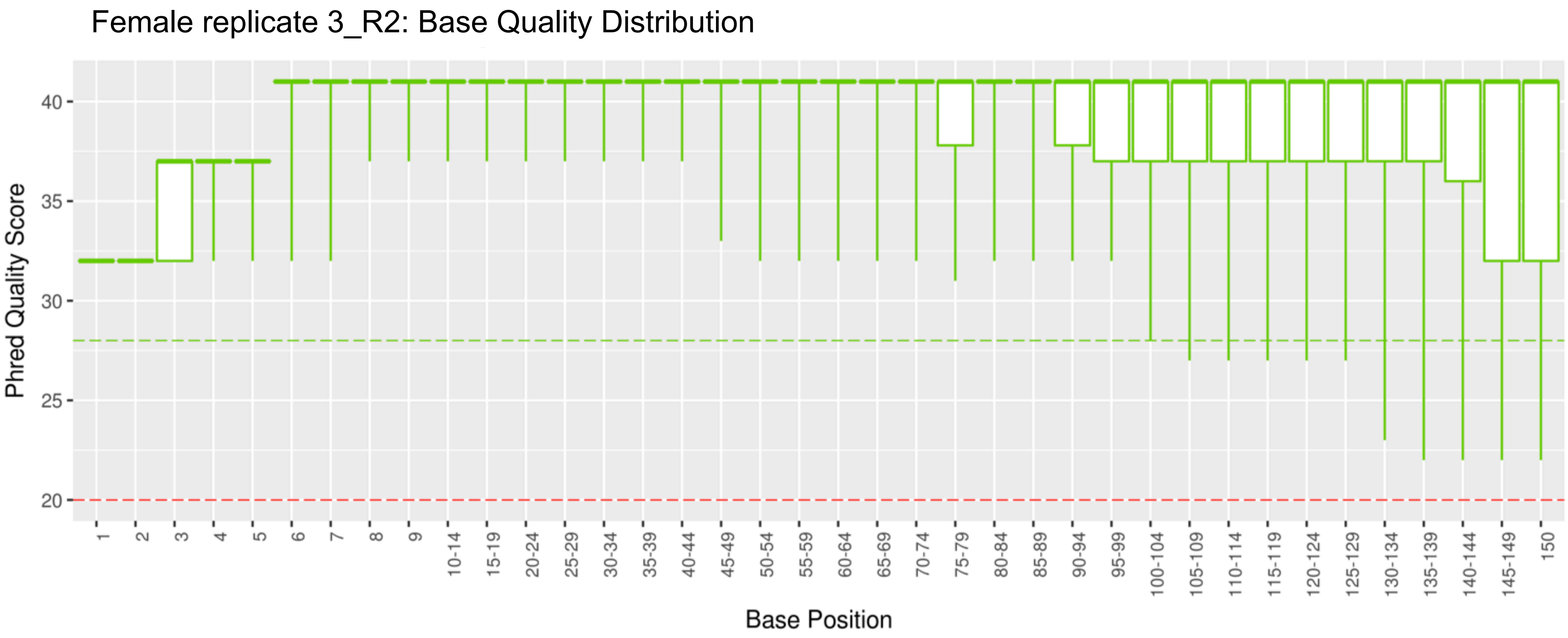
Female replicate 3_R2: Base Quality Distribution
Phred Quality Score
40
35
30
25
20
1
2
3
4
5
6
7
8
9
10-14
15-19
20-24
25-29
30-34
35-39
40-44
45-49
50-54
55-59
60-64
65-69
70-74
75-79
80-84
85-89
90-94
95-99
100-104
105-109
110-114
115-119
120-124
125-129
130-134
135-139
140-144
145-149
150
Base Position

Trimmed Read Quality

Male replicate 1_R1-paired: Base Quality Distribution

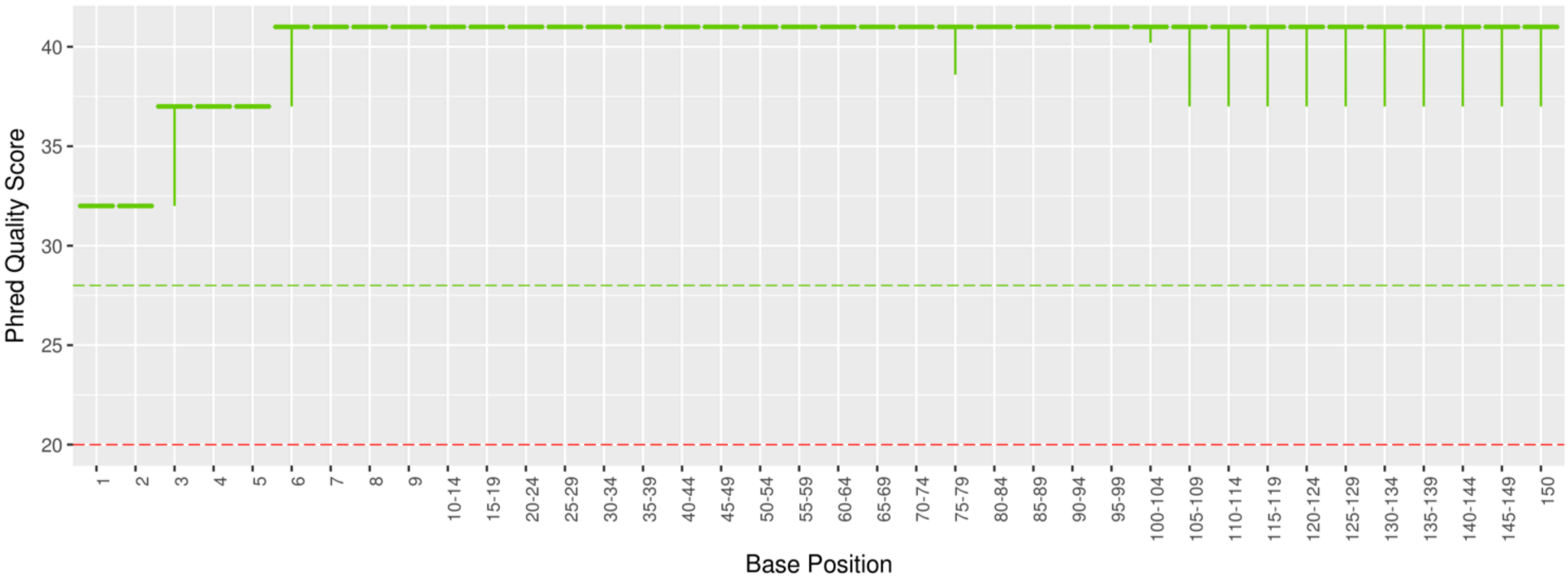

Male replicate 1_R2-paired: Base Quality Distribution

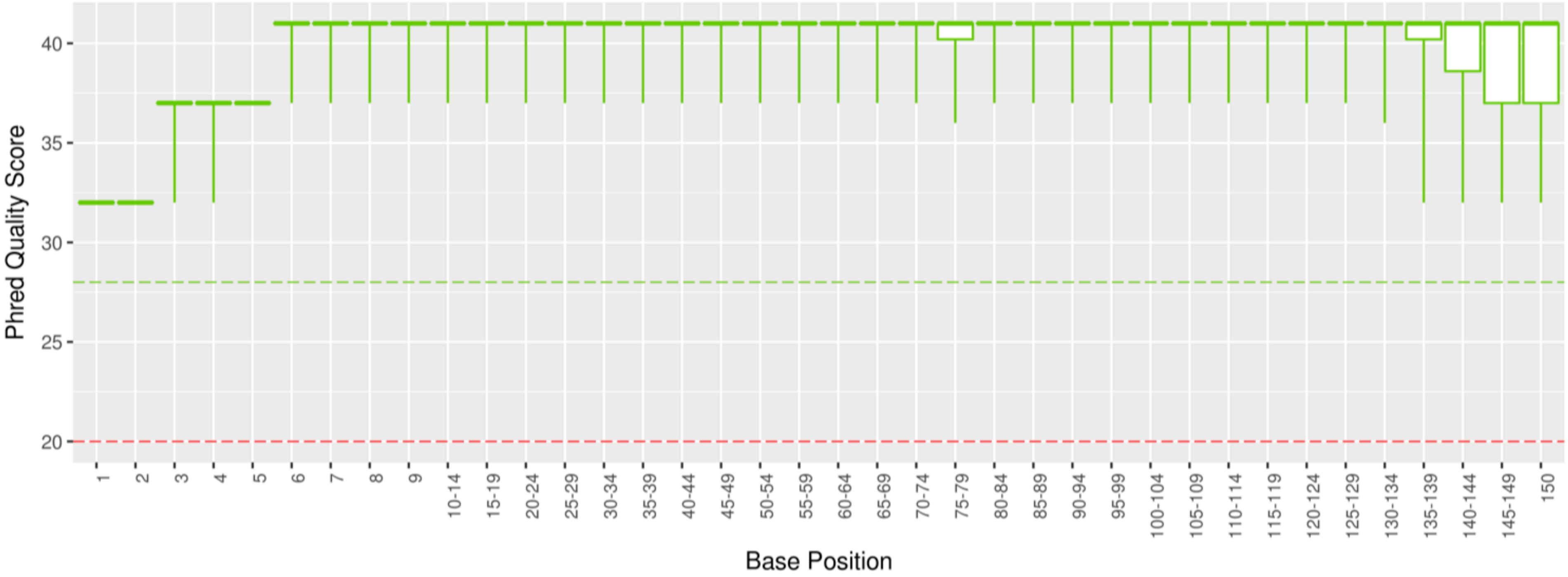

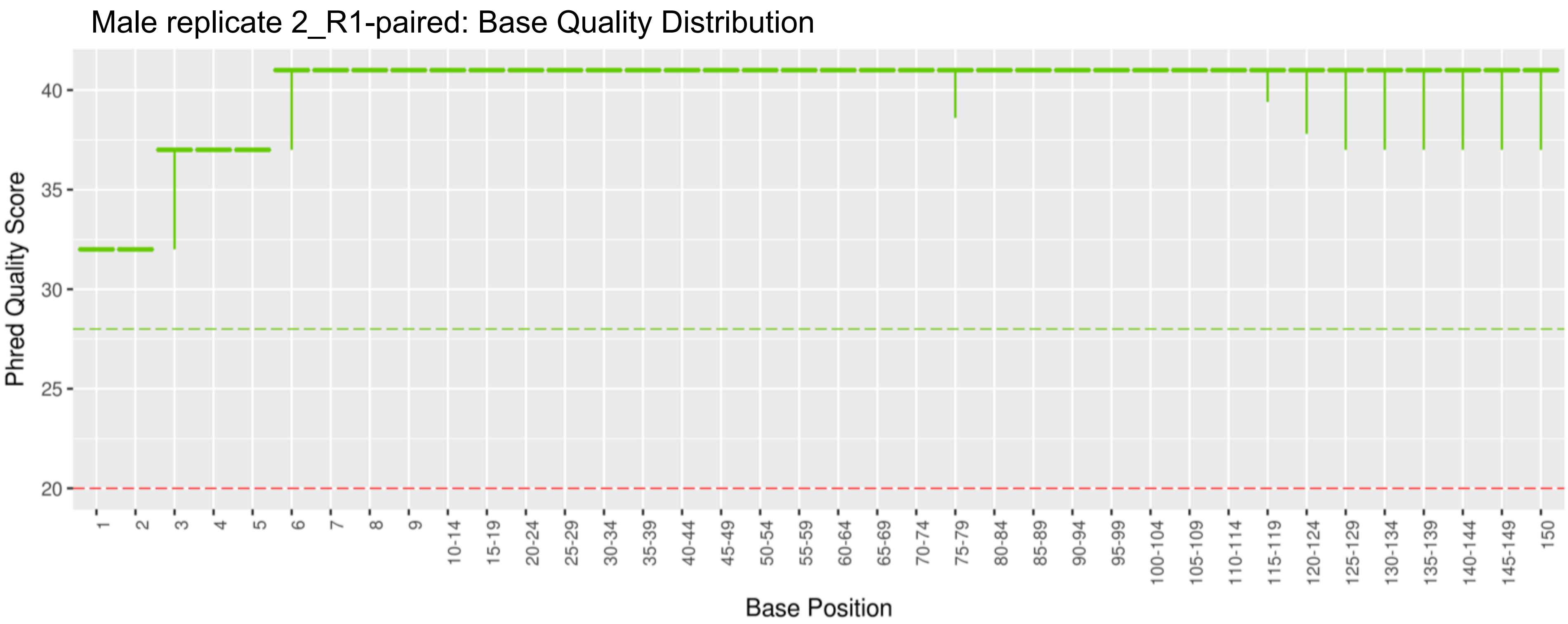

Male replicate 2_R1-paired: Base Quality Distribution
Phred Quality Score
40
35
30
25
20
1
2
3
4
5
6
7
8
9
10-14
15-19
20-24
25-29
30-34
35-39
40-44
45-49
50-54
55-59
60-64
65-69
70-74
75-79
80-84
85-89
90-94
95-99
100-104
105-109
110-114
115-119
120-124
125-129
130-134
135-139
140-144
145-149
150
Base Position

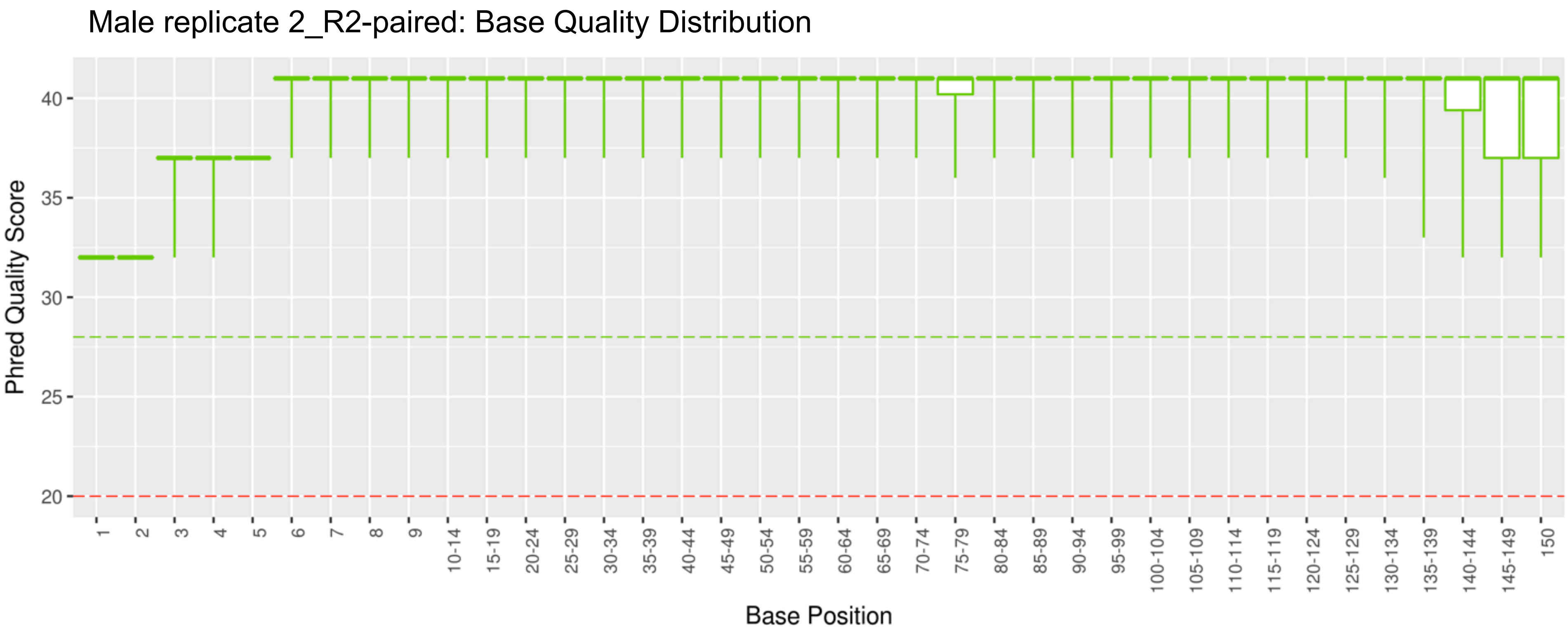
Male replicate 2_R2-paired: Base Quality Distribution
Phred Quality Score
40
35
30
25
20
1
2
3
4
5
6
7
8
9
10-14
15-19
20-24
25-29
30-34
35-39
40-44
45-49
50-54
55-59
60-64
65-69
70-74
75-79
80-84
85-89
90-94
95-99
100-104
105-109
110-114
115-119
120-124
125-129
130-134
135-139
140-144
145-149
150
Base Position

Male replicate 3_R1-paired: Base Quality Distribution

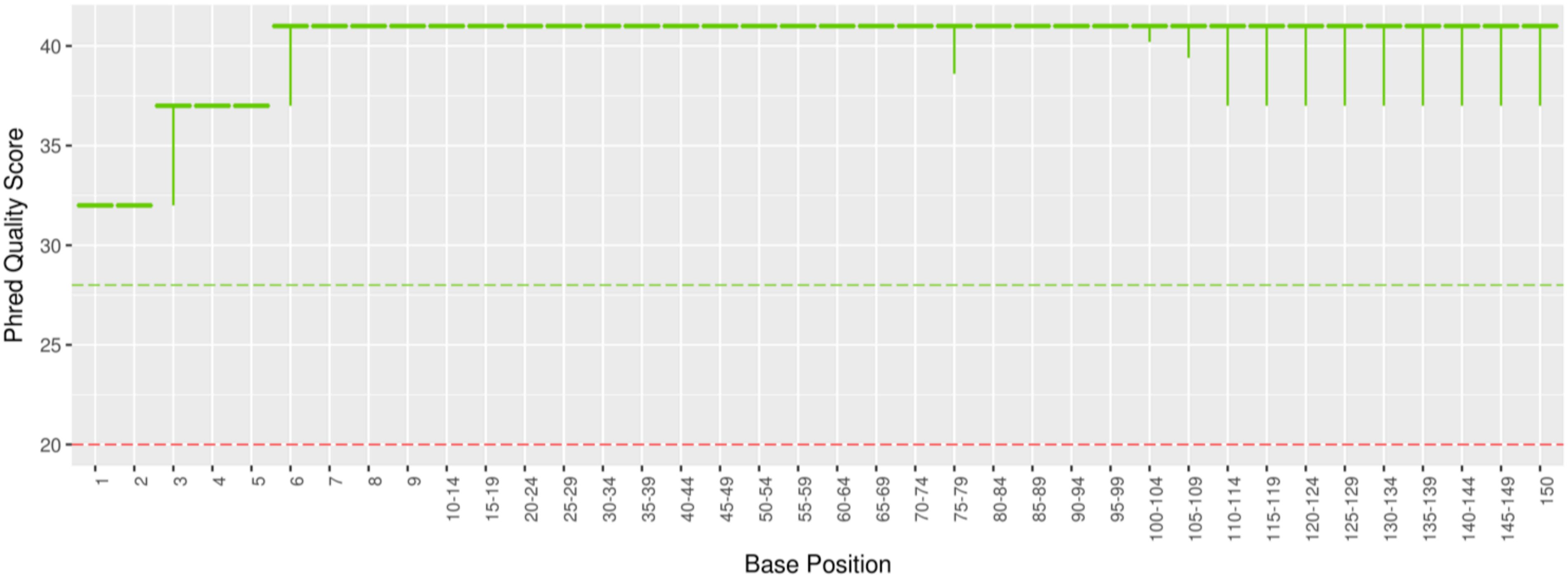

Male replicate 3_R2-paired: Base Quality Distribution

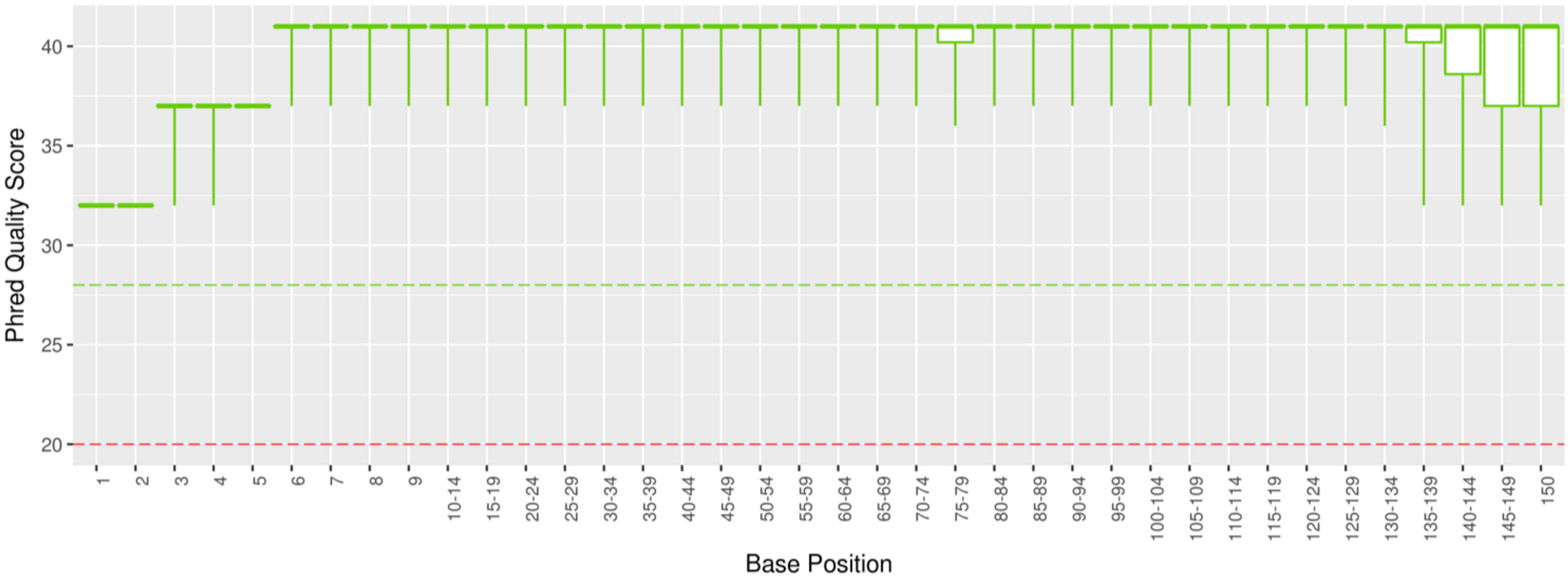

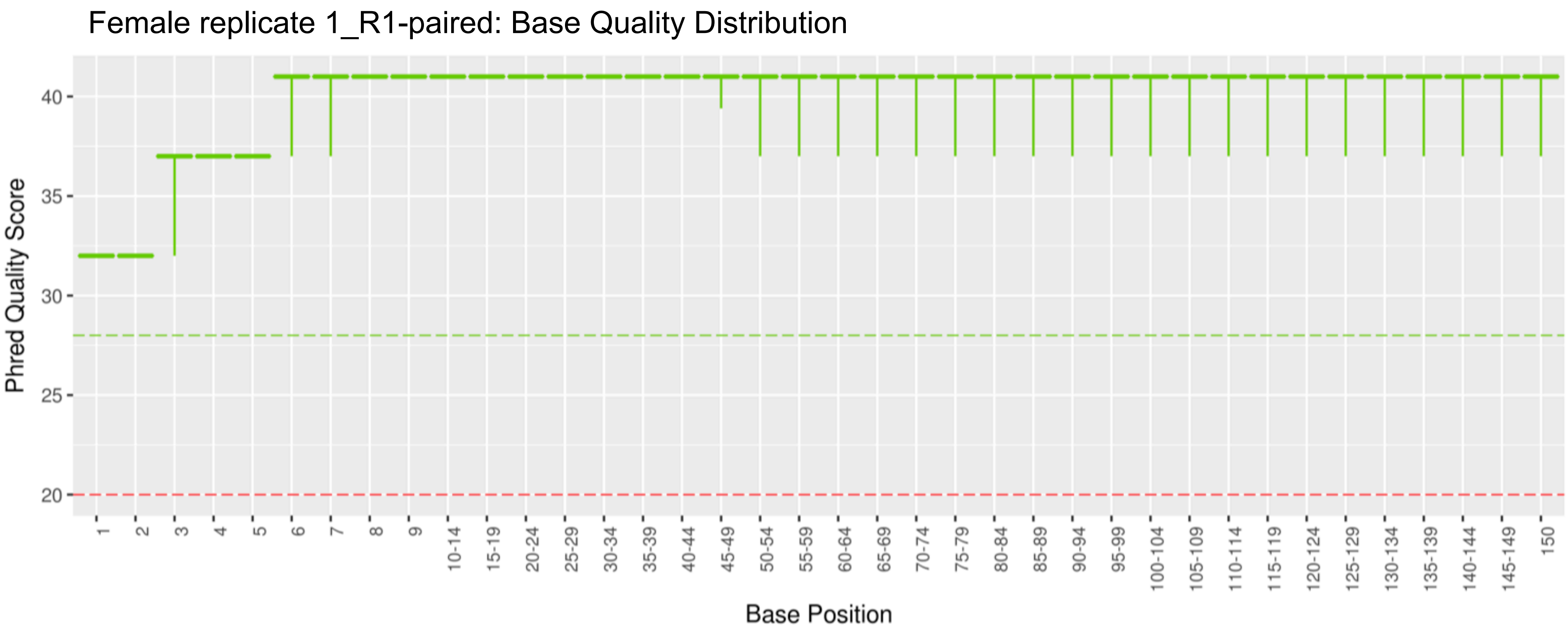

Female replicate 1_R1-paired: Base Quality Distribution
Phred Quality Score
40
35
30
25
20
1
2
3
4
5
6
7
8
9
10-14
15-19
20-24
25-29
30-34
35-39
40-44
45-49
50-54
55-59
60-64
65-69
70-74
75-79
80-84
85-89
90-94
95-99
100-104
105-109
110-114
115-119
120-124
125-129
130-134
135-139
140-144
145-149
150
Base Position

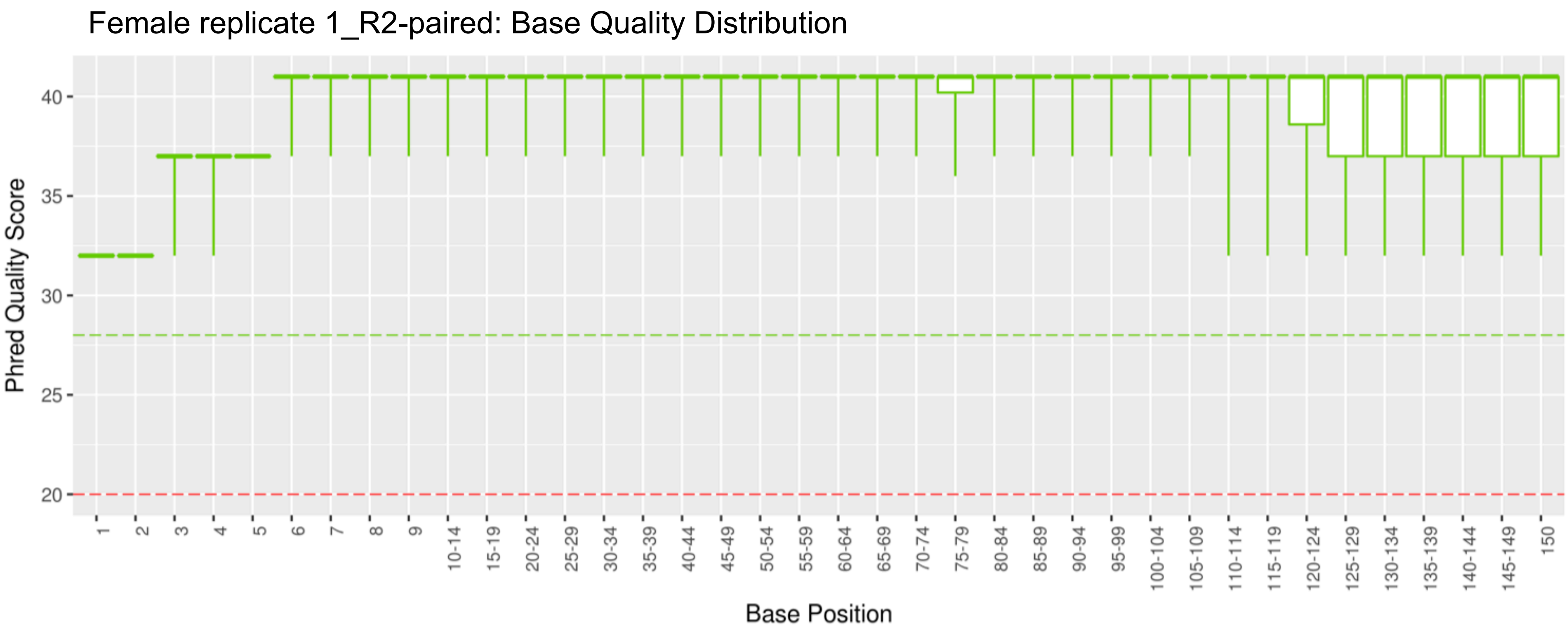

Female replicate 1_R2-paired: Base Quality Distribution
Phred Quality Score
40
35
30
25
20
1
2
3
4
5
6
7
8
9
10-14
15-19
20-24
25-29
30-34
35-39
40-44
45-49
50-54
55-59
60-64
65-69
70-74
75-79
80-84
85-89
90-94
95-99
100-104
105-109
110-114
115-119
120-124
125-129
130-134
135-139
140-144
145-149
150
Base Position

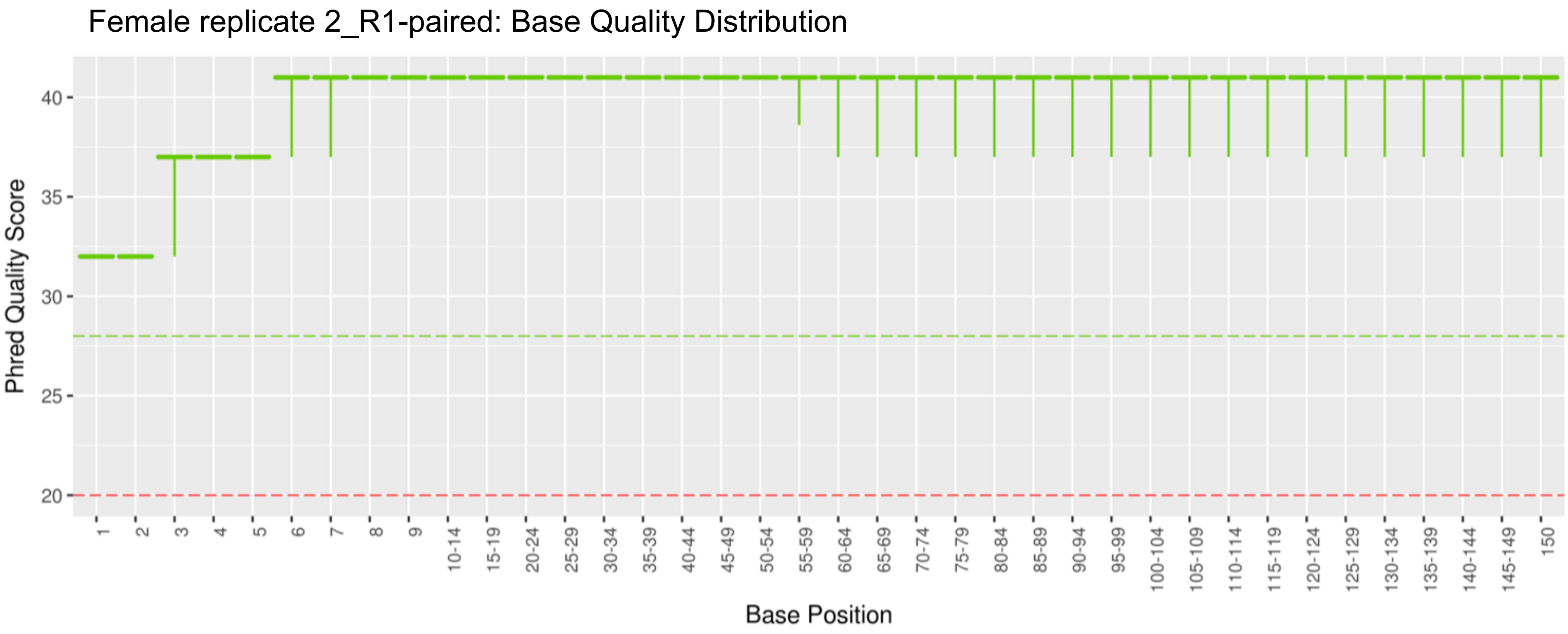

Female replicate 2_R1-paired: Base Quality Distribution
Phred Quality Score
40
35
30
25
20
1
2
3
4
5
6
7
8
9
10-14
15-19
20-24
25-29
30-34
35-39
40-44
45-49
50-54
55-59
60-64
65-69
70-74
75-79
80-84
85-89
90-94
95-99
100-104
105-109
110-114
115-119
120-124
125-129
130-134
135-139
140-144
145-149
150
Base Position

Female replicate 2_R2-paired: Base Quality Distribution

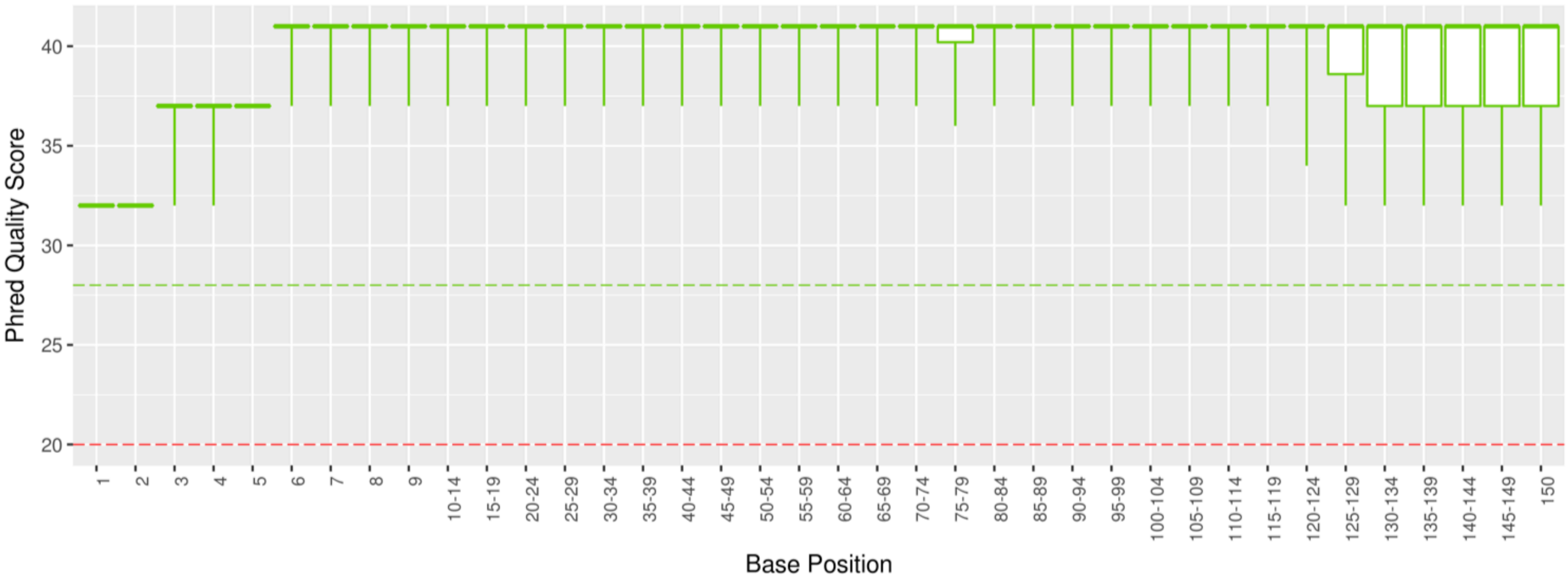

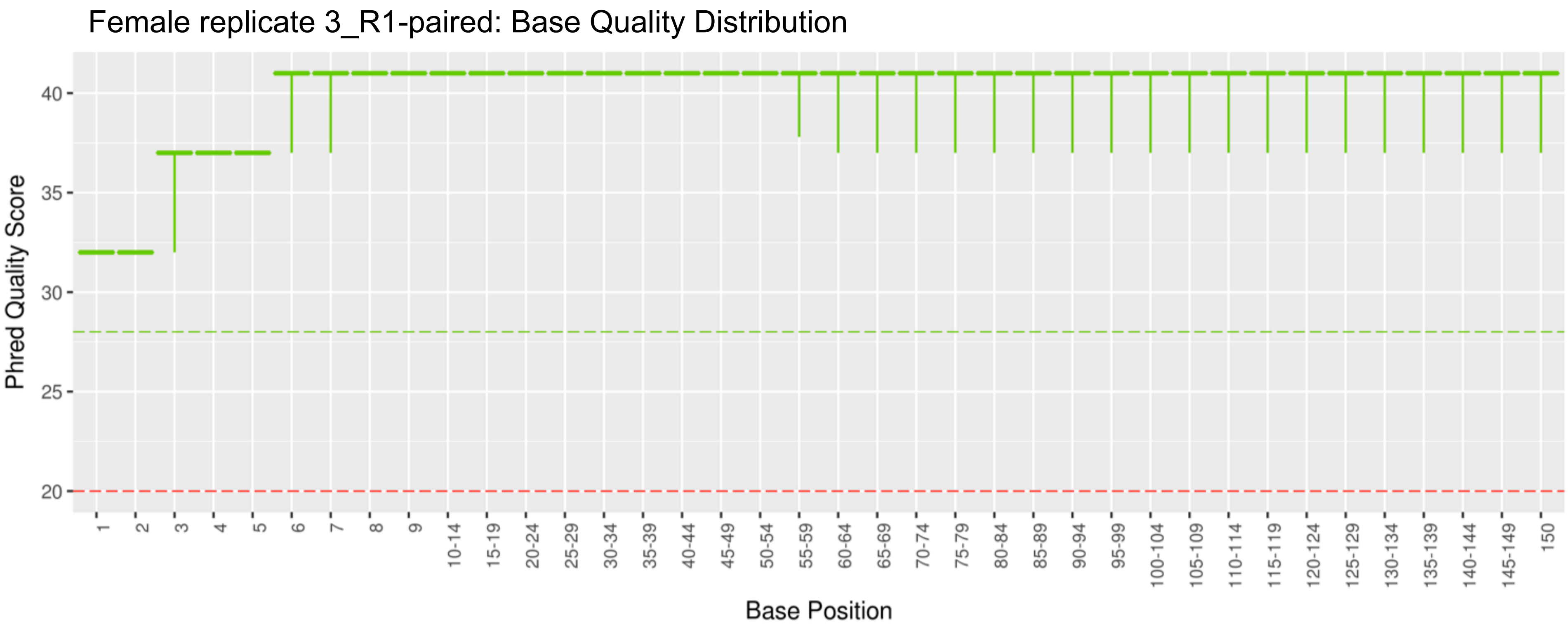
Female replicate 3_R1-paired: Base Quality Distribution
Phred Quality Score
40
35
30
25
20
1
2
3
4
5
6
7
8
9
10-14
15-19
20-24
25-29
30-34
35-39
40-44
45-49
50-54
55-59
60-64
65-69
70-74
75-79
80-84
85-89
90-94
95-99
100-104
105-109
110-114
115-119
120-124
125-129
130-134
135-139
140-144
145-149
150
Base Position

Female replicate 3_R2-paired: Base Quality Distribution

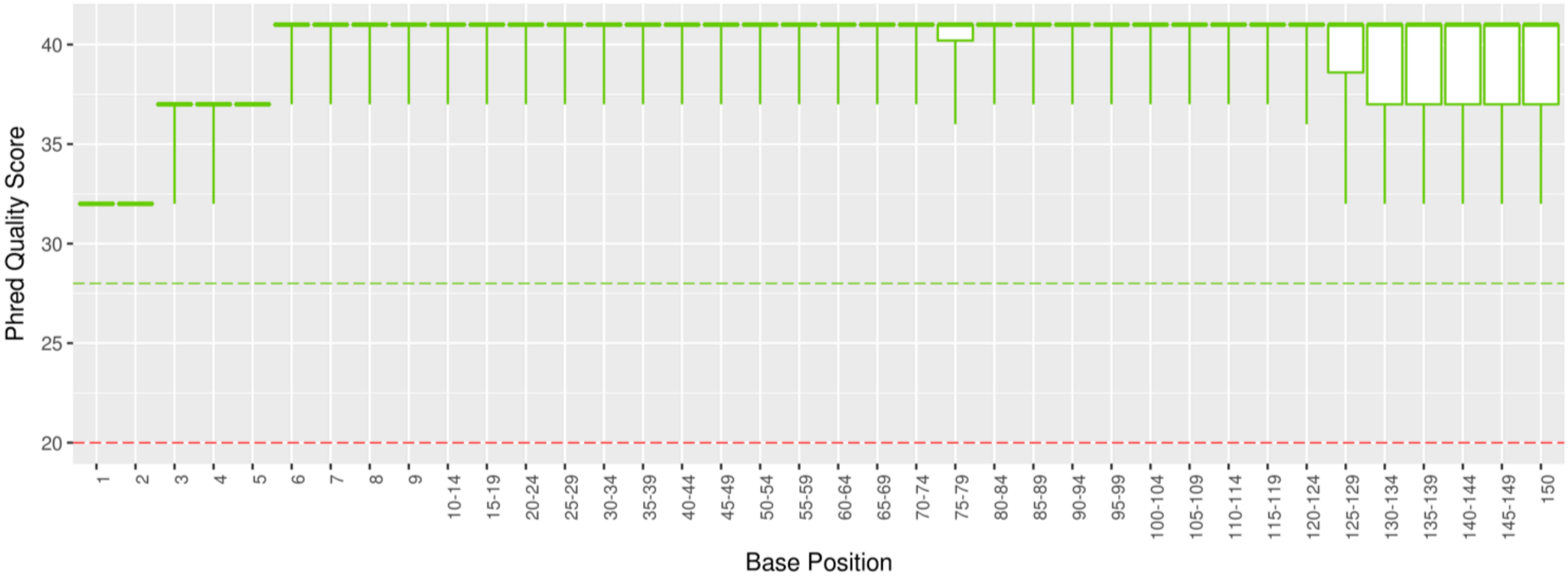

Supplement: Supplementary file 1 — Figure S1. Base quality distribution of raw reads and trimmed paired-end reads. (PDF 2840 kb) [file 13071_2018_3086_MOESM1_ESM.pdf]
