# Supplementary material for: Transcriptomic analysis of male and female Schistosoma mekongi adult worms
Source: Parasit Vectors. 2018 Sep 10;11:504. doi: 10.1186/s13071-018-3086-z (PMC6131826; doi:10.1186/s13071-018-3086-z)
Supplement: Supplementary file 6 — Figure S2. Bar graph for GO term analysis in male upregulated transcripts. (PDF 1856 kb) [file 13071_2018_3086_MOESM6_ESM.pdf]

# Additional file 6: Figure S2
# Gene Ontology Analysis

Bar graph for GO term analysis in male up-regulated transcripts

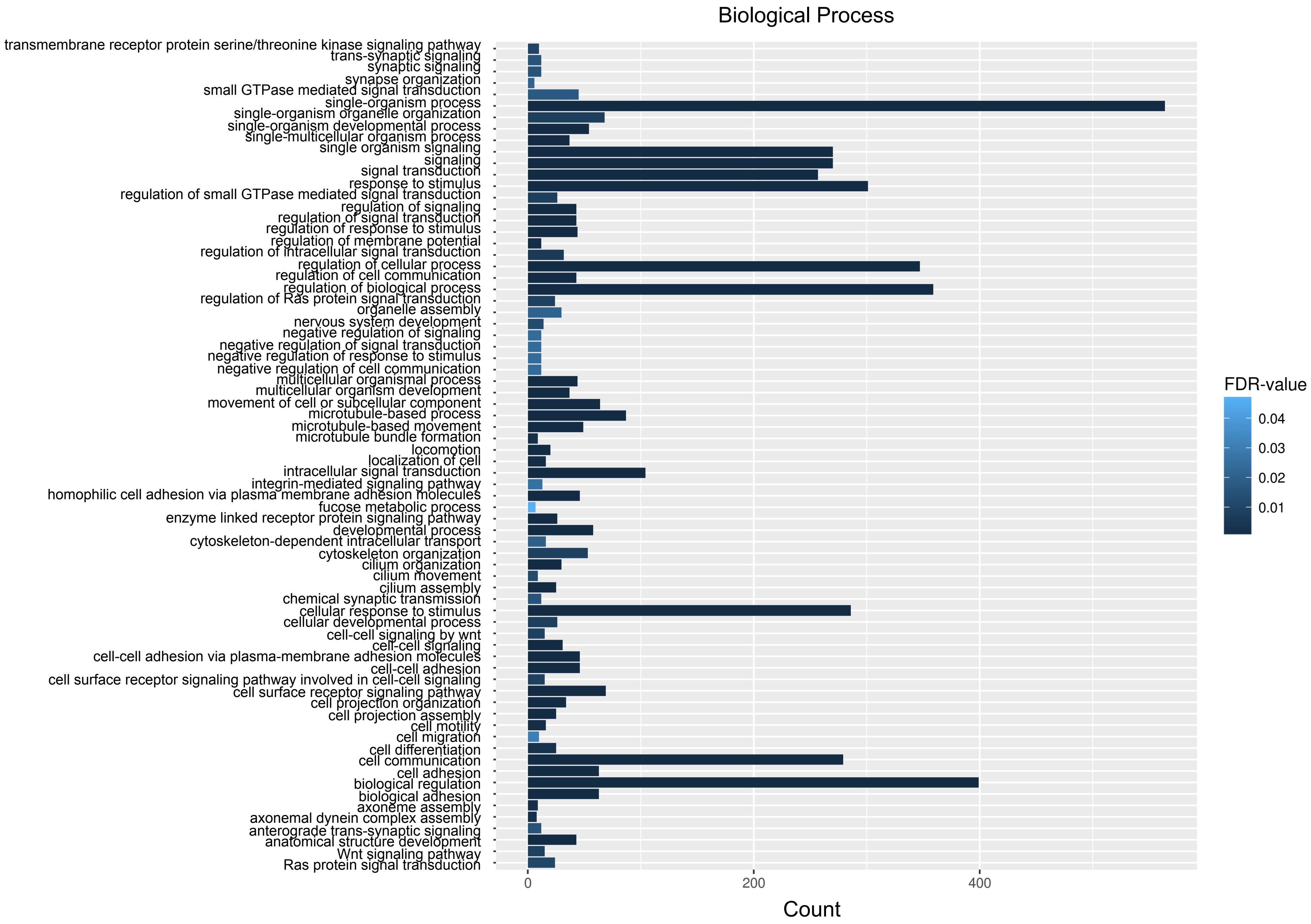
Biological Process
transmembrane receptor protein serine/threonine kinase signaling pathway
trans-synaptic signaling
synaptic signaling
synapse organization
small GTPase mediated signal transduction
single-organism process
single-organism organelle organization
single-organism developmental process
single-multicellular organism process
single organism signaling
signaling
signal transduction
response to stimulus
regulation of small GTPase mediated signal transduction
regulation of signaling
regulation of signal transduction
regulation of response to stimulus
regulation of membrane potential
regulation of intracellular signal transduction
regulation of cellular process
regulation of cell communication
regulation of biological process
regulation of Ras protein signal transduction
organelle assembly
nervous system development
negative regulation of signaling
negative regulation of signal transduction
negative regulation of response to stimulus
negative regulation of cell communication
multicellular organismal process
multicellular organism development
movement of cell or subcellular component
microtubule-based process
microtubule-based movement
microtubule bundle formation
locomotion
localization of cell
intracellular signal transduction
integrin-mediated signaling pathway
homophilic cell adhesion via plasma membrane adhesion molecules
fucose metabolic process
enzyme linked receptor protein signaling pathway
developmental process
cytoskeleton-dependent intracellular transport
cytoskeleton organization
cilium organization
cilium movement
cilium assembly
chemical synaptic transmission
cellular response to stimulus
cellular developmental process
cell-cell signaling by wnt
cell-cell signaling
cell-cell adhesion via plasma-membrane adhesion molecules
cell-cell adhesion
cell surface receptor signaling pathway involved in cell-cell signaling
cell surface receptor signaling pathway
cell projection organization
cell projection assembly
cell motility
cell migration
cell differentiation
cell communication
cell adhesion
biological regulation
biological adhesion
axoneme assembly
axonemal dynein complex assembly
anterograde trans-synaptic signaling
anatomical structure development
Wnt signaling pathway
Ras protein signal transduction
0
200
400
Count
FDR-value
0.04
0.03
0.02
0.01

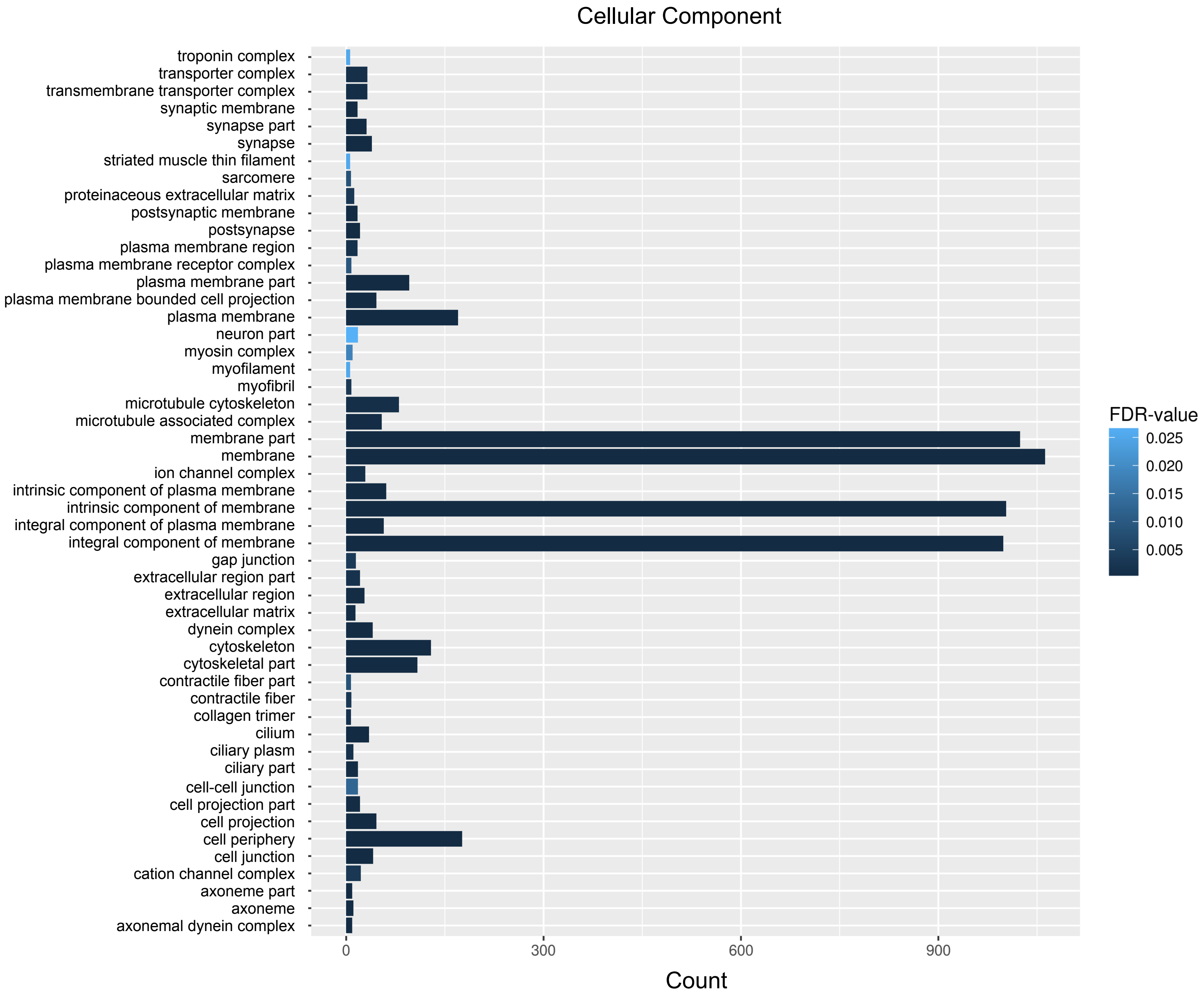

Cellular Component
troponin complex
transporter complex
transmembrane transporter complex
synaptic membrane
synapse part
synapse
striated muscle thin filament
sarcomere
proteinaceous extracellular matrix
postsynaptic membrane
postsynapse
plasma membrane region
plasma membrane receptor complex
plasma membrane part
plasma membrane bounded cell projection
plasma membrane
neuron part
myosin complex
myofilament
myofibril
microtubule cytoskeleton
microtubule associated complex
membrane part
membrane
ion channel complex
intrinsic component of plasma membrane
intrinsic component of membrane
integral component of plasma membrane
integral component of membrane
gap junction
extracellular region part
extracellular region
extracellular matrix
dynein complex
cytoskeleton
cytoskeletal part
contractile fiber part
contractile fiber
collagen trimer
cilium
ciliary plasm
ciliary part
cell-cell junction
cell projection part
cell projection
cell periphery
cell junction
cation channel complex
axoneme part
axoneme
axonemal dynein complex
0
300
600
900
Count
FDR-value
0.025
0.020
0.015
0.010
0.005

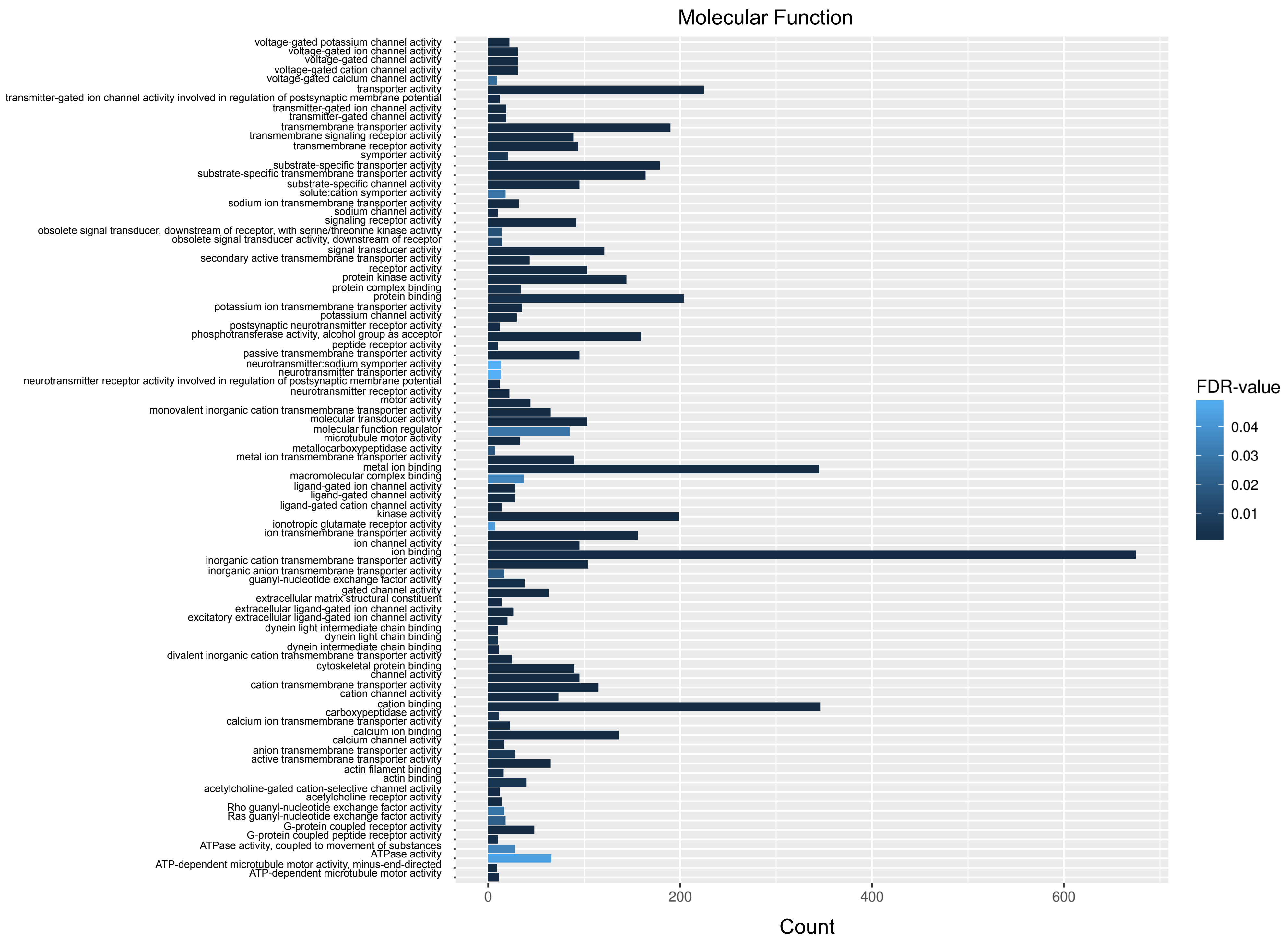

Molecular Function
voltage-gated potassium channel activity
voltage-gated ion channel activity
voltage-gated channel activity
voltage-gated cation channel activity
voltage-gated calcium channel activity
transporter activity
transmitter-gated ion channel activity involved in regulation of postsynaptic membrane potential
transmitter-gated ion channel activity
transmitter-gated channel activity
transmembrane transporter activity
transmembrane signaling receptor activity
transmembrane receptor activity
symporter activity
substrate-specific transporter activity
substrate-specific transmembrane transporter activity
substrate-specific channel activity
solute:cation symporter activity
sodium ion transmembrane transporter activity
sodium channel activity
signaling receptor activity
obsolete signal transducer, downstream of receptor, with serine/threonine kinase activity
obsolete signal transducer activity, downstream of receptor
signal transducer activity
secondary active transmembrane transporter activity
receptor activity
protein kinase activity
protein complex binding
protein binding
potassium ion transmembrane transporter activity
potassium channel activity
postsynaptic neurotransmitter receptor activity
phosphotransferase activity, alcohol group as acceptor
peptide receptor activity
passive transmembrane transporter activity
neurotransmitter:sodium symporter activity
neurotransmitter transporter activity
neurotransmitter receptor activity involved in regulation of postsynaptic membrane potential
neurotransmitter receptor activity
motor activity
monovalent inorganic cation transmembrane transporter activity
molecular transducer activity
molecular function regulator
microtubule motor activity
metallocarboxypeptidase activity
metal ion transmembrane transporter activity
metal ion binding
macromolecular complex binding
ligand-gated ion channel activity
ligand-gated channel activity
ligand-gated cation channel activity
kinase activity
ionotropic glutamate receptor activity
ion transmembrane transporter activity
ion channel activity
ion binding
inorganic cation transmembrane transporter activity
inorganic anion transmembrane transporter activity
guanyl-nucleotide exchange factor activity
gated channel activity
extracellular matrix structural constituent
extracellular ligand-gated ion channel activity
excitatory extracellular ligand-gated ion channel activity
dynein light intermediate chain binding
dynein light chain binding
dynein intermediate chain binding
divalent inorganic cation transmembrane transporter activity
cytoskeletal protein binding
channel activity
cation transmembrane transporter activity
cation channel activity
cation binding
carboxypeptidase activity
calcium ion transmembrane transporter activity
calcium ion binding
calcium channel activity
anion transmembrane transporter activity
active transmembrane transporter activity
actin filament binding
actin binding
acetylcholine-gated cation-selective channel activity
acetylcholine receptor activity
Rho guanyl-nucleotide exchange factor activity
Ras guanyl-nucleotide exchange factor activity
G-protein coupled receptor activity
G-protein coupled peptide receptor activity
ATPase activity, coupled to movement of substances
ATPase activity
ATP-dependent microtubule motor activity, minus-end-directed
ATP-dependent microtubule motor activity
0
200
400
600
Count
FDR-value
0.04
0.03
0.02
0.01
