# Supplementary material for: Transcriptomic analysis of male and female Schistosoma mekongi adult worms
Source: Parasit Vectors. 2018 Sep 10;11:504. doi: 10.1186/s13071-018-3086-z (PMC6131826; doi:10.1186/s13071-018-3086-z)
Supplement: Supplementary file 8 — Figure S3. Bar graph for GO term analysis in female upregulated transcripts. (PDF 1341 kb) [file 13071_2018_3086_MOESM8_ESM.pdf]

Additional file 8: Figure S3
Gene Ontology Analysis

Bar graph for GO term analysis in female up-regulated transcripts

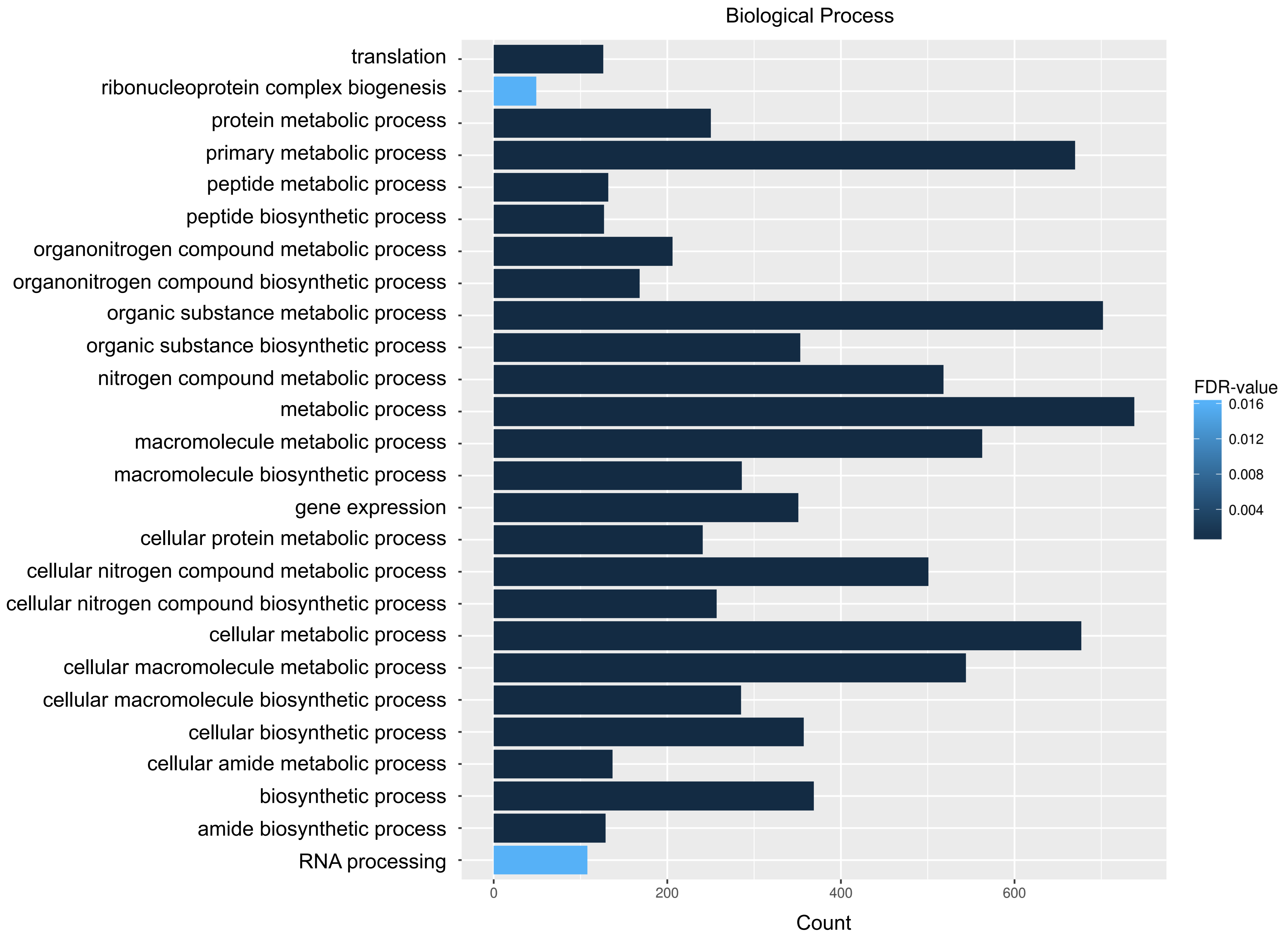
Biological Process
translation
ribonucleoprotein complex biogenesis
protein metabolic process
primary metabolic process
peptide metabolic process
peptide biosynthetic process
organonitrogen compound metabolic process
organonitrogen compound biosynthetic process
organic substance metabolic process
organic substance biosynthetic process
nitrogen compound metabolic process
metabolic process
macromolecule metabolic process
macromolecule biosynthetic process
gene expression
cellular protein metabolic process
cellular nitrogen compound metabolic process
cellular nitrogen compound biosynthetic process
cellular metabolic process
cellular macromolecule metabolic process
cellular macromolecule biosynthetic process
cellular biosynthetic process
cellular amide metabolic process
biosynthetic process
amide biosynthetic process
RNA processing
0
200
400
600
Count
FDR-value
0.016
0.012
0.008
0.004

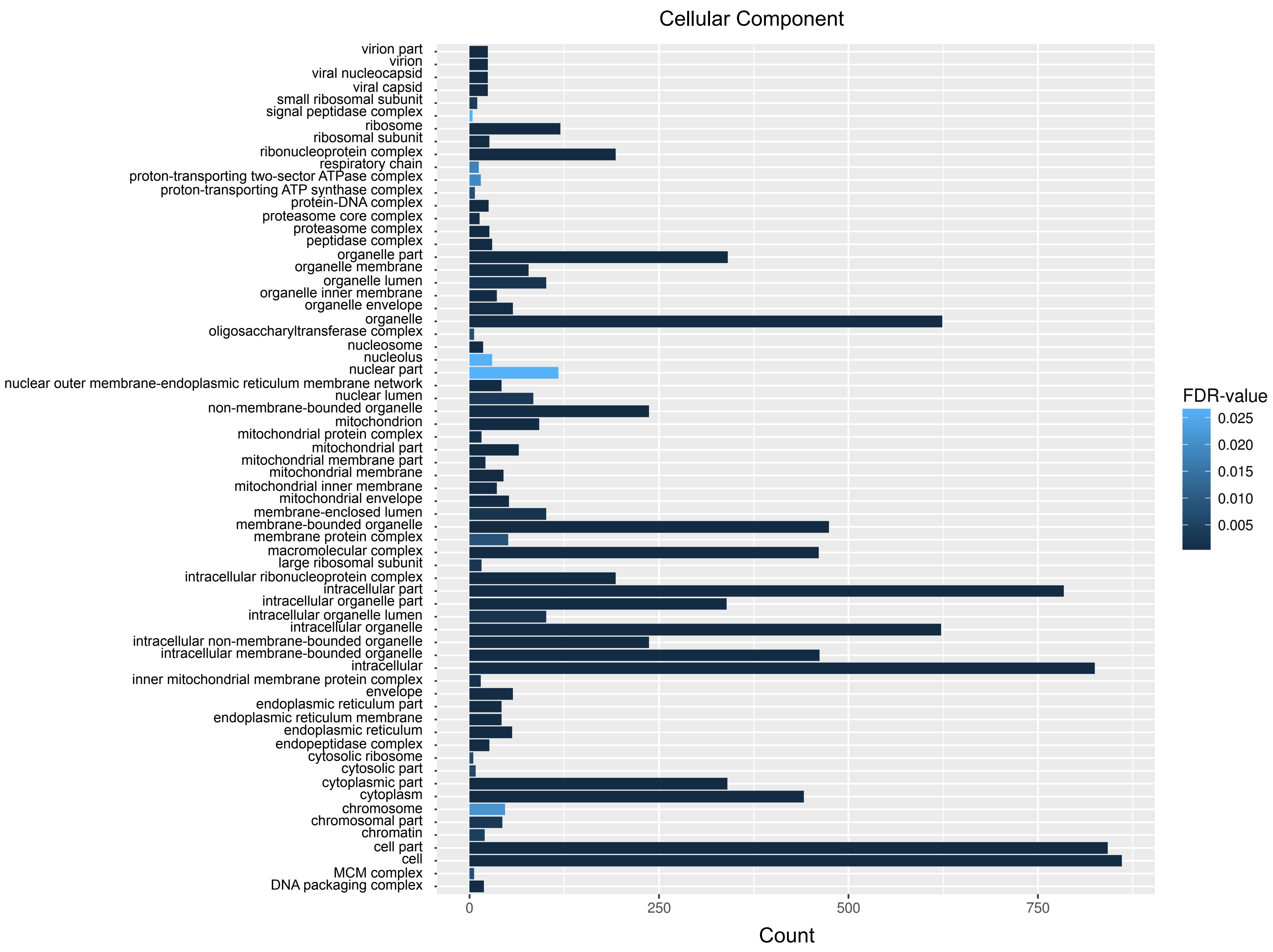
Cellular Component
virion part
virion
viral nucleocapsid
viral capsid
small ribosomal subunit
signal peptidase complex
ribosome
ribosomal subunit
ribonucleoprotein complex
respiratory chain
proton-transporting two-sector ATPase complex
proton-transporting ATP synthase complex
protein-DNA complex
proteasome core complex
proteasome complex
peptidase complex
organelle part
organelle membrane
organelle lumen
organelle inner membrane
organelle envelope
organelle
oligosaccharyltransferase complex
nucleosome
nucleolus
nuclear part
nuclear outer membrane-endoplasmic reticulum membrane network
nuclear lumen
non-membrane-bounded organelle
mitochondrion
mitochondrial protein complex
mitochondrial part
mitochondrial membrane part
mitochondrial membrane
mitochondrial inner membrane
mitochondrial envelope
membrane-enclosed lumen
membrane-bounded organelle
membrane protein complex
macromolecular complex
large ribosomal subunit
intracellular ribonucleoprotein complex
intracellular part
intracellular organelle part
intracellular organelle lumen
intracellular organelle
intracellular non-membrane-bounded organelle
intracellular membrane-bounded organelle
intracellular
inner mitochondrial membrane protein complex
envelope
endoplasmic reticulum part
endoplasmic reticulum membrane
endoplasmic reticulum
endopeptidase complex
cytosolic ribosome
cytosolic part
cytoplasmic part
cytoplasm
chromosome
chromosomal part
chromatin
cell part
cell
MCM complex
DNA packaging complex
0
250
500
750
Count
FDR-value
0.025
0.020
0.015
0.010
0.005

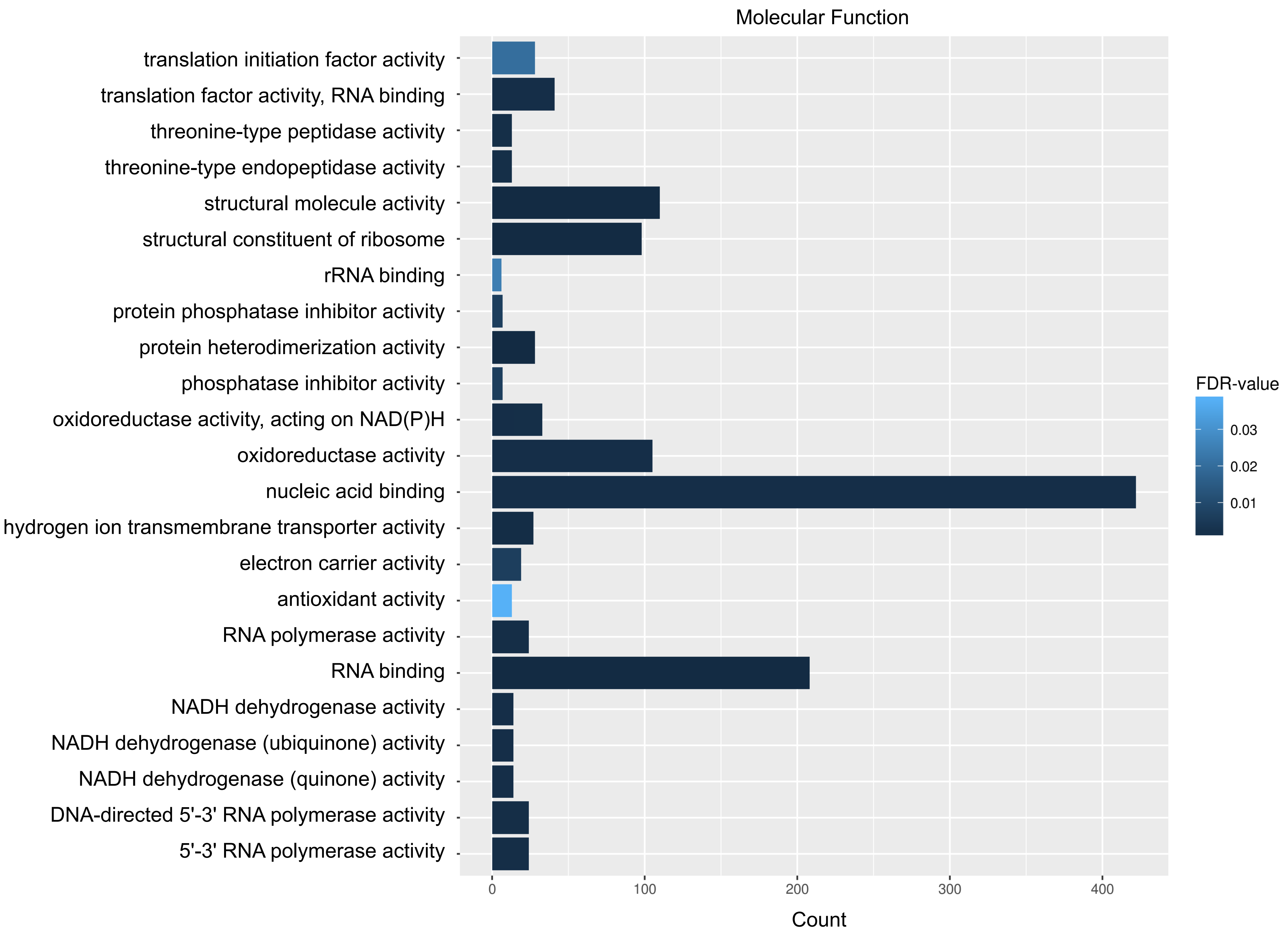
Molecular Function
translation initiation factor activity
translation factor activity, RNA binding
threonine-type peptidase activity
threonine-type endopeptidase activity
structural molecule activity
structural constituent of ribosome
rRNA binding
protein phosphatase inhibitor activity
protein heterodimerization activity
phosphatase inhibitor activity
oxidoreductase activity, acting on NAD(P)H
oxidoreductase activity
nucleic acid binding
hydrogen ion transmembrane transporter activity
electron carrier activity
antioxidant activity
RNA polymerase activity
RNA binding
NADH dehydrogenase activity
NADH dehydrogenase (ubiquinone) activity
NADH dehydrogenase (quinone) activity
DNA-directed 5'-3' RNA polymerase activity
5'-3' RNA polymerase activity
0
100
200
300
400
Count
FDR-value
0.03
0.02
0.01
