# Supplementary material for: Transcriptomic analysis of male and female Schistosoma mekongi adult worms
Source: Parasit Vectors. 2018 Sep 10;11:504. doi: 10.1186/s13071-018-3086-z (PMC6131826; doi:10.1186/s13071-018-3086-z)
Supplement: Supplementary file 12 — Figure S4. Correlation between RNA-Seq and RT-qPCR results. (PDF 90 kb) [file 13071_2018_3086_MOESM12_ESM.pdf]

Additional file 12: Figure S4

Correlation between RNA-Seq and RT-qPCR results

Correlation between RNA-Seq and RT-qPCR results for 10 male-associated DE transcripts using Spearman's rho

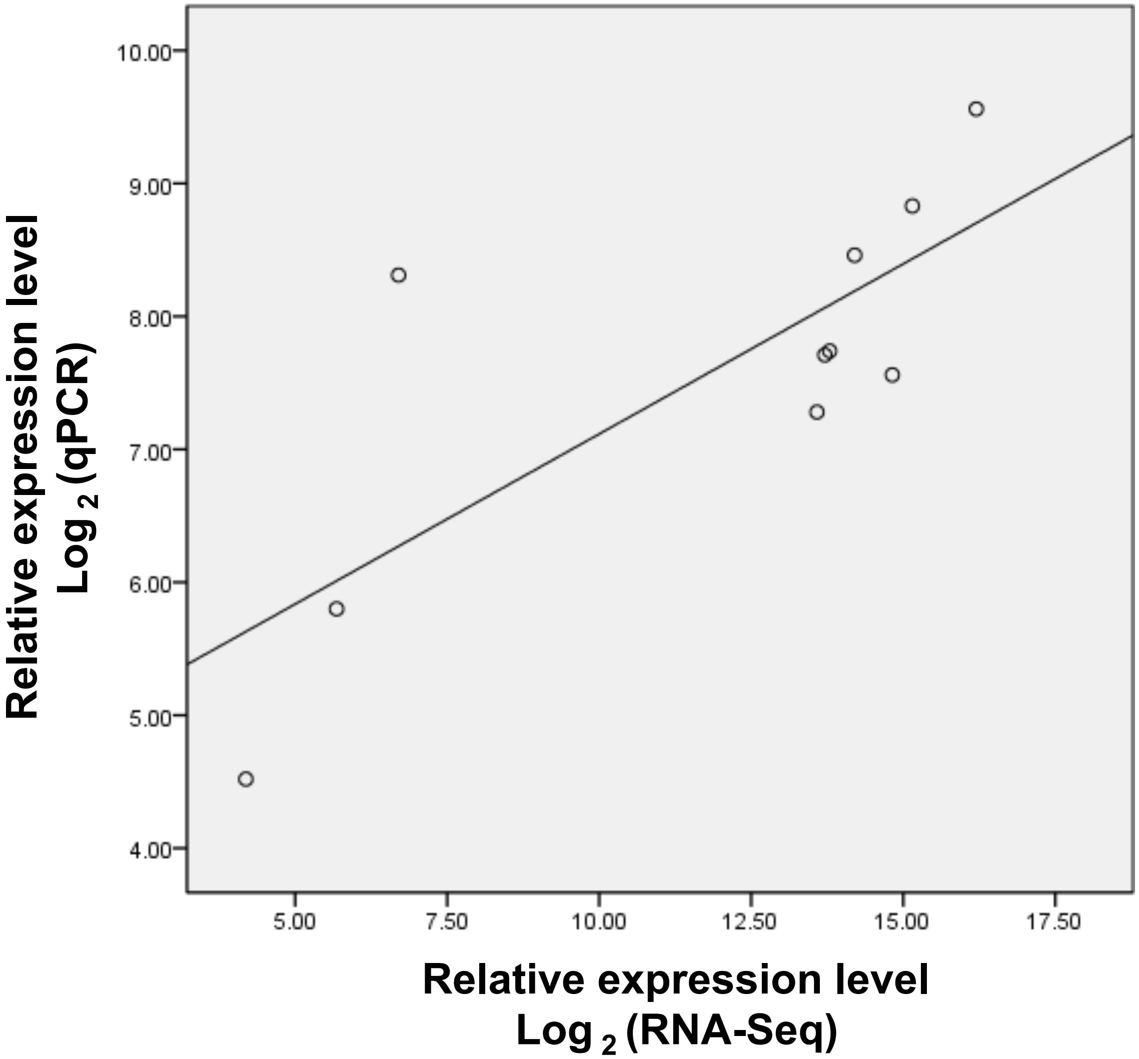

r = 0.793939
p = 0.006100

Correlation between RNA-Seq and RT-qPCR results for 10 female-associated DE transcripts using Spearman's rho

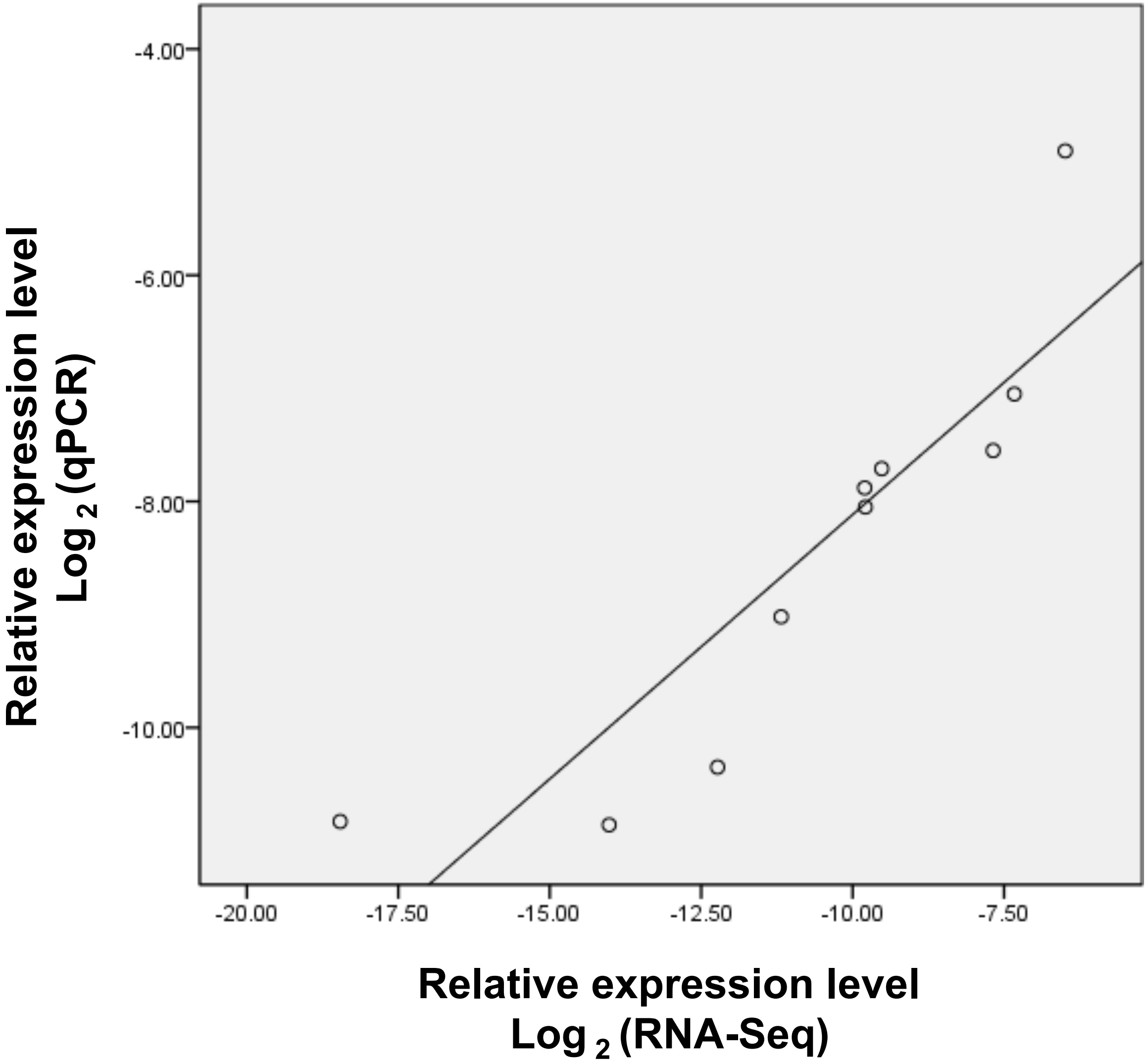

r = 0.975758
p = 0.000001

Correlation between RNA-Seq and RT-qPCR results for 10 non-DE transcripts using Spearman's rho

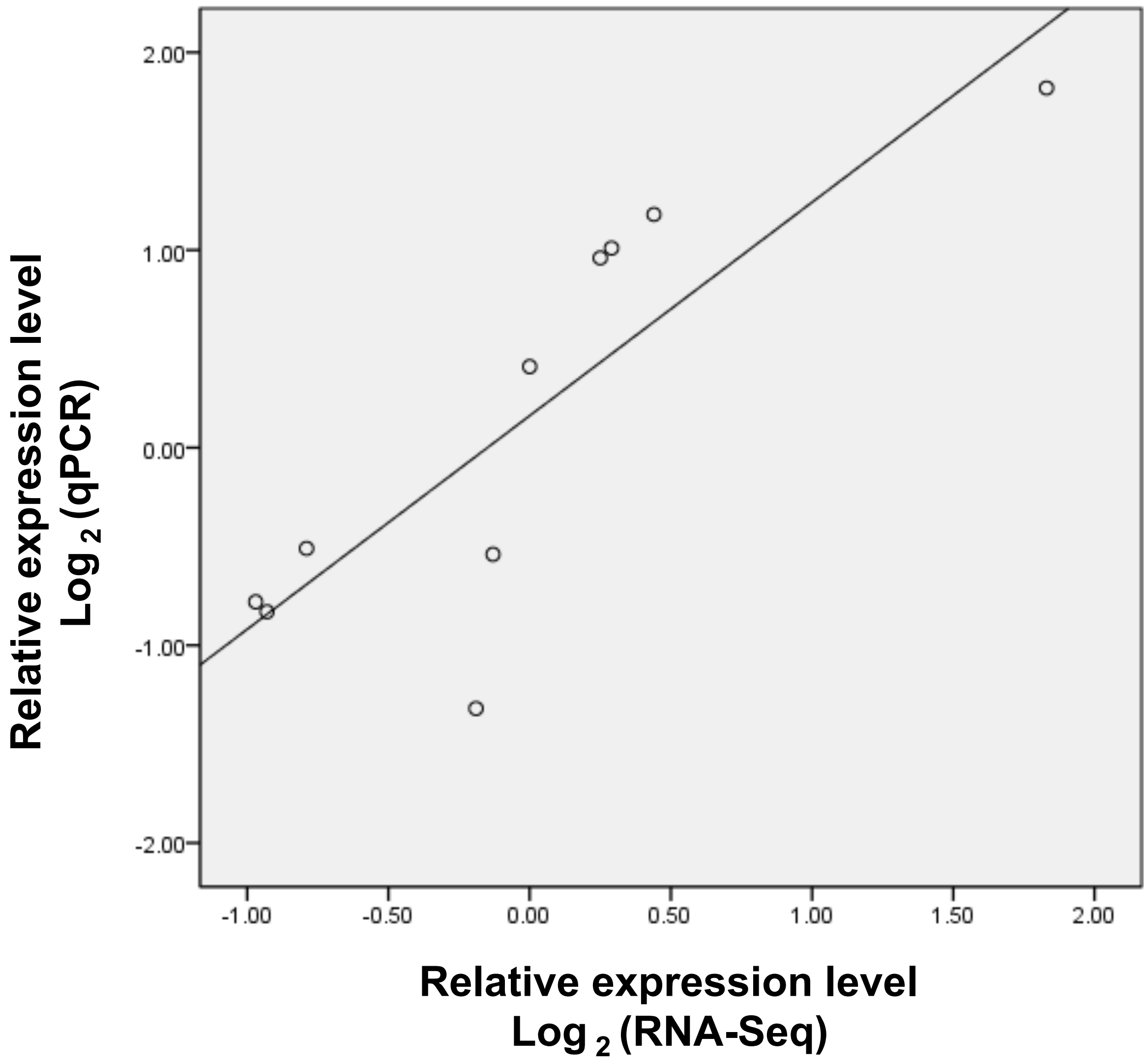

r = 0.890909
p = 0.000542
