# Supplementary material for: Transcriptomic analysis of male and female Schistosoma mekongi adult worms
Source: Parasit Vectors. 2018 Sep 10;11:504. doi: 10.1186/s13071-018-3086-z (PMC6131826; doi:10.1186/s13071-018-3086-z)
Supplement: Supplementary file 13 — Figure S5. Relative expression level of elastase 2b in male worms, female worms and eggs. (PDF 162 kb) [file 13071_2018_3086_MOESM13_ESM.pdf]

Additional file 13: Figure S5

Relative expression level of elastase 2b
in *S. mekongi* male worms, female worms, and eggs

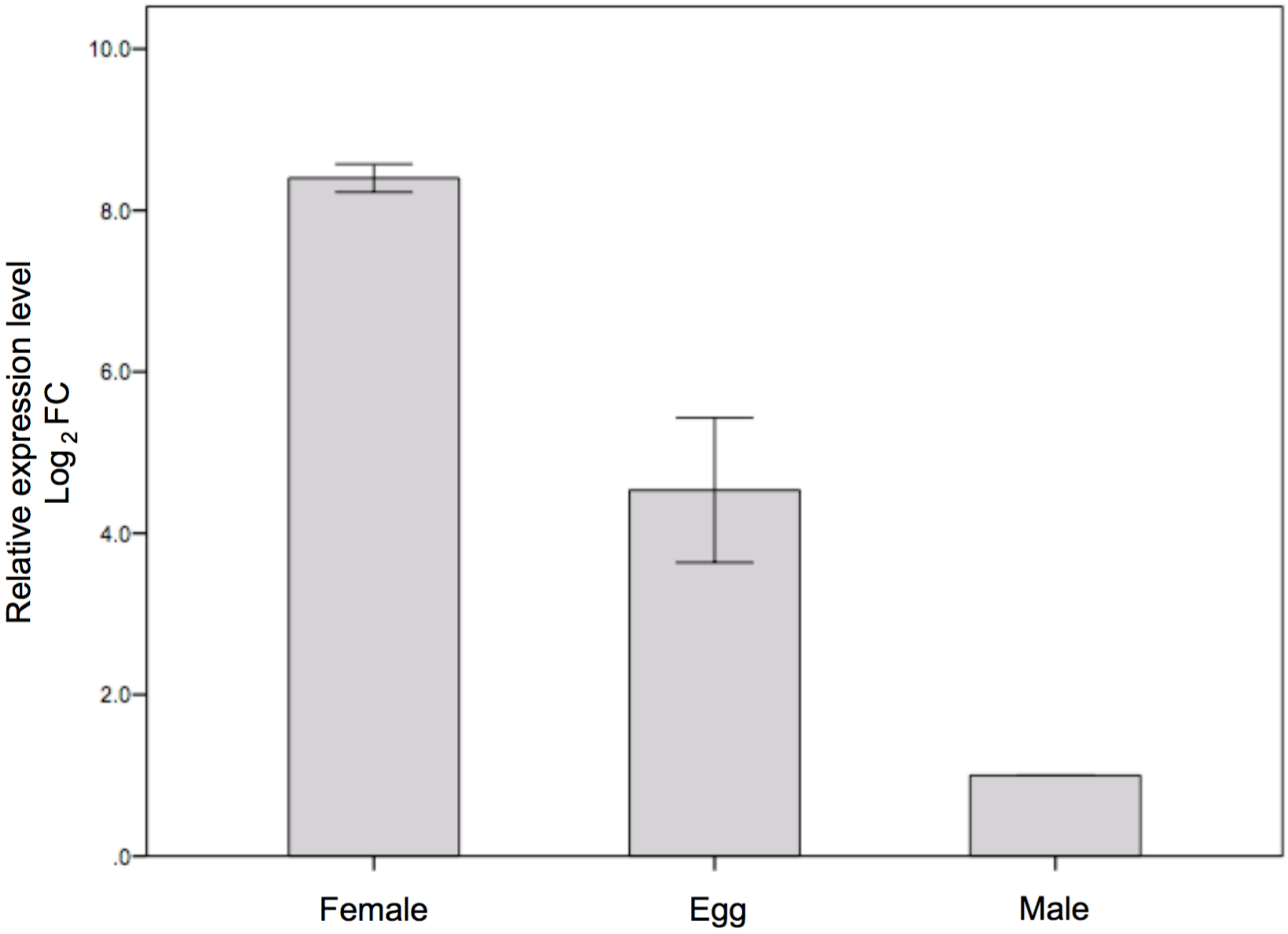

Relative expression of elastase 2b in *S. mekongi* male worms, female worms, and eggs obtained by RT-qPCR. The 18S rRNA gene was used for internal normalization among the three parasite forms. The expression level of the elastase 2b in the male worms was chosen as reference. The error bars represent the standard deviation of the mean for the three biological replicates.
