# Supplementary material for: Transcriptomic analysis of male and female Schistosoma mekongi adult worms
Source: Parasit Vectors. 2018 Sep 10;11:504. doi: 10.1186/s13071-018-3086-z (PMC6131826; doi:10.1186/s13071-018-3086-z)
Supplement: Supplementary file 14 — Figure S6. RNA integrity analysis. (PDF 1602 kb) [file 13071_2018_3086_MOESM14_ESM.pdf]

Additional file 14: Figure S6

RNA integrity analysis

Ladder

Electropherogram Summary

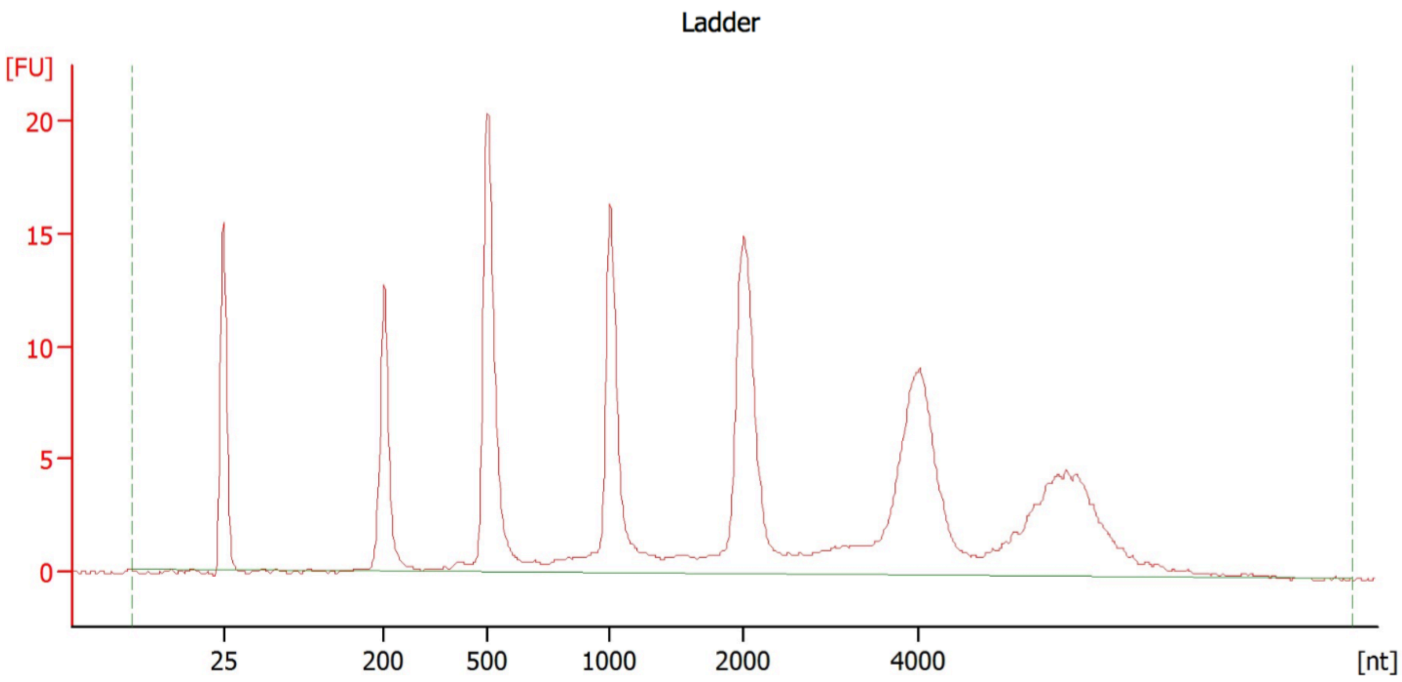

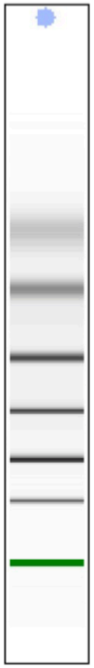

Overall Results for Ladder

RNA Area:	189.0	Result Flagging Color:	
RNA Concentration:	150 ng/µl	Result Flagging Label:	All Other Samples

Male Replicate 1

Electropherogram Summary Continued ...

Male-1 [18xdilution]

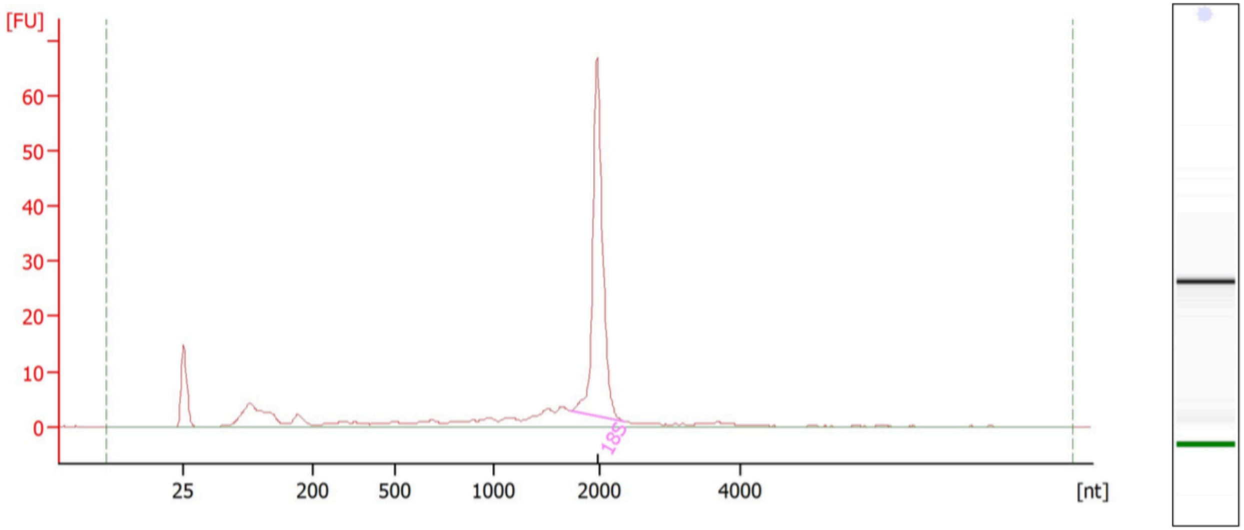

Overall Results for sample 2 : Male-1

RNA Area:	179.1	RNA Integrity Number (RIN):	8.1 (B.02.08, Anomaly Threshold(s) manually adapted)
RNA Concentration:	142 ng/µl		
rRNA Ratio [28s / 18s]:	0.0	Result Flagging Color:	
		Result Flagging Label:	RIN: 8.10

Fragment table for sample 2 : Male-1

Name	Start Size [nt]	End Size [nt]	Area	% of total Area
18S	1,729	2,352	86.1	48.1

Male Replicate 2

Electropherogram Summary Continued ...

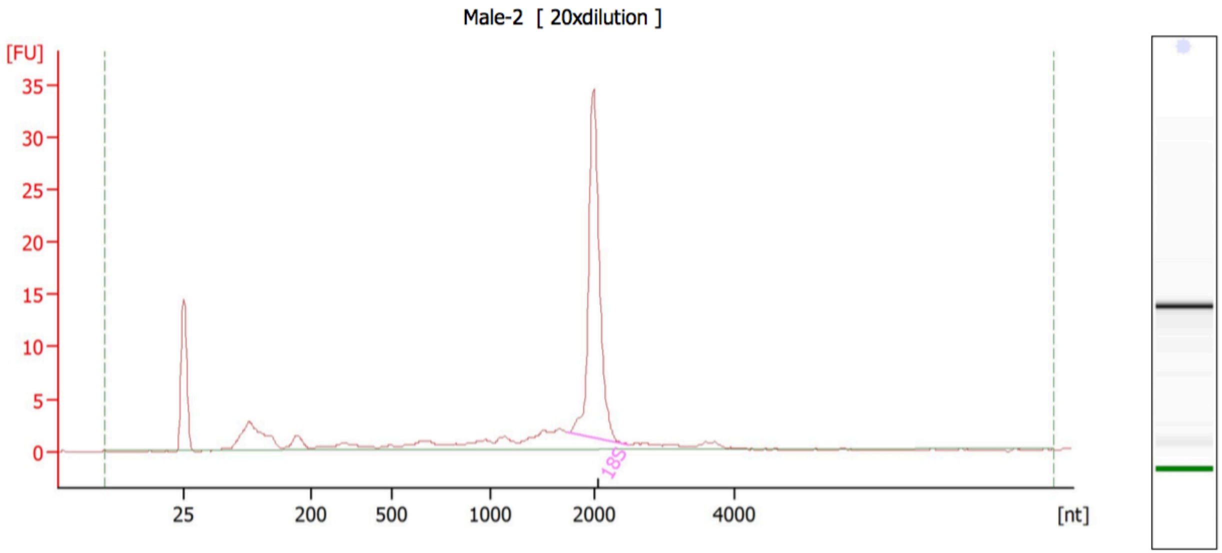

Overall Results for sample 3 : Male-2

RNA Area:	105.1	RNA Integrity Number (RIN):	7.7 (B.02.08, Anomaly Threshold(s) manually adapted)
RNA Concentration:	83 ng/µl		
rRNA Ratio [28s / 18s]:	0.0	Result Flagging Color:	
		Result Flagging Label:	RIN: 7.70

Fragment table for sample 3 : Male-2

Name	Start Size [nt]	End Size [nt]	Area	% of total Area
18S	1,742	2,491	47.4	45.1

Male Replicate 3

Electropherogram Summary Continued ...

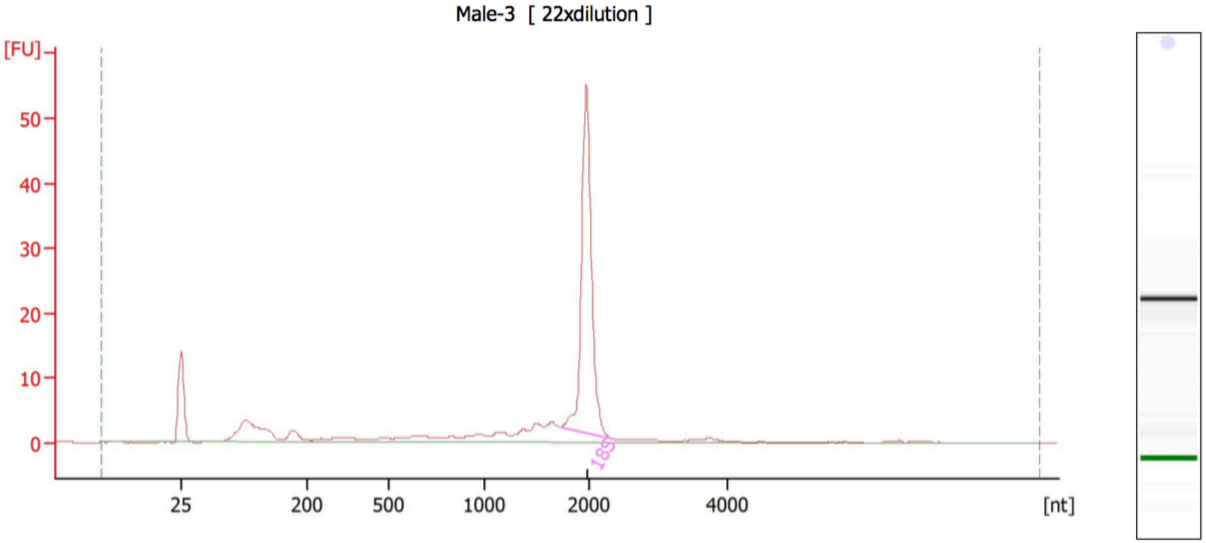

Overall Results for sample 4 : Male-3

RNA Area:	150.2	RNA Integrity Number (RIN):	7.9 (B.02.08, Anomaly Threshold(s) manually adapted)
RNA Concentration:	119 ng/µl		
rRNA Ratio [28s / 18s]:	0.0		
		Result Flagging Color:	
		Result Flagging Label:	RIN: 7.90

Fragment table for sample 4 : Male-3

Name	Start Size [nt]	End Size [nt]	Area	% of total Area
18S	1,718	2,382	71.3	47.4

Female Replicate 1

Electropherogram Summary Continued ...

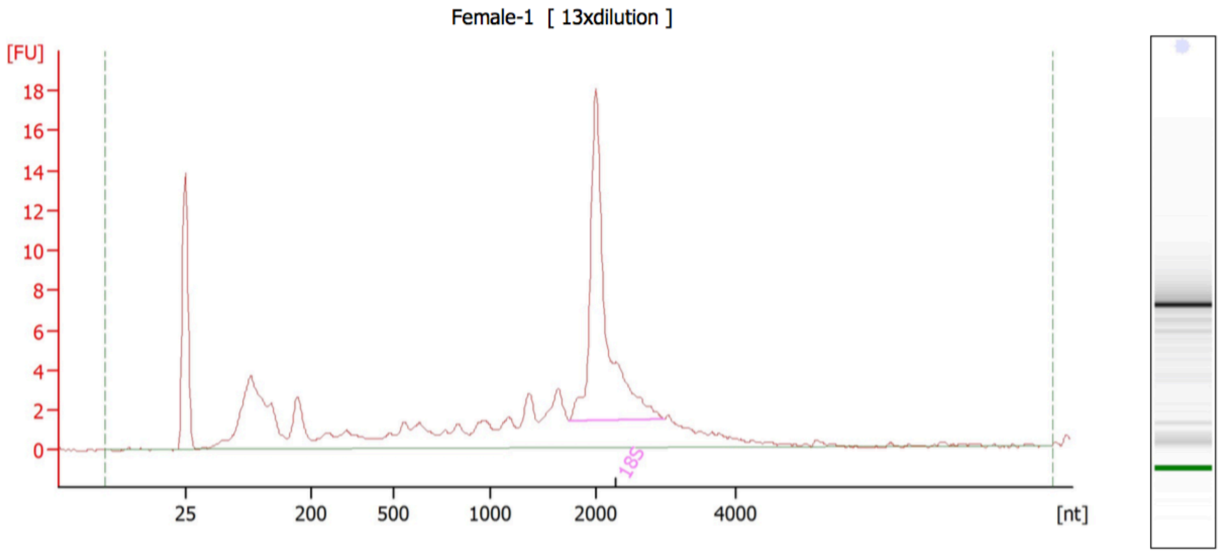

Overall Results for sample 5 : Female-1

RNA Area:	122.3	RNA Integrity Number (RIN):	7.2 (B.02.08, Anomaly Threshold(s) manually adapted)
RNA Concentration:	97 ng/µl		
rRNA Ratio [28s / 18s]:	0.0	Result Flagging Color:	
		Result Flagging Label:	RIN: 7.20

Fragment table for sample 5 : Female-1

Name	Start Size [nt]	End Size [nt]	Area	% of total Area
18S	1,734	2,971	33.8	27.6

Female Replicate 2

Electropherogram Summary Continued ...

Female-3 [6xdilution]

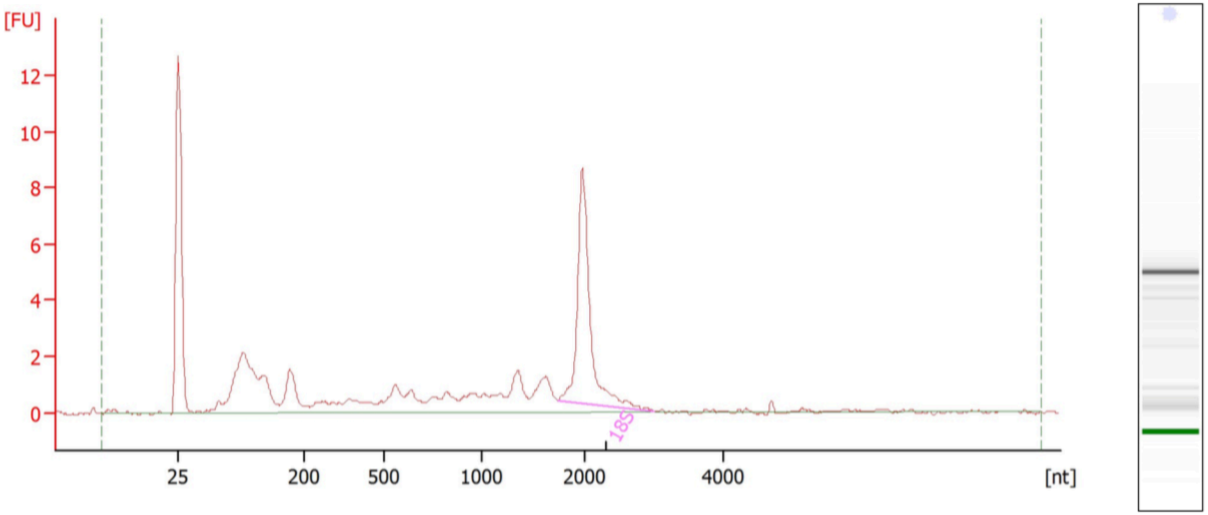

Overall Results for sample 7 : **<u>Female-3</u>**

RNA Area:	55.5	RNA Integrity Number (RIN):	7.2 (B.02.08, Anomaly Threshold(s) manually adapted)
RNA Concentration:	44 ng/µl		
rRNA Ratio [28s / 18s]:	0.0		
		Result Flagging Color:	
		Result Flagging Label:	RIN: 7.20

Fragment table for sample 7 : **<u>Female-3</u>**

Name	Start Size [nt]	End Size [nt]	Area	% of total Area
18S	1,732	3,014	14.0	25.3

Female Replicate 3

Electropherogram Summary Continued ...

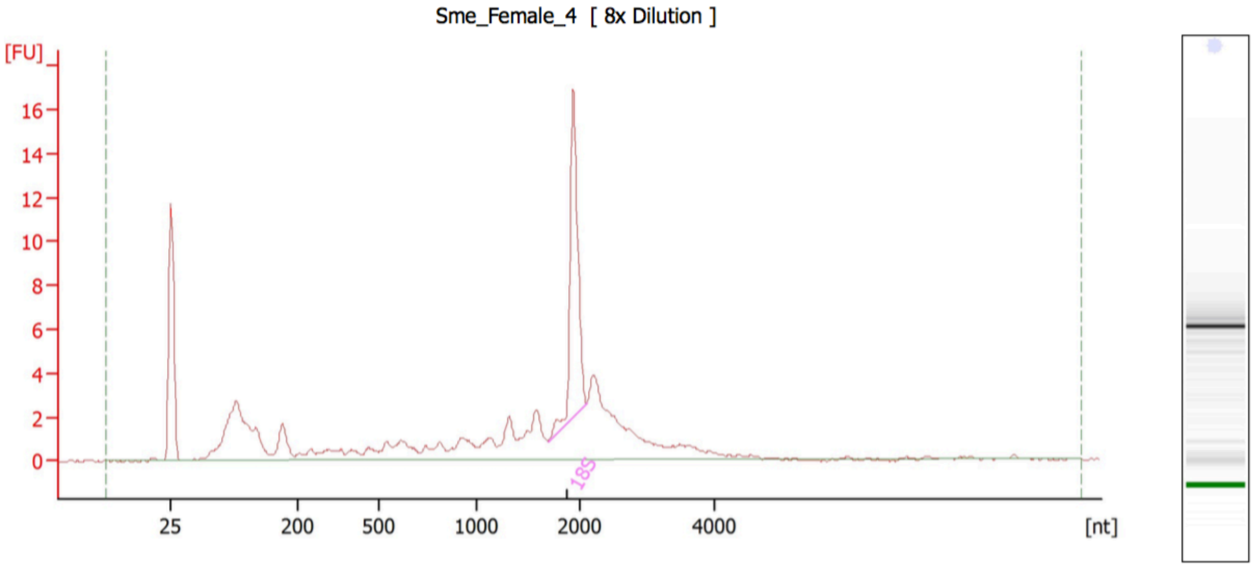

Overall Results for sample 12 : Sme_Female_4

RNA Area:	85.2	RNA Integrity Number (RIN):	6.6 (B.02.08)
RNA Concentration:	131 ng/µl	Result Flagging Color:	
rRNA Ratio [28s / 18s]:	0.0	Result Flagging Label:	RIN: 6.60

Fragment table for sample 12 : Sme_Female_4

Name	Start Size [nt]	End Size [nt]	Area	% of total Area
18S	1,692	2,097	15.7	18.5
