# Supplementary material for: Transcriptomic analysis of male and female Schistosoma mekongi adult worms
Source: Parasit Vectors. 2018 Sep 10;11:504. doi: 10.1186/s13071-018-3086-z (PMC6131826; doi:10.1186/s13071-018-3086-z)
Supplement: Supplementary file 15 — Table S9. Oligonucleotide primer sets used for RT-qPCR validation. (PDF 58 kb) [file 13071_2018_3086_MOESM15_ESM.pdf]

Additional file 15: Table S9. Oligonucleotide primer sets used for RT-qPCR validation.

No.	Transcript ID	Seq 5'- 3'	Nt	Product size (bp)
1	comp65_seq18	Fw: ATACTATCCAGCCGTTGTCCAC<br>Rw: TTGCCACTCCGTTTGTTTGC	22<br>20	119
2	comp1481_seq2	Fw: TGAGCTCGGATTTGCAGAAC<br>Rw: ACGGGCAAATTCAACCAAGG	20<br>20	112
3	comp2831_seq1	Fw: TGGTGAGACCAGAATGTTCTCC<br>Rw: ATCGTGATGCTGTTGCTGTG	22<br>20	97
4	comp926_seq0	Fw: TCGCATCTTGTTTGCACACC<br>Rw: AGGTACAAAATGCGGGCATG	20<br>20	137
5	comp1045_seq0	Fw: AACAAGTACGTGGTGGTTGG<br>Rw: ACGCCATACATTTCCAAGGC	20<br>20	86
6	comp3726_seq0	Fw: ACGCCGTGGAAAATTTGGTG<br>Rw: ATTTGCACGACTTGGTACCG	20<br>20	121
7	comp4508_seq0	Fw: AAGCCACCAATATGTGCTGAG<br>Rw: ACAGTGTTCGTGCCCATAAC	21<br>20	103
8	comp1311_seq0	Fw: AACACAACCACAAGGCAGTC<br>Rw: TAGCCTGTGGTGCAAATTGC	20<br>20	107

9	comp2062_seq0	Fw: TGCGAAAACCAGTGAAGGTG<br>Rw: AGTGCACGCTTTTTGCCTTG	20<br>20	99
10	comp4395_seq2	Fw: TCCATTTTGGCGCCATTACC<br>Rw: GCTGGTGCAGTAGGATAACAAC	20<br>22	128
11	comp2758_seq1	Fw: GTGGTGGTGGTGTTAATCTTGC<br>Rw: AGAACTTGGCACATGCATGC	22<br>20	74
12	comp4912_seq0	Fw: ATGGGTGTAGCTGATGAGGATG<br>Rw: GGCAAGAACGGCAACATTTG	22<br>20	73
13	comp2149_seq0	Fw: AACGGAAGCGTTCAAAGTGG<br>Rw: TTGAACACAGACTCGGGTTG	20<br>20	122
14	comp5896_seq0	Fw: GCAAGTCTTCAAGGTGAGTTGG<br>Rw: GTGGGGAGGTGAGGAATTTTG	22<br>21	92
15	comp837_seq1	Fw: GGCTCAATCTACTGCAGCTTTG<br>Rw: TGTGCTTTGTGCCACACTTG	22<br>20	82
16	comp5430_seq10	Fw: ACTTGCATCGGATATCCACTGG<br>Rw: TGCTCAACGCATGCTTGTTC	22<br>20	73
17	comp1134_seq0	Fw: CGCGCACGTTAAACCTTCTAG<br>Rw: AAGTGCGCATACTTCACGTG	21<br>20	114

18	comp5560_seq0	Fw: TAATGCGTCGTGTAGTCAGGTC<br>Rw: ATTGAGCCAACCATCAGACG	22<br>20	81
19	comp3298_seq0	Fw: TCTCGCCTGCGGAAATATTG<br>Rw: AGGCACGAAGAACAATTGCG	20<br>20	129
20	comp1148_seq0	Fw: ATTCCCGACGCAATGTGTTC<br>Rw: TCGCCAACCTGCATTACAAG	20<br>20	91
21	comp9311_seq2	Fw: GCGGTGGACTTTATACTCTTGG<br>Rw: TTCTCCTCCAATCGCTCCAAC	22<br>21	92
22	comp6513_seq0	Fw: TGCCTGGGAACGTGTATTTG<br>Rw: TGACAAAGCAGCAACAGCAG	20<br>20	103
23	comp7481_seq0	Fw: AAGATCCAAGGAACCGACTGTC<br>Rw: GTGCTGAAGAAGGGCTTTATGG	22<br>22	107
24	comp5964_seq1	Fw: AAAAGTGGCGCCATGAATCC<br>Rw: TCACCTCATGACTGCCCTAATG	20<br>22	137
25	comp2803_seq0	Fw: AAGCAGTCAAAGCTGGCAAC<br>Rw: AAGCCGGCGTAACTTTGTTC	20<br>20	85
26	comp7056_seq1	Fw: TCATCGTGAACTCTGCAAGC<br>Rw: TCCAGGGCAGTCAACAAGTG	20<br>20	89

27	comp4888_seq3	Fw: AACCCAGCAAAGGATAACGC<br>Rw: ACTTTGGAACTGCACGTTGG	20<br>20	100
28	comp2642_seq0	Fw: ACAGCACCACTACAGAGACATG<br>Rw: AATATTGCAGCCCGGACTTC	22<br>20	127
29	comp5540_seq1	Fw: TTGCATGACGTGAACAAGCC<br>Rw: ATGTGGCCTGTGCACAAAAC	20<br>20	89
30	comp1556_seq4	Fw: TCTGGCCTCGATGTGATATTCG<br>Rw: AAAACGTTGCGGTGAACCAC	22<br>20	93
31	18S ribosomal RNA<br>(Internal control)	Fw: TGTGCAACCTTTCAGCCGTGTC<br>Rw: AAGGCATCCGAAAGCATGCCAAC	22<br>23	102
32	comp5733_seq0<br>(Elastase 2b)	Fw: GGATTGCATTTCTCGCACTC<br>Rw: CCACCGAATGATTGTGTGAC	20<br>20	139
